# Influences on cognitive outcomes in adult patients with gliomas: A systematic review

**DOI:** 10.3389/fonc.2022.943600

**Published:** 2022-08-05

**Authors:** Matthew A. Kirkman, Benjamin H. M. Hunn, Michael S. C. Thomas, Andrew K. Tolmie

**Affiliations:** ^1^ Department of Psychology and Human Development, University College London (UCL) Institute of Education, UCL, London, United Kingdom; ^2^ Department of Neurosurgery, Queen’s Medical Centre, Nottingham University Hospitals National Health Service (NHS) Trust, Nottingham, United Kingdom; ^3^ Department of Neurosurgery, Royal Melbourne Hospital, Melbourne, VIC, Australia; ^4^ Department of Neurosurgery, Royal Hobart Hospital, Hobart, TAS, Australia; ^5^ School of Medicine, University of Tasmania, Hobart, TAS, Australia; ^6^ Department of Psychological Sciences, Birkbeck, University of London, London, United Kingdom

**Keywords:** attention, brain tumor, cognitive function, executive function, glioma, memory, outcome

## Abstract

People with brain tumors, including those previously treated, are commonly affected by a range of neurocognitive impairments involving executive function, memory, attention, and social/emotional functioning. Several factors are postulated to underlie this relationship, but evidence relating to many of these factors is conflicting and does not fully explain the variation in cognitive outcomes seen in the literature and in clinical practice. To address this, we performed a systematic literature review to identify and describe the range of factors that can influence cognitive outcomes in adult patients with gliomas. A literature search was performed of Ovid MEDLINE, PsychINFO, and PsycTESTS from commencement until September 2021. Of 9,998 articles identified through the search strategy, and an additional 39 articles identified through other sources, 142 were included in our review. The results confirmed that multiple factors influence cognitive outcomes in patients with gliomas. The effects of tumor characteristics (including location) and treatments administered are some of the most studied variables but the evidence for these is conflicting, which may be the result of methodological and study population differences. Tumor location and laterality overall appear to influence cognitive outcomes, and detection of such an effect is contingent upon administration of appropriate cognitive tests. Surgery appears to have an overall initial deleterious effect on cognition with a recovery in most cases over several months. A large body of evidence supports the adverse effects of radiotherapy on cognition, but the role of chemotherapy is less clear. To contrast, baseline cognitive status appears to be a consistent factor that influences cognitive outcomes, with worse baseline cognition at diagnosis/pre-treatment correlated with worse long-term outcomes. Similarly, much evidence indicates that anti-epileptic drugs have a negative effect on cognition and genetics also appear to have a role. Evidence regarding the effect of age on cognitive outcomes in glioma patients is conflicting, and there is insufficient evidence for gender and fatigue. Cognitive reserve, brain reserve, socioeconomic status, and several other variables discussed in this review, and their influence on cognition and recovery, have not been well-studied in the context of gliomas and are areas for focus in future research.

**Systematic Review Registration:**

https://www.crd.york.ac.uk/prospero/, identifier CRD42017072976

## Introduction

People living with, or with a history of, brain tumors are commonly affected by a range of neurocognitive impairments involving executive function, memory, attention, and social/emotional functioning (1–7) that have a profound impact on their quality of life (8) and that of their families (9). The development of cognitive deficits limits an individual’s potential to retrain or learn compensation strategies. Cognitive dysfunction has thus become an important outcome measure in treatment trials for brain tumors. Cognitive problems are not always evident to healthcare staff, with patients often appearing to be cognitively ‘normal’ in formal clinic/hospital settings (10), which reduces the opportunities for appropriate and timely referrals to treatments such as cognitive rehabilitation and support services.

Gliomas are tumors that arise in the glial cells of the central nervous system, which are non-neuronal cells that maintain homeostasis and provide support and protection for neurons. They are the most frequent type of primary brain tumor in adults (11), with an age-adjusted annual incidence of 7.3 cases per 100,000 person-years in one Danish registry study (12), and can present with a range of clinical symptoms including seizures. Gliomas are classified according to their molecular and histopathological characteristics by the World Health Organization (WHO) into grades I to IV; grade I and II gliomas have traditionally been termed ‘benign’ or ‘low-grade’, and grades III and IV ‘malignant’ or ‘high-grade’. However, this is a rather oversimplified classification and several subtypes exist (13), and the classification is continually being updated, most recently in the 2021 WHO classification (14). The majority of patients with glioma have high-grade gliomas (HGGs) as opposed to low-grade gliomas (LGGs); the ratio was 85%:15% HGG : LGG in one study (12). Survival is markedly better for patients with LGGs than those with HGGs, although inevitably LGGs ‘transform’ into HGGs over time, even with aggressive treatment of the LGG. Treatment options for patients with gliomas depend on several factors, including clinical factors (e.g., patient age, tumor type/grade, and size and location of the tumor) and patient preferences, but can include surgery, chemotherapy, and/or radiotherapy. Surgical approaches to brain tumors are heterogeneous and can include obtaining a small sample of tissue for diagnostic biopsy through to complete (or even supratotal) resection, contingent on many factors including the surgeon’s preference, patient demographics and wishes, and other clinical and treatment variables.

The reported prevalence of neurocognitive impairments in patients with gliomas varies widely across studies, but it is common; prior to any treatment, up to 75% of patients experience cognitive impairments (5, 15). There are several factors postulated to influence cognitive impairment in patients with brain tumors, including the biophysical aspects of the tumor and its associated treatments (7, 16–20), genetics (7), as well as extent of associated cerebral oedema and tumor grade (4, 5, 7). However, evidence relating to many of these factors is conflicting and does not fully explain the variation in cognitive outcomes seen both in the literature (some of which may be due to methodological differences between studies) and in clinical practice. At least some of the conflicting findings are likely to be a reflection of the complexity of the relationships between different variables and cognitive outcomes. For example, although it may be expected that tumors located in regions of the brain subserving cognitive functions would affect cognition due to disruption of the networks, tumors located elsewhere in the brain can still cause cognitive dysfunction for several reasons, including the administration of chemotherapy and radiotherapy. Factors such as education, cognitive function prior to disease onset, and sociodemographic factors are also likely to influence cognitive outcomes and the variation in such outcomes. Growth of the tumor, or treatments including surgery, may trigger plastic reorganization of the brain, with locally lost functions potentially being compensated for by other brain regions in order to achieve a desired outcome or behavior; patients with slower-growing tumours including LGGs may have sufficient ‘time’ to facilitate such functional reorganization.

To more fully understand the influences on cognitive outcomes in patients with gliomas, we performed a systematic review of the literature with the aim of providing a unified view of the range of factors that can influence cognitive outcomes in patients with gliomas.

## Methods

### Study approval

The protocol for this systematic review was reviewed and approved by the International Prospective Register of Systematic Reviews (PROSPERO; approval number CRD42017072976; https://www.crd.york.ac.uk/PROSPERO/display_record.php?ID=CRD42017072976&ID=CRD42017072976).

### Data sources and search strategy

The methods used in this systematic review were prespecified and are presented in accordance with the 2020 Preferred Reporting Items for Systematic Reviews and Meta-Analyses (PRISMA) guidelines (21). A literature search was performed using the electronic databases of Ovid MEDLINE(R) Epub Ahead of Print, In-Process & Other Non-Indexed Citations, Ovid MEDLINE(R) Daily and Ovid MEDLINE(R) (1946 to Present [April 2018]), PsycINFO (1806 to April Week 1 2018), and PsycTESTS (1910 to March 2018). A top-up search was subsequently performed with the same databases: Ovid MEDLINE(R) Epub Ahead of Print, In-Process & Other Non-Indexed Citations, Ovid MEDLINE(R) Daily and Ovid MEDLINE(R) (1946 to September 24, 2021), PsycINFO (1806 to September Week 3 2021), and PsycTESTS (1910 to September 2021), with a filter for articles published from 2018 onwards. Medical Subject Heading (MeSH) terms (**Supplementary Table 1**) were used to ensure the search was as comprehensive as possible. The search strategy that was created combined the three broad content areas of brain tumor, cognition, and outcome/recovery/plasticity (**Supplementary Table 2**). These three content areas were combined using the Boolean operator “and”. Reference lists of identified studies were also reviewed to identify additional relevant studies.

### Inclusion criteria

To be eligible for inclusion in this systematic review, the manuscripts identified had to: report primary data; include adult patients with gliomas; and be published in English language. Although the focus of this review is on patients with gliomas, the search strategy (**Supplementary Table 2**) was deliberately broad to include a range of brain tumors in order to ensure all studies incorporating patients with gliomas were identified, including studies with mixed pathologies (different types of brain tumors or brain tumor and non-brain tumor pathologies, for example). Studies evaluating interventions for the amelioration or prevention of cognitive dysfunction were identified using the search strategy but not included in this systematic review manuscript, as this manuscript focuses on non-interventional influences on cognition. If there was uncertainty about whether a manuscript was relevant or not, it was decided to include it for full-text review.

### Exclusion criteria

The following search results were excluded from this systematic review:

- Review papers, including systematic reviews, meta-analyses, and narrative reviews
- Single patient case reports (case series or case studies with more than one patient were included)
- Animal studies/experimental studies
- Dissertation abstracts
- Book chapters/books
- Studies focusing on children, without a predominantly adult population
- Intervention studies addressing cognitive dysfunction (amelioration or prevention studies)

### Screening process

Manuscript titles were initially screened by one author (MAK, a medically qualified specialist in neurosurgery) to identify potentially relevant articles. Then, abstracts of screened studies were screened independently by MAK and another medically qualified researcher (BH) to identify relevant studies. Where ambiguity regarding eligibility persisted, the full article was reviewed and disagreements were resolved by consensus.

### Data extraction process

Data from studies meeting our inclusion criteria were extracted using a standardized data extraction proforma and critically appraised. The relevant information extracted from the manuscripts included: study setting; study population, participant demographics and baseline characteristics; details of intervention and control conditions, where applicable; study methodology; recruitment and study completion rates; outcomes and times of measurement.

Due to the wide variations in study design and outcome measures, it was not possible to perform a meta-analysis.

## Results

### Selected articles

**Figure 1:**
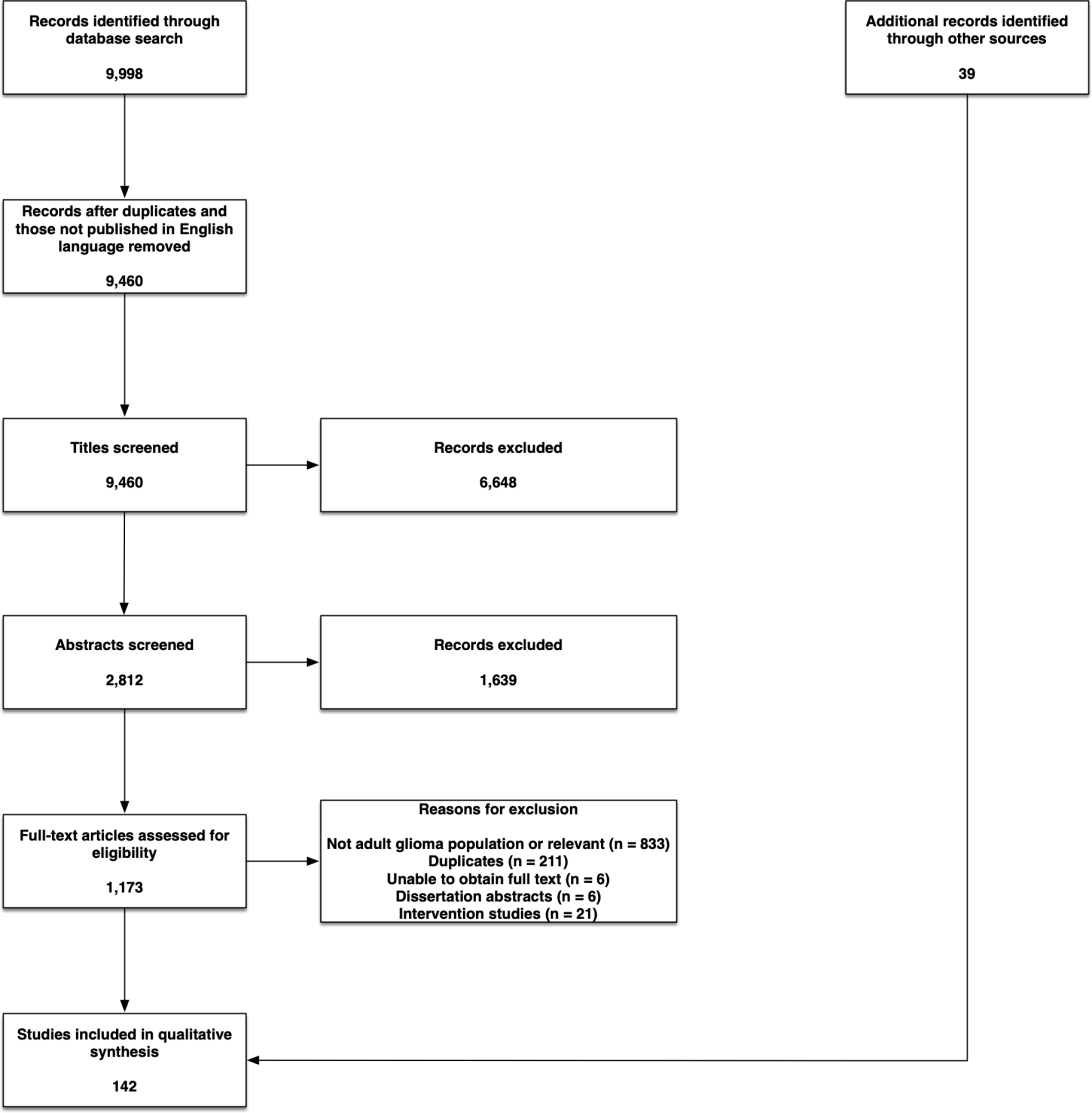
Study flow chart.

The search strategy identified 9,998 articles (**Figure 1**). After excluding duplicates and articles not published in the English language, 9,460 articles remained for title screening; of these, 2,812 articles were selected for abstract review. There was agreement in the decision for inclusion/exclusion among 2,518 (89.54%) of the 2,812 articles identified through the search strategy at the abstract screening stage, with a resulting kappa statistic of agreement of 0.781 (95% confidence interval = 0.757–0.804), which can be defined as ‘substantial agreement’ (22). There was disagreement on inclusion/exclusion of 294 of the studies. After a consensus meeting, 228 of these 294 studies were included for full-text review. A total of 1,173 articles were identified by the search strategy, and an additional 39 articles were identified from other sources, for full-text review. Of these, 142 manuscripts met our inclusion/exclusion criteria. A summary of the included studies is provided in **Supplementary Table 3**, and a flow chart of the selection process is shown in **Figure 1**.

### Characteristics of included studies and study settings

An overview of the identified studies is shown in **Table 1**.

**Table 1:** Overview of the studies included in this systematic review.

Study characteristics	N
	142
**Study design**	
- Randomised controlled trial, including secondary analyses of data collected as part of a randomised controlled trial	7
- Prospective non-randomised study	85
- Retrospective study	28
- Mixed prospective and retrospective	1
- Cross-sectional study	1
- Case series	2
- Not specified	18
**Location of study authors**	
- North America	33
- Europe*	72
- Asia	14
- UK	5
- More than one continent	15
- Australia	1
- Middle East	2
**Decade study published**	
- 2020 onwards	38
- 2010-2019	66
- 2000-2009	30
- 1990-1999	8

*Turkey classified as Europe.

The majority of studies were authored by researchers either solely based in Europe (n=72, 50.7%) or North America (n=33; 23.2%), and 14 (10.6%) by authors from more than one continent. Most studies were published since the year 2000 (between 2000 and 2009: n=30, 21.1%; between 2010 and 2019: n=66, 46.5%; 2020 onwards: n=38, 26.8%).

It is important to note that there was a paucity of studies identified evaluating the role of environmental and demographic factors on cognitive outcomes in patients with glioma; the overwhelming majority of studies focused on clinical factors.

### Study quality and level of evidence

The majority of included studies were prospective non-randomized studies (n=85, 59.9%), There were also 7 (4.9%) randomized controlled trials (RCT) or studies presenting secondary analyses of RCT data identified. The study design was not clear or specified in 18 (12.7%) studies.

### Data synthesis

#### Tumour-related factors

The identified studies found wide variability in the reported effects of tumor-related factors on cognitive outcomes in patients with gliomas. Anatomical location of the tumor has been shown to be an important influence on cognitive outcomes in some studies (23–35), but not in others (3, 36–40); although most of the studies providing evidence for and against a role of anatomical location were prospective cohort studies (some study designs in both groups were not specified), the studies suggesting no role of anatomical location were fewer and tended to have smaller numbers of participants, and on balance anatomical location is most likely to be relevant overall for cognitive function. Location of the tumor has been shown to influence the likelihood of developing deficits in some but not all cognitive domains (41), and in another study was found to predict spontaneous speech deficits and naming scores in HGG but not LGG patients (42); the authors of the latter study hypothesize that this implies that large functional reorganization occurs in LGG, and highlights the importance of glioma grade in macrostructural plasticity mechanisms that modulate brain-behavior relationships.

In addition to the specific lobe affected, tumor laterality has been shown to influence cognitive outcomes. Many studies have shown that tumors in the right hemisphere (which is the non-dominant hemisphere in the majority of the population, including most left-hand-dominant individuals) or right-hemisphere interventions are associated with a lower risk of cognitive impairment (15, 33, 43–49), sometimes irrespective of the exact location of the tumor within the hemisphere (50). However, some studies have shown that cognitive effects of tumor laterality depend on the cognitive modality under assessment (51–55). Some studies have shown tumor laterality to not influence cognitive outcomes, or that left-hemisphere interventions consistently induce cognitive decline (36, 56, 57). Indeed, some evidence indicates right-sided pathology increases vulnerability to cognitive impairment. In one study of 59 patients with high and low-grade gliomas that underwent neuropsychological assessment before and one year after surgery, the brain regions most vulnerable to cognitive decline after surgery were found in the right cerebral hemisphere (58); in this study, the most commonly affected cognitive domains were attention and information processing speed. One prospective study of LGG patients found that, although those with left hemisphere tumors were more impaired in verbal measures at baseline than those with right hemisphere tumors, they demonstrated greater improvement in verbal memory over the five-year follow-up period of the study (44). One study of 66 patients with gliomas who underwent awake craniotomies found visuospatial cognitive deficits persisted in 14.3% of patients with right-sided lesions, and recovered fully in all patients with left-sided lesions (41), which is unsurprising given the established association between unilateral spatial neglect and right-hemisphere lesions (59). However, overall, tumor laterality appears to be important in cognitive outcomes.

Size of the tumor as evaluated on imaging has been shown in some studies to influence cognitive outcomes (4, 54, 60), and particularly for tumors affecting the frontal, temporal, and parietal lobes (26, 43), but some studies have found no effect of tumor volume on neurocognitive functioning (3, 15, 36, 39, 42, 47, 52, 61). The role of tumor size may depend on the underlying genetic make-up of the tumor: one study found an inverse relationship between neurocognitive function and lesion volume in patients with IDH1 wild type but not mutant tumors (7). On balance, the evidence is overall more against a role of tumor volume on cognition, with most studies concluding this being prospective in design and including a randomized trial (n=187); two of the three studies supporting a tumor volume were retrospective in design (54, 60), although one of these had a large study population (n=780) (54). Invasiveness of a tumor determined using MRI has also been shown to be associated with neurocognitive functioning (34).

The role of tumor grade in cognitive outcomes is unclear, with evidence for (4, 32, 42, 54, 62–67) and against (15, 40, 50, 60, 68, 69) a role. Both groups contained largely prospective studies and no clear superiority in evidence one way or another although there is more evidence for a role of tumor grade in cognitive outcomes than against. One study found differences in pre-operative neurocognitive function according to glioma grade (with higher grade tumors associated with worse cognition) even when controlling for MRI-determined tumor volumes (63). There is some evidence that tumor grade is associated with cognitive outcomes for some but not all cognitive domains (41). HGG has been shown to be associated with lower language scores and more language and cingulo-opercular/fronto-parietal network disruptions prior to treatment compared to LGG (65). In another study, HGG was associated with significantly worse language impairment than LGG even when controlling for variables such age, sex, education, and tumor volume (42). In a prospective study of 16 patients (half with high-grade brain tumors and half with low-grade brain tumors), all but one of whom received radiotherapy, compared to a ‘control’ group of eight patients with ‘non-malignant’ brain tumors (meningiomas) who did not receive radiotherapy, there was a differential pattern of cognitive performance observed between the low- and high-grade brain tumor groups following radiotherapy; the low-grade tumor group’s performance was superior across all five main neuropsychological measures, and their pattern of improvement was similar to that of the non-malignant brain tumor group that had not received radiotherapy (62). Other studies have found patients with HGG more likely to improve in relation to neurocognitive functioning following surgery compared to those with LGG (4, 64); this may be because patients with HGG have had little time for functional brain reorganization, and thus their recovery may be facilitated by removal of the physical tumor, whereas in LGG functional reorganization may have already taken place with some functions being subserved by other brain regions or networks. This is supported by the results of a study of 119 patients with malignant gliomas that compared neurocognitive performance according to IDH1 mutation status (7); IDH1 wild type tumors, which generally more aggressive and grow faster, are associated with reduced neurocognitive function compared to those with IDH1 mutant tumors.

Tumor biology, including associated molecular and histopathological profiles, has been shown to influence cognitive outcomes in patients with glioma (7, 52, 54, 70), but not in others (41, 60); all of these studies were retrospective, although the evidence supporting a role for tumor biology in cognitive outcomes included larger populations [n = 168 (54) and n = 197 (70)] albeit from the same research group. Such discrepancies may result from differences in the specific tumor biological characteristics studied as well as the methodological differences between studies. Molecular characteristics and their influence on cognitive outcomes have also been an area of study. One such study evaluated the relationship between cognitive performance (executive function, memory, and psychomotor speed) and the intratumoral expression levels of molecular markers in patients with diffuse glioma prior to treatment; after correction of tumor volume and location, significant associations were identified between expression levels of CD3 and IDH-1 and psychomotor speed, as well as between IDH-1, ATRX, NLGN3, BDNF, CK2Beta, EAAT1, GAT-3, SRF, and memory performance, and between IDH-1, P-STAT5b, NLGN3, CK2Beta, and executive functioning (70). There were also independent associations identified between P-STAT5b, CD163, CD3 and Semaphorin-3A after correcting for histopathological grade. The authors concluded that variations in glioma biology can influence cognitive function through mechanisms that include disturbed neuronal communication.

#### Patient-specific factors

There are a range of patient-specific factors that have been shown to influence cognitive performance in patients with brain tumors, including basic demographics such as age (28, 71–73), although some have found age to not influence cognitive outcomes (38, 61, 74); all of these studies were prospective and, although the studies supporting a role of age were generally larger, one study providing evidence against a role of age was a randomized controlled trial with a good sample size (n=187) (61). There is wide variability in language localization between individuals (75), which means that a tumor located in a specific anatomical location may have different effects across the population. This may explain why not all patients with tumors in areas of the brain deemed ‘critical’ for language have cognitive disturbance (76). Other factors that influence study results have been identified; for example, in a study of 20 adult LGG patients who underwent tests of writing fluency and oral lexical retrieval, typing speed accounted for some of the differences observed between the LGG patients preoperatively and a reference group of 31 individuals without neurological disease (23). Poor performance in timed tasks among patients with HGG has been found to be largely attributable to the presence of visual and motor deficits (49). Low thyroid hormone levels (hypothyroidism) are known to affect cognition in the general population, and a prospective analysis of 230 patients with a range of primary brain tumors, including meningioma, LGG, HGG, pituitary adenoma, and acoustic neuroma, found low levels of the thyroid hormone tri-iodothyronine to be common (74%) and associated with lower Mini-Mental State Examination (MMSE) scores (77). A patient’s performance (functional) status may also be a risk factor for postoperative cognitive dysfunction (32). Female sex has been shown to be associated with better language performance immediately after surgery, as well as a faster recovery, but at one year after surgery scores were comparable (78).

Baseline cognitive status (that is, an individual’s cognitive ability at the time of glioma diagnosis or prior to commencing treatment) is also important in predicting outcomes of treatment for low-grade tumors (71, 79, 80), and is likely to at least in part reflect earlier influences such as education, socioeconomic status, and cognitive reserve (discussed later). In a prospective study of 27 patients with a range of low-grade brain tumors, including pituitary tumors, meningiomas, and LGGs, diffusion tensor imaging (DTI) metrics were used along with a range of cognitive tests to correlate with cognitive performance assessed prior to starting radiotherapy as well as six and 18 months later (79); in multivariate analysis, the only clinical variable that predicted changes in verbal memory was baseline neurocognitive score, with most improvement in verbal memory score observed in those with worse baseline cognition, and no clinical variable was able to predict verbal fluency changes. In another study including 24 patients with temporal lobe epilepsy associated with low-grade brain tumors (gliomas and other tumors) and 36 healthy controls, preoperative memory test scores were the most important contributor to postoperative memory test scores, as shown by the strongest correlation of all studied variables, after which other relevant factors included laterality of the surgery, age, and education (71). Another prospective study of 9 HGG and 9 LGG patients found that patients with preoperative neurocognitive dysfunction tended to have persistent cognitive deficits, and that visuospatial dysfunction often persisted until the chronic phase of the disease, which may reflect damage to the white matter bundles superior longitudinal fasciculus I and II (80).

Changes to the white matter tracts of the brain, as determined using fractional anisotropy, appear to correlate with cognitive test results (81) and lateralization of the arcuate fasciculus appears to predict language deficits in patients with brain tumors (82). Increased radial diffusion on DTI in the parahippocampal cingulum white matter at the end of radiotherapy has been shown to significantly predict decline in verbal fluency 18 months following radiotherapy in patients with a range of low-grade brain tumors (79). Differences in the functional networks of a patient’s brain also appear to be important in cognitive outcomes in LGG patients; for example, differences in functional connectivity between key regions of the frontoparietal network are associated with cognitive performance in patients with gliomas, and are associated with cognitive outcomes following surgery (83). In patients with unilateral temporal glioma, intrinsic regional activity in the contralesional hippocampus and parahippocampal regions, determined using resting state functional MRI, has been shown to negatively correlate with visuospatial scores, but not with other cognitive measures (84). In a magnetoencephalography (MEG) study of glioma patients with epilepsy, network characteristics correlated with clinical presentation in relation to seizure frequency in LGG patients, and with poorer cognitive performance in both LGG and HGG patients; more specifically, decreased synchronisability and decreased global integration in the theta band were associated with the occurrence of seizures and cognitive decline (85).

#### Surgery and extent of tumour resection

Studies have been conflicting in relation to the findings of the effect of surgery on cognition. This is probably at least in part due to the complex interaction between surgery and other relevant variables, particularly tumor location, in modulating cognitive outcomes; from a statistical perspective, the surgical procedure (including the surgical approaches and goals) are likely to depend on the anatomical and other clinical properties of the tumor, and thus surgery and other variables are not independent but are instead confounded predictive factors for cognitive function in patients with glioma. It is also likely to be due to the heterogeneity in cognitive tests used in the different studies. Some studies have found a deterioration in cognition following surgery often with partial or full recovery (or even improvement) in the ensuing months (3, 24, 33, 41, 47, 48, 64, 71, 78, 86–100), others an improvement in cognition (37, 52, 101–103), and others no effect of surgery at all (28, 37, 68, 104–106) including in both languages of bilingual speakers following awake craniotomy with intraoperative mapping (107). One study found selective deterioration in specific cognitive domains at 3 and 12 months following surgery but no overall cognitive impairment at the group level (108). Second surgery for recurrent HGG and LGG has been shown to be possible without significant cognitive damage in the months after surgery (109). Other studies have found a mixed picture of some patients experiencing cognitive improvement and others in the same study who experience cognitive decline or no change (39, 58, 60, 76, 110–113), or improvements in some cognitive domains and deterioration (2) or no change (46) in others; such a pattern could indicate ‘noise’ within the cognitive data. The cognitive domain being tested is important, as shown by one study where a decline in most cognitive domains was observed 5 days after surgery compared to pre-operatively, but only memory remained impaired 1 month after surgery (91). Overall, the data suggest that surgery influences cognition negatively initially followed by a recovery over several months in most cases.

Understanding the anatomical substrate driving cognitive changes following brain tumor resection has been an area of particular interest. Brain tumors, as well as the associated treatments, have been shown to influence brain networks fundamental for memory (39). An inverse relationship between neurocognitive function and changes in network properties assessed through resting-state fMRI has been shown in a small study of patients with left perisylvian gliomas who underwent awake tumor resection (94). Language function reorganization following surgery has been observed using functional MRI and magnetoencephalography (112), and magnetoencephalography has been used to identify brain regions of high functional connectivity within around HGGs and LGGs, which are associated with early postoperative decline in some cognitive functions that are transient (99).

Surgery may be associated with the development of complications, such as stroke, that may contribute to cognitive outcomes; for example, a study of LGG patients including 33 who underwent computerized cognitive testing pre- and 3 months postoperatively found that neurocognitive functions between those that did and did not develop an infarct were generally stable, but those that developed a stroke experienced a decline in verbal rhyming ability (114). In a series of patients with giant insular gliomas, ischaemic insults in eloquent brain regions were shown to be the leading factor associated with long-term neurological and neuropsychological morbidity (93). However, another study suggested the presence of small infarcts was only associated with a slight decrease in semantic fluency scores four months following surgery in patients with gliomas (115).

One would expect that the specific surgical approach chosen to remove a brain tumor may be an important determinant of cognitive outcomes; for example, when attempting to remove a tumor located deep in the brain, one may expect worse cognitive outcomes if traversing cognitively eloquent structures, although some data indicate this to not appear to be the case (104). Similarly, the extent of tumor resection achieved by surgery has been shown in multiple studies to not influence cognitive outcomes (3, 4, 36, 38, 47, 49, 50, 52, 60), although one study interestingly found higher extent of resection to be positively associated with cognitive outcomes (116). One study comparing total to supratotal (i.e., removal of brain tissue beyond the tumor borders detectable on imaging) resection in patients with radiologically presumed LGGs found that memory, language, and fluid intelligence were not influenced by extent of resection, but praxis was better in the total resection group immediately after surgery (although this difference reversed after 3 months); furthermore, there was a better recovery of executive functions in the supratotal resection group (78).

Generally, studies have found varying effects of surgery on different cognitive domains; for example, one prospective study of 14 patients who underwent surgery for frontal or precentral gliomas found improved verbal memory following surgery, but unchanged or worsening visuo-spatial performance, and slightly worsened alertness (98). This highlights the importance of a comprehensive battery of neuropsychological testing to ensure that all changes in cognitive function are detected.

There has been great interest in identifying pre-operative predictors of cognitive outcomes in patients with low-grade brain tumors. To this end, in a small prospective study of 10 patients with WHO grade II gliomas who underwent MEG prior to and a mean of 16 weeks (range, 11-25 weeks) after resective surgery, increased alpha band resting-state network functional connectivity on MEG was found to correlate with improved cognitive outcome post-operatively, supporting the notion that cognitive changes following surgery correspond to changes in connectivity in resting-state networks (117).

For gliomas that involve eloquent parts of the brain, awake craniotomies are performed to enable the surgeon to monitor neurological well-being during surgery and prevent complications. Awake surgery with intraoperative mapping has been shown to facilitate preservation of visuospatial cognition and spatial working memory in patients with right frontal gliomas (118), and extensive intraoperative mapping for cognitive, visual and haptic functions in patients with giant insular gliomas has been shown to decrease long-term neurological, neuropsychological, and quality of life morbidity as well as increasing the extent of resection (93). In comparison to tumors in the same anatomical regions operated on under general anesthesia without brain mapping, tumors operated on through awake craniotomy with intraoperative mapping are associated with better neuropsychological outcomes six months following surgery, particularly those located in the parietal and insular lobes (119). One study compared rates of permanent surgery-related language deterioration in patients who underwent an awake craniotomy in the presence of a neuropsychologist to those without the presence of a neuropsychologist, and found no significant difference (120). Another study of intraoperative stimulation mapping during awake craniotomy in LGG patients found its use to be associated with slightly worse cognitive performance after surgery (57), despite a similar extent of resection. Electrophysiological mapping can also be performed in asleep craniotomies to identify the inferior frontal gyrus (Broca’s area) with the aim of preserving speech functions post-operatively (121).

#### Chemotherapy

It has been postulated that chemotherapy could have a deleterious effect on cognition through the development of acute and chronic encephalopathy (18). However, chemotherapy has been shown to have a positive effect on cognitive outcomes in some studies (122), and no effect in others (3, 68, 123, 124). One analysis of RCT data from 251 patients with LGG evaluating the effect of adding chemotherapy to radiotherapy treatment found no significant increase in cognitive decline with the addition of chemotherapy during the five-year follow-up, but only evaluated cognition using the MMSE (123).

Studies evaluating the effects of chemotherapy on cognitive outcomes in patients with LGG are limited by small numbers and the grouping together of patients who have had multiple types of chemotherapy (each of which have their own mechanism of action) and indeed other treatments (including radiotherapy). For example, a small prospective study of 25 patients with LGG found that cognitive outcomes were influenced by whether or not oncological treatment, i.e., chemotherapy and/or radiotherapy, was administered (125); however, the treatment group (n = 16) comprised of patients receiving radiotherapy alone (n=5), radiotherapy and carboplatin chemotherapy (n=1), or chemotherapy alone (n=3). Larger studies with more homogenous treatment populations are needed to fully elucidate the effects of specific treatments on cognition in patients with LGG.

#### Radiotherapy

Radiotherapy appears to affect the cerebral vasculature and the white matter tracts, resulting in demyelination, vessel wall thickening, and coagulative necrosis (18). These changes have been associated with cognitive impairment that may be protracted for several years after the completion of radiotherapy treatment (126, 127), and cognitive decline can be noted as early as 18 months after radiotherapy treatment for low-grade brain tumors (79). There is a clinical spectrum of cognitive deficits in patients following radiotherapy that ranges from mild to moderate to dementia, and such deficits occur in at least 12% of patients treated with cranial radiotherapy (128).

Radiotherapy has been widely shown to negatively influence cognitive outcomes in patients with brain tumors (32, 39, 129–138), but not all studies have found such a relationship (3, 28, 68, 139–141), and in some cases mixed results (61, 142–144) and improvements in cognition following radiotherapy (122) have been noted. These conflicting findings may be due to differences in study design, outcome measures, and/or timing of assessments; for example, two large randomized studies that found most patients maintain stable neurocognitive status after radiotherapy relied on MMSE scores alone in the assessment of cognitive function (61, 141), and other studies have also relied solely on general screening tools such as the MMSE to evaluate for cognitive impairment (145). Furthermore, although a different study found non-significantly improved neurocognitive test scores at a second evaluation relative to the baseline (pre-radiotherapy) evaluation (38), this may be due to practice effects associated with the test.

The additive and differential effects of radiotherapy and chemotherapy on cognition have been studied with great interest. For example, one three-arm RCT of 36 adult patients with newly diagnosed WHO grade III oligodendroglioma compared radiotherapy alone to chemoradiotherapy and chemotherapy alone; the primary outcome was overall survival, but the study found no difference in neurocognitive decline from baseline to 3 months between the three arms (146). Another follow-on study of a different RCT comparing outcomes following radiotherapy to chemotherapy alone as a primary oncological treatment in 99 patients with LGG found no significant difference in cognitive outcomes at 12 months between the two treatment arms (122), although longer-term follow-up would likely be necessary given the timespan over which radiotherapy can affect cognitive function.

Several studies have found neuroimaging correlates of impaired cognitive function associated with radiotherapy, including cortical atrophy and white matter abnormalities (131, 132, 135, 137). Although it is widely felt that cognitive outcomes are affected at specific radiation fraction doses, for example above 2 Gy (133), it has been shown that doses less than this can result in declined attentional functioning (132). In another study, however, no effect of gross, clinical, or planned radiotherapy target volumes on memory functions were found in patients with LGG; this same study also found no deleterious effect of radiotherapy on memory function compared to temozolomide chemotherapy treatment (122).

There is evidence against late cognitive and radiographic changes related to radiotherapy. A prospective long-term study of 26 patients with low-grade, supratentorial brain tumors (including gliomas as well as pituitary tumors, pineal tumors, and non-invasive meningiomas) with annual follow up for six years found that, although half of the patients showed evidence of cognitive decline and treatment-related T2-weighted MRI hyperintensities, there was no evidence of general cognitive decline or progression in white matter changes associated with radiotherapy after 3 years of follow-up (129).

The effects of radiotherapy on cognitive outcomes are likely to relate to the specific anatomical structures that have been irradiated and their laterality (136, 138). A retrospective analysis of 57 patients with a range of brain tumors, including 35 benign or low-grade tumors, aimed to identify neuroanatomical targets of radiation-induced cognitive decline by correlating performance in neurocognitive assessments with dose volume histogram analyses of specific brain regions of interest (136). The authors found that the corpus callosum, left frontal white matter, right temporal lobe, bilateral hippocampi, subventricular zone, and cerebellum were able to predict global cognitive outcomes at radiation doses of <60 Gy; regions that did not predict global cognitive outcomes at any dose include total brain volume, frontal pole, anterior cingulate, right frontal white matter, and the right precentral gyrus. This was a retrospective study and further prospective data are required to validate these findings.

#### Genetics

The role of genetics and its interaction with brain tumors in modulating cognitive outcomes has been studied previously. In a study of 128 patients with LGG and HGG, polymorphisms in COMT, BDNF, and DRD2 genes were found to be associated with cognitive performance; more specifically, patients with high-performing alleles had better scores in the Repeatable Battery for the Assessment of Neuropsychological Status and Stroop tests, but not the Trail Making Test (TMT) (147).

A study focusing on 233 patients with HGG and LGG found polymorphisms in inflammation, DNA repair, and metabolism pathways were associated with cognitive function prior to surgical resection, with those harboring at-risk variant alleles at greater risk of cognitive dysfunction (148). Another study of 150 patients with a range of tumor types including HGG, LGG, and primary CNS lymphoma found strong associations between attention, executive functions, memory and 33 single-nucleotide polymorphisms in genes involved in late-onset Alzheimer’s disease, inflammation, cholesterol transport, dopamine regulation, myelin repair, DNA repair, cell cycle regulation, and response to oxidative stress (149). Of note, the genetic findings were not associated with white matter abnormalities on brain MRI scans. The presence of mutations in IDH genes have been shown to be associated with better cognitive outcomes in patients with HGGs (7), which is of relevance to patients with LGG given that the majority of LGG patients have such a mutation. However, equivalent studies are yet to be performed in patients with LGG.

In a study of 40 LGG patients of whom 36 had undergone testing for the APOE ϵ-4 allele, APOE ϵ-4 allele carriers (n=9) had non-significantly lower scores in verbal memory than non-carriers (n=27), but there were no differences in other domains (131). In a subsequent study by the same authors of 20 patients with high- and low-grade gliomas stratified according to whether or not they harbored the APOE ϵ-4 allele, a battery of cognitive tests were administered at two time points: first, a mean of 4 years after completing treatment, and second a mean 5.21 years later (126). Mean age was similar in the APOE ϵ-4-positive (51 years, standard deviation [SD] 9.1 years) and -negative (50 years, SD 9.9 years) patients. Imaging for detection of β-amyloid deposition (using ^18^F-florbetaben positron emission tomography [FBB PET]) was also performed. The study found a significant decline in attention and an almost-significant decline in verbal learning. There were significant differences over time in attention/working memory according to APOE status, with a decline noted in the APOE ϵ-4 carriers. There were no significant differences in the FBB PET findings between APOE ϵ-4 carriers and non-ϵ-4 carriers. The results of this study indicate that glioma patients may experience worsening attention and executive functions several years following treatment, and that the APOE ϵ-4 allele may modulate cognitive decline independently from increased β-amyloid deposition.

Not all studies have found genetics to influence cognitive outcomes. One study of 505 patients with HGG, LGG, or meningiomas found that carriers of the APOE ϵ-4 allele (mean age 54.9 years, SD 13.9 years) did not have an increased risk of pre-treatment cognitive dysfunction or cognitive decline within one year of debulking surgery relative to non-carriers (mean age 55.3 years, SD 13.1 years) (150). However, this does not exclude the presence of potential late treatment effects.

Although APOE status is associated with cognitive decline in older age in the general population (151), the similar ages between APOE ϵ-4 carriers and non-carriers in the above studies argue against this being a driver of the observed findings. It is most likely that genetics influence the effects of gliomas on cognition both directly (for example, through modulating the plasticity of reorganization) and indirectly (for example, through cognitive and/or brain reserve, discussed later).

#### Seizures and anti-epileptic medications

Epilepsy is common in patients with gliomas, affecting 86% of patients with LGG according to one study (152). Anti-epileptic drugs are known to be associated with cognitive decline (36, 46, 49, 131, 133, 153, 154), particularly the older anti-epileptic medications (155), although most of the studies performed to date have suffered from poor methodological quality (including non-randomization of anti-epileptic drug use) and heterogenous reporting of outcomes (155, 156). Some data suggest no influence of seizures or anti-epileptic medications on cognitive outcomes in patients with brain tumors (61, 63, 157), but these studies include limitations of small sample size (157) and lack of comprehensive cognitive assessment (use of MMSE alone) (61). Overall, most evidence supports a role for anti-epileptic drug use (and, to a lesser extent, seizure burden) in adverse cognitive outcomes.

The number of antiepileptic medications prescribed to a patient has been shown to influence short-term memory in patients with LGG 40 months after surgery, despite the lack of a relationship with other factors such as tumor laterality, lobe affected, tumor volume, or extent of resection of the tumor (36). In another prospective study of 195 LGG patients, the use of antiepileptic medication was significantly associated with disability in attentional and executive function and epilepsy burden was found to affect cognitive function more than radiotherapy (133). As shown by a more detailed analysis of a large portion of this cohort, the intensity of the epilepsy treatment is a more important contributor to cognitive outcomes than the severity of the patient’s epilepsy, as patients that received anti-epileptic drugs had more cognitive impairment, even in the absence of seizures, supporting the notion that it is the anti-epileptic medications that primarily influence cognitive function (152).

Importantly, anti-epileptic drugs may also interact with chemotherapy treatments used in patients with brain tumours (158, 159) and this may affect survival as well as cognitive outcomes, although this remains to be proven.

#### Steroids

Corticosteroids are regularly used in the treatment of brain tumors to reduce swelling from cerebral oedema, which is known to affect cognitive outcomes (4, 43). However, corticosteroids are also known to result in cognitive, psychiatric, and behavioral dysfunction, and there are data to indicate that their effects on attention, concentration, and memory are a result of neurotoxicity to the hippocampal and prefrontal areas (160). Most data on the adverse cognitive effects of corticosteroid therapy comes from patients with systemic conditions treated with steroids as opposed to brain tumors. A study of 18 patients with a range of brain lesions (mainly tumors) found language abilities improved in 4 of the 6 patients that showed language impairments 1–5 days postoperatively, when tested again at 6–9 days, following a wean of dexamethasone in the days following surgery (112). A study of 72 patients with HGG and LGG evaluated using cognitive testing prior to any surgery or oncological treatments found no effect of steroid use on cognitive performance (63). There is evidence from patients with HGG, however, that corticosteroid use is associated with improved recognition memory, which is likely to be due to resolution of cerebral oedema (49). The most robust evidence on corticosteroid use comes from an analysis of data collected from an RCT that effect of corticosteroid use on neurocognitive function in 321 patients with recurrent glioblastoma, using data from the European Organization for Research and Treatment of Cancer trial 26101; it found that patients on corticosteroids had worse neurocognitive function in all tested domains (memory, expressive language, visual-motor scanning speed, executive functioning) compared to those not on corticosteroids with significant inverse correlations between corticosteroid use and Hopkins Verbal Learning Test - Revised Free Recall and Delayed Recall scores (161). There is limited evidence of the effect of corticosteroids on cognition in patients with LGG, which is likely to be due at least in part to the fact that LGGs tend to cause cerebral oedema less frequently than many other tumor types. The results of the above studies must also be interpreted in light of the fact that corticosteroid treatment was not randomly allocated to participants in any of the studies, opening up the results to bias as steroid treatment is likely confounded by specific characteristics of the patient and their tumor.

#### Fatigue/sleep disturbance

Fatigue is a common symptom in patients with brain tumors. For example, in a prospective study of 12 children with low-grade tectal tumors, 50% had fatigue during follow-up and this was the commonest symptom reported (162). Fatigue is also a common side-effect of a number of anti-epileptic medications (163). There are no studies that have reliably connected fatigue with cognitive outcomes in patients with gliomas, but it is highly plausible that fatigue is likely to affect cognition and performance in cognitive testing. Further studies are needed in this regard.

#### Mood disorders

Mood disorders are common in patients with brain tumors (164), and particularly in gliomas relative to other types of brain tumors (165). Some of the treatments used for brain tumors are also associated with depression, and depression is associated with negative outcomes such as shorter survival in patients with brain tumors (166, 167). However, whether treatment of depression improves cognitive and other outcomes in patients with brain tumors remains to be elucidated. There is some evidence to suggest that a negative correlation exists between cognitive performance and psychological distress, in particular depression (28, 168). This suggests that patients with higher cognitive performance are less likely to be psychologically distressed, and counteracts the argument that low psychological distress is attributable to a lack of insight associated with cognitive impairment.

There is some evidence for a role of mood state in cognitive outcomes in brain tumor patients (8, 26, 64, 74, 169). In one study of 141 brain tumor patients who were divided into groups depending on the tumor location, negative mood changes were identified after the resection of brain tumors involving heteromodal cortices located either prefrontal or temporoparietal, whereas positive mood changes were identified after lateral frontal resections (26). Postoperatively reported levels of fatigue, irritability/anger, and anxiety/depression were positively correlated with the extent of cognitive impairment, significantly so for paired associate learning, Similarities, Block Design, Picture Completion, and Visual Span tasks. However, when analyzing by lesion group in multivariate analyses, basic cognitive and attentional performance did not seem to contribute significantly to the reported mood levels. In another study, depressive symptoms were correlated strongly with many aspects of health-related quality of life but not neurocognitive functioning (8). However, another study in patients with HGG and LGG found an association between both mood disorders and low quality of life and cognitive deficits from pre-operatively to one year after surgery (169). Another study of patients with low- and high-grade brain tumors including objective and self-reported cognitive outcomes found those reporting worse cognitive impairment had worse depressive symptoms (74).

#### Cognitive and brain reserve

Very few studies have evaluated the role of cognitive and brain reserve in patients with brain tumors. Brain reserve can be defined in terms of individual differences in the brain itself that allow some individuals to cope better than others with a given pathology; the differences may be quantifiable, such as brain volume or the number of neurons or synapses (170). Cognitive reserve, on the other hand, has been defined in terms of individual differences in the processing of tasks that allow some to cope with brain pathology better than others (170); this type of reserve has been further sub-classified into neural reserve and neural compensation. Neural reserve refers to inter-individual variability in the brain networks/cognitive paradigms that underlie performance in tasks in the healthy brain. This variability could be in efficiency, capacity, or flexibility, and the implication is that those with more efficient, capacious, and/or flexible brains are more able to cope with the disruption imposed by brain pathology. Neural compensation instead refers to inter-individual variability in the ability to compensate for the disruption of standard processing networks, caused by brain pathology, using brain structures or networks not normally used by individuals with ‘intact’ brains. This compensation may help maintain or even improve performance (170). Most studies have found that the most commonly used proxy of cognitive reserve in the literature (years of formal education) does not significantly predict outcomes (28, 39, 72, 171), but there are exceptions (71) including a study that found it to influence cognitive outcomes in HGG but not LGG (42). One analysis of RCT data that evaluated patient factors that predicted responsiveness to a cognitive rehabilitation program in 64 patients (including 54 with LGG), and found younger age and higher education were predictors of benefit from the program - proxy measures of brain reserve and cognitive reserve, respectively (172). Age has been shown to influence cognitive outcomes in some (28, 71–73) but not all (38, 61) studies.

A recent study explored whether the concept of cognitive reserve can be applied to cognitive functions in 100 patients with brain tumors (HGG, LGG, and meningioma); different proxies for cognitive reserve (education level, premorbid IQ, current IQ, working and leisure activity) were investigated in terms of their role in protecting language function against the effects of brain tumors and surgery, when considering interactions with demographic, anatomical, and clinical/biological variables (171). The study found premorbid IQ (evaluated using the Italian equivalent of the National Adult Reading Test [NART]) was the best predictor of pre-operative language integrity, over and above all clinical variables evaluated. Furthermore, patients with worse pre-operative language integrity and low-to-moderately aggressive tumors showed a mitigating effect of current IQ over the consequences of surgery. The authors concluded that different cognitive reserve proxies play a role in moderating cognitive decline following brain tumors and surgery. The results of this study also indicated that cognitive reserve influences language outcomes in patients with brain tumors over and above the location of the lesion. This is supported by a study that evaluated whether cognitive reserve predicts cognitive performance of 91 patients with non-frontal lesions (high-grade tumors, low-grade tumors, meningiomas, or stroke) compared to 166 with frontal lesions and 136 healthy controls (73). In this study, NART IQ was found to predict executive, intelligence, and naming performance. Age was also found to significantly predict performance on executive and processing speed tests. Being part of the frontal group predicted executive and naming performance, while being part of the non-frontal group predicted intelligence. The authors concluded that age, lesion group, and literacy attainment have independent roles in predicting cognitive performance following stroke or brain tumour; however, the relationship between NART IQ and cognitive performance following focal brain damage does not differ in relation to frontal and non-frontally located lesions, implying that environmental factors shape resilience to cognitive decline in both of these groups (73).

One study aimed to evaluate the independent effects of two cognitive reserve proxies, education and NART IQ, on a range of cognitive domains in 86 patients with focal frontal lesions compared to 142 healthy controls (72). Education and NART IQ were highly correlated with each other (r=0.48, p<0.001). However, linear regression models testing for the effects of education and NART IQ on multiple cognitive tests, with chronicity, age, and frontal lesion severity as covariates, were examined for multicollinearity using the variance inflation factor; this was below 2 in all instances, indicating a lack of high intercorrelations among the predictor variables. Only NART IQ predicted executive and naming performance. Neither education nor NART IQ predicted performance on fluid intelligence, processing speed, verbal short-term memory, or perceptual abilities. Education and NART IQ did not modify the effect of lesion severity on cognitive impairment. Age significantly predicted performance on executive tests and most of the other cognitive measures except verbal short-term memory and naming. Age was the only predictor for fluid intelligence, suggesting that age plays a role in executive performance over and above the contribution of cognitive reserve proxies with focal frontal lesions. The authors concluded that the studied cognitive reserve proxies do not appear to modify the relationship between cognitive impairment and frontal lesions.

Overall, it appears that NART-IQ is a good proxy measure of cognitive reserve and that cognitive reserve protects cognitive functions as it mitigates the effect of lesion severity. The data from these studies also support the notion that lesion location is not as important for language and other cognitive outcomes as one may expect.

#### Treatment timing and effects on cognitive outcome

Several retrospective studies have identified superior cognitive outcomes in those with LGG treated with radiotherapy or surgery later in the course of their disease compared to those treated at the time of diagnosis (88, 137, 140, 173). Methodological limitations aside, this finding could be the result of patients that received delayed treatment having more time for functional reorganization prior to potential disruption to cognitive networks associated with treatment of their tumor. However, this is purely speculative.

#### Timing of cognitive assessment

The timing of the cognitive assessment in relation to the duration of a patient’s disease, i.e., the length of time since their diagnosis, has been shown in some studies to affect cognitive outcomes in patients with gliomas (125), but not in others (74). In a small prospective study of 25 patients with LGG that received either no oncological treatment (n=16), radiotherapy (n=5), radiotherapy and carboplatin chemotherapy (n=1), or chemotherapy alone (n=3), disease duration as well as the treatment administered influenced cognitive outcomes (125). During the baseline evaluation, patients that had received some form of oncological treatment (chemotherapy/radiotherapy) had impaired performance in motor speed alone (defined as ≥1.5 standard deviations below normative means), but scored one standard deviation below normative values on tests of executive functions. To contrast, patients that had received no oncological treatment had no cognitive impairment. There was a significant variation over time in nonverbal memory (delayed recall), with patients that had received oncological treatment having an improved performance at the six-month point to a level similar to the untreated patients, although both groups of patients (treated and untreated) declined in performance slightly at the 12-month point. However, this study was limited by the small sample size and absence of pre-treatment cognitive assessments. The non-randomized nature of the study may also have influenced the composition of the treated and untreated groups.

In a study of 48 patients with WHO grade II or III oligodendroglioma treated with surgery and either radiotherapy alone (21%) or radiotherapy combined with chemotherapy (79%), cognitive function was evaluated in groups stratified by time since completion of treatment: within the past 2–5 years (mean age at diagnosis 38.27 ± 9.73 years), 6–10 years (mean age at diagnosis 43.08 ± 12.38 years), or more than 10 years ago (mean age at diagnosis 37.77 ± 11.68 years; no significant difference in age at diagnosis between groups was identified). A higher incidence of cognitive impairment was detected in individuals who had completed treatment the longest time ago (173). In patients assessed more than five years after the completion of their treatment, severe cognitive impairment was detected in 38%. Cognitive deficits were found in patients assessed 2–5 years after completion of treatment, but no structural brain abnormalities were detected in this group. Cognitive deterioration was strongly associated with loss of grey matter volume and increased white matter damage.

There also appears to be variation in the cognitive testing results within subjects over time, particularly following surgical intervention or oncological treatment (3, 23, 24, 69, 95–97, 121, 174, 175), which presumably relates to postoperative recovery processes and presumably, to some extent, measurement error. There is also evidence indicating that the rate of recovery of cognitive function after surgery varies by cognitive domain, with language, attention, and executive functions being the slowest domains to recover (93).

Overall, these data highlight the importance of accounting for the timing of cognitive assessment when interpreting the literature, and also for serial assessments to document the cognitive trajectory.

## Discussion

**Figure 2:**
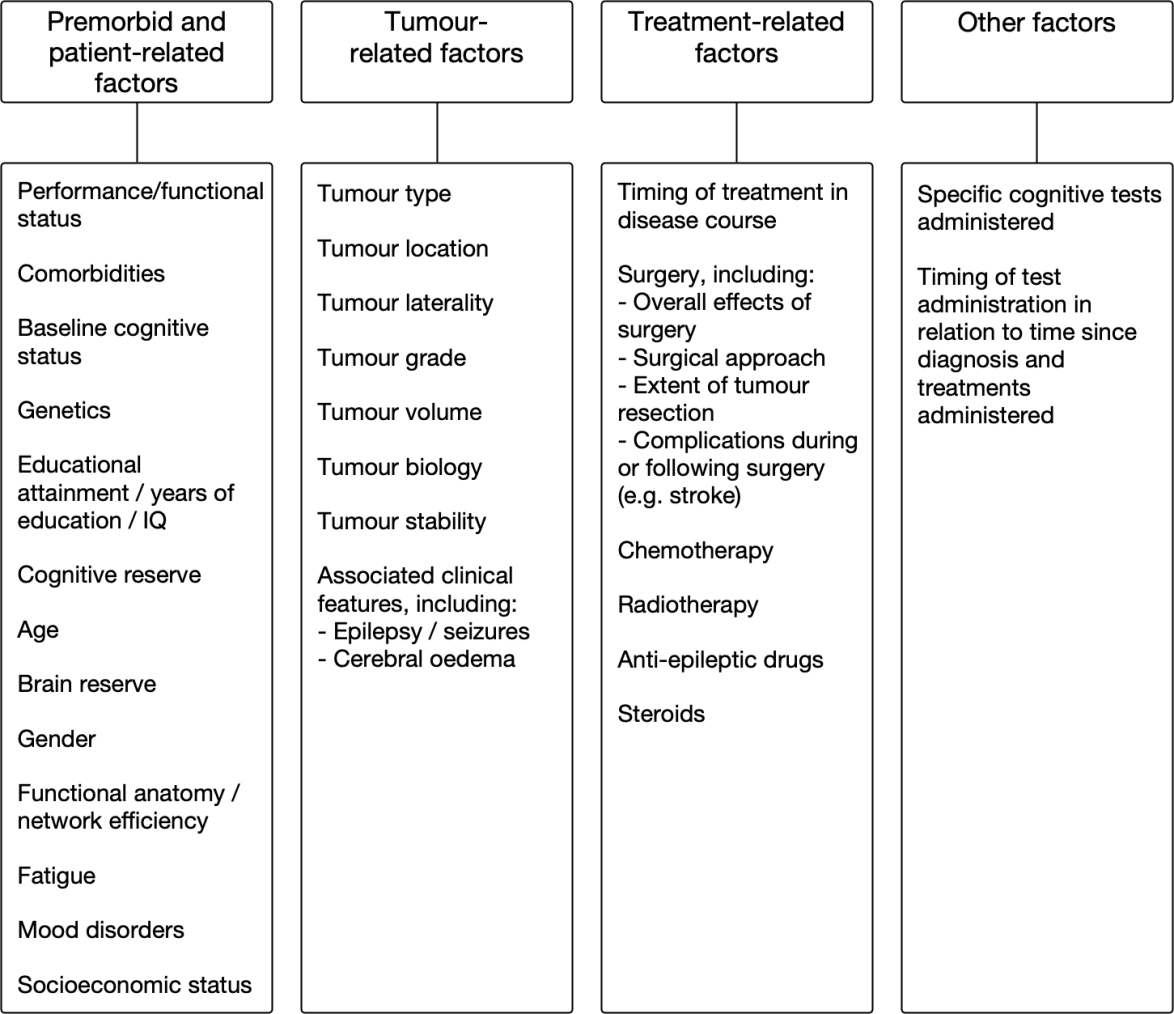
Potential factors associated with cognitive dysfunction in patients with gliomas.

This systematic review aimed to provide a comprehensive overview of the literature relating to influences on cognitive outcomes in patients with gliomas. It has provided a large body of evidence addressing the role of various factors in influencing cognition in patients with gliomas. A summary of the main potential influences on cognitive outcomes is shown in **Figure 2** and **Table 2**; this is a somewhat oversimplified representation of the topic for several reasons. For example, age affects cognitive function but is used as a proxy measure of brain reserve, and thus the two variables are difficult to disentwine. Furthermore, much of the evidence is conflicting, particularly in relation to the role of specific treatments and tumor-related factors in modulating cognitive outcomes. Some studies indicate that tumor location and laterality, tumor volume, and tumor grade are important determinants of cognitive outcomes, and others do not. Tumor location and laterality are likely to be important for cognitive outcomes and the finding of such an association will be dependent on the use of appropriate cognitive tests to detect deficits in the relevant domains. There are studies that suggest that surgery improves cognition, whereas other studies state that surgery makes cognition worse, or that surgery has different effects on different patients, or that surgery initially worsens cognition and over time (a few months) there is a recovery either to the pre-operative baseline or even better than baseline. On balance, most evidence supports the view that surgery has an initial detrimental effect on cognitive outcomes that recovers in most patients over the ensuing months. Clearly, such effects are likely to be influenced by the occurrence of complications such as stroke, as well as by the eloquence of the brain tissue encountered and disrupted by surgery. Discrepancies are also found in relation to the effects of radiotherapy and chemotherapy on cognition, which may be due to differences in study design and specific treatments as well as doses administered, although the evidence supporting the deleterious effects of radiotherapy is strong. Steroid treatment may improve cognition due to resolution of cerebral oedema, but may worsen cognition due to the side effects of the medication itself. Fatigue, gender, and comorbidities may also be important, but have been studied little in patients with gliomas. Mood may or may not influence cognitive outcomes; the evidence is conflicting. Such conflicting findings leave the literature somewhat difficult to interpret, and many unanswered questions remain. For example, proton radiotherapy may be associated with superior cognitive outcomes compared to photon radiotherapy, due to reduced radiation delivery to uninvolved neural tissue associated with the former treatment. Indeed, evidence from children with brain tumors supports this assertion (176–179). However, there are no robust comparative data confirming this in adult glioma patients, requiring further study.

**Table 2:** A summary of the putative influences on cognitive outcomes in patients with gliomas.

Factor		Evidence/Key Points
**Premorbid and patient-related factors**	**Performance/functional status**	Some limited evidence to indicate that this may influence the risk of postoperative cognitive dysfunction (32).
	**Comorbidities**	Hypothyroidism has been associated with impaired MMSE scores (77). Other medical comorbidities are likely to also be relevant.
	**Baseline cognitive status**	Likely to be an important predictor of cognitive outcomes (71, 79, 80).
	**Genetics**	Polymorphisms in COMT, BDNF, and DRD2 genes may be associated with cognitive performance in specific domains (147). IDH mutations may also be associated with improved cognitive outcomes (7). Polymorphisms in inflammation, DNA repair, and metabolism pathways may also be associated with cognitive function (148). APOE status has been shown to be associated with cognitive outcomes in some (126, 131) but not all (150) studies.
	**Educational attainment/cognitive reserve**	Cognitive reserve is most commonly evaluated *via* proxy, typically years of formal education, with mostly no effect on cognitive outcomes observed (28, 39, 72, 171), but there are exceptions (71) and it may be relevant for HGG but not LGG (42). Higher education levels may predict responsiveness to cognitive rehabilitation (172). NART IQ, a measure of premorbid IQ, is another proxy measure of cognitive reserve and has been positively associated with cognitive outcomes in some (73, 171) but not all (72) studies.
	**Age/brain reserve**	Age is the commonest proxy measure for brain reserve, which may (28, 71–73) or may not (38, 61, 74) influence cognitive outcomes. Evidence for and against a role for age in influencing cognition comes from prospective studies. Younger age may predict responsiveness to cognitive rehabilitation (172).
	**Gender**	Limited evidence. One study indicated improved language performance in females immediately after surgery, but scores were comparable to males one year after surgery (78).
	**Functional anatomy**	Changes to the white matter tracts and functional networks of the brain (81) and arcuate fasciculus lateralization (82) may influence cognitive function, particularly language outcomes.
	**Fatigue**	No strong evidence that it influences cognitive outcomes specifically in brain tumour patients, but fatigue is common in patients with brain tumours (162) and is a common side effect of several anti-epileptic drugs (163). Further studies are required.
	**Mood disorders**	There is some evidence for an association between mood state in cognitive outcomes (8, 26, 64, 74, 169), but whether mood changes influence cognitive outcomes or vice versa remains to be fully elucidated.
**Tumour-related factors**	**Tumour location**	Conflicting results, with evidence for (23–35) and against (3, 36–40) a role in cognitive outcomes, but on balance it is most likely important. Likely to depend on the cognitive domain evaluated (41).
	**Tumour laterality**	Many studies suggest right-sided tumours are associated with a lower risk of cognitive impairment (15, 33, 43–49), sometimes irrespective of the exact location of the tumour within the hemisphere (50), but this may depend on the cognitive modality under assessment (51–55). Other studies disagree with the relevance of laterality (36, 56, 57), but overall tumour laterality is likely to influence cognitive outcomes.
	**Tumour grade**	Evidence for (4, 32, 42, 54, 62–67) and against (15, 40, 50, 60, 68, 69) a role of tumour grade in influencing cognitive outcomes; others indicate it may be important for some but not all cognitive domains (41). Overall, there is more evidence supporting a role of tumour grade in cognitive outcomes than evidence against a link.
	**Tumour volume**	Some evidence for a role of tumour volume on cognitive outcomes (4, 54, 60), but there is more evidence against such a role (3, 15, 36, 39, 42, 47, 52, 61). It is likely that this variable has complex interactions with other putative influences on cognitive outcomes, including extent of tumour resection and surgical approach.
	**Tumour biology**	Molecular and histopathological profiles may (7, 52, 54, 70) or may not (41, 60) influence cognitive outcomes. The conflicting findings may result from differences in the specific tumour characteristics studied and differences in study design.
	**Associated clinical features, including:** **Epilepsy/seizures** **Cerebral oedema Hydrocephalus**	Epilepsy may influence cognitive outcomes but evidence suggests the relationship is driven primarily by the use of anti-epileptic drugs (AEDs; see below) (152). The limited evidence supporting improved cognition in patients receiving corticosteroids are likely to be due to the resolution of cerebral oedema (49) (see below). Hydrocephalus is known to be associated with cognitive dysfunction but the specific relationship in patients with gliomas is poorly studied to date.
**Treatment-related factors**	**Timing of treatment in disease course**	Superior cognitive outcomes have been observed in those with LGG treated with radiotherapy or surgery later in the course of their disease compared to those treated at the time of diagnosis (88, 137, 140, 173).
	**Surgery**	**Overall effects of surgery** Large variation in findings. Several patterns observed, including: - a deterioration in cognition following surgery often with partial or full recovery or long-term improvement in the ensuing months (3, 24, 33, 41, 47, 48, 64, 71, 78, 86–100) - an improvement in cognition (37, 52, 101–103) - no effect of surgery at all (28, 37, 68, 104–106) - deterioration in specific cognitive domains but no overall cognitive impairment at the group level (108) - a mixed picture: some patients experiencing cognitive improvement and others in the same study who experience cognitive decline or no change (39, 58, 60, 76, 110–113) - or improvements in some cognitive domains and deterioration (2) and/or no change (46) in others Overall, surgery influences cognition negatively initially followed by a recovery over several months in most cases. **Surgical approach** Limited specific evidence to indicate that this influences cognitive outcomes apart from use of awake craniotomies/intraoperative brain mapping having a positive (93, 118, 119) or negative (57) influence, although there is likely overlap with evidence for tumour location, which is a crucial influence on surgical approach undertaken. **Extent of tumour resection** A number of studies indicate that higher extent of tumour resection does not negatively influence cognitive outcomes (3, 4, 36, 38, 47, 49, 50, 52, 60), and some evidence it may positively influence cognitive outcomes (116). **Complications during or following surgery** Evidence to suggest the development of infarcts are associated with worse cognitive outcomes following surgery (93, 114, 115).
	**Chemotherapy**	Most evidence indicates no effect on cognition (3, 68, 123, 124), but may have a negative (18) or a positive effect (122). Likely to be influenced by several variables including the specific chemotherapy regime chosen and timing of administration.
	**Radiotherapy**	One of the factors most strongly associated with adverse cognition in patients with brain tumour (32, 39, 129–138), with neuroimaging correlates of the cognitive effects identified (131, 132, 135, 137), but not all studies have found such a relationship (3, 28, 68, 139–141), and in some cases mixed results (61, 142–144) and improvements in cognition following radiotherapy (122) have been noted. Effects of radiotherapy on cognitive outcomes are likely to relate to the specific anatomical structures that have been irradiated and their laterality (136, 138). Studies comparing combinations or radiotherapy and/or chemotherapy treatments suggest no difference in cognitive outcomes according to oncological treatment administered (122, 146), but follow-up in these studies was short.
	**Anti-epileptic drugs**	AEDs are a well-recognised cause of cognitive decline (36, 46, 49, 131, 133, 153, 154), particularly the older anti-epileptic medications (155), and use of multiple AEDs in a single patient increases the risk further (36). Limited data suggest no influence of seizures or anti-epileptic medications on cognitive outcomes in patients with brain tumours (61, 63, 157), but these studies include limitations of small sample size (157) and lack of comprehensive cognitive assessment (use of MMSE alone) (61). Overall, most evidence supports its role in cognition.
	**Steroids**	Well-recognised cognitive effects, particularly in studies of patients with systemic conditions requiring steroid treatment, but also in patients with gliomas (161). Some evidence against a role of steroid use in determining cognitive outcomes (63). Evidence of improved cognition with steroid use (49) is likely secondary to resolution of cerebral oedema. Role is likely to be influenced by the presence and extent of cerebral oedema, and the effects of that on cognition.
**Other**	**Specific cognitive tests administered**	Many studies identified in the systematic review found impaired performance in some but not all cognitive tests administered to patients. Furthermore, evidence indicates that the rate of recovery of cognitive function after surgery varies by cognitive domain, with language, attention, and executive functions being the slowest domains to recover (93). This highlights the importance of administering comprehensive test batteries to patients to ensure all cognitive changes are captured.
	**Timing of test administration**	Length of time since diagnosis has been shown in some studies to affect cognitive outcomes in patients with gliomas (125), but not in others (74). This may be in part due to the timing in relation to treatments administered.

To contrast, baseline cognitive status appears to be a consistent factor that influences cognitive outcomes, with worse baseline cognition at diagnosis/pre-treatment correlated with worse long-term outcomes. Similarly, there is evidence that anti-epileptic drugs have a negative effect on cognition. Delaying treatment for LGG where possible appears to halt cognitive deterioration. Several studies indicate a role for genetics, particularly in relation to APOE status, but further studies are needed to confirm this. Cognitive and brain reserve have not been well-studied in the context of gliomas; there is evidence to suggest that age and NART IQ can influence cognitive outcomes, and that years of education do not, although not all studies are in agreement with this.

**Figure 3:**
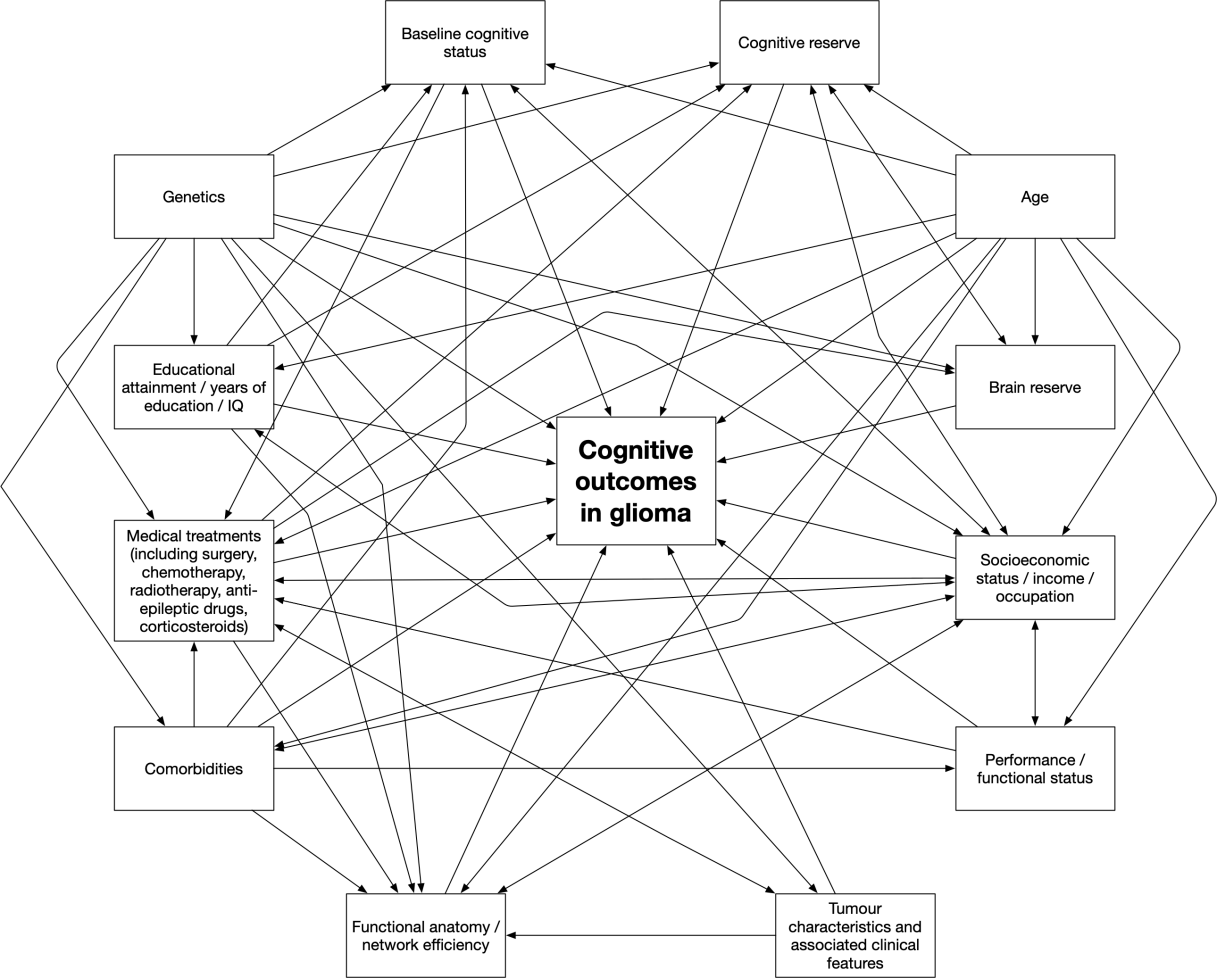
A proposed theoretical causal depiction of putative pathways related to cognitive outcomes in patients with gliomas.

The conflicting findings may be the result of wide heterogeneity in the methods used between studies, and the wide variation in the timing of the cognitive testing. Crucially, the potentially important role of baseline sociodemographic variables has been relatively neglected in the glioma literature. It is of note that several of the studies had collected data on occupation or social class from participants, but this was usually presented in a table without any analysis, for example through incorporating it as a covariate. These variables may explain some of the variability in the results obtained between studies and explain the variability in cognitive outcomes seen in clinical practice. Several of the putative influences on cognitive outcomes are likely to interact, and **Figure 3** provides a theoretical causal depiction of putative pathways.

There are several limitations affecting the interpretation of this systematic review. First, the studies have used varying cognitive tests, performed the tests at different time points following completion of treatment, and have varying lengths of follow-up. Some of the studies relied on a single measure of cognitive function, including the MMSE (itself not a comprehensive assessment of cognitive function), despite evidence that reliable results are only obtained with a comprehensive neuropsychological battery (180) and evidence that screening tools such as the MMSE and Montreal Cognitive Assessment (MoCA) do not detect all instances of cognitive impairment (2). The Response Assessment in Neuro-oncology (RANO) Group have provided recommendations about which cognitive tests should be administered in trials of patients with diffuse LGGs; this includes the MMSE, Hopkins Verbal Learning Test-Revised (HVLT-R), TMT parts A and B, and Multilingual Aphasia Examination Controlled Oral Word Association (MAE-COWA) test at baseline assessment, and the HVLT-R, TMT parts A and B, and MAE-COWA test during follow-up assessments (6). It is the authors’ opinion that these should be seen as a minimum set of tests to be used in studies evaluating cognitive function. Some studies did not clearly specify when the cognitive testing took place (27, 28, 89, 120, 142, 152). It is also difficult to disentangle the contributions of each of the different factors towards cognitive dysfunction; for example, how much are the cognitive changes due to the natural history of the disease itself, particularly when residual tumor has been left after surgery, versus other causes such as the specific treatments administered. Furthermore, patients in the same study have also had different treatments, which makes it difficult to identify specific contributions to a patient’s cognitive status. Some manuscripts did not detail the exact type and extent of surgery undertaken, which is important because (i) a biopsy is likely to result in different cognitive outcomes than tumor resection, but also (ii) grouping those that underwent debulking or resection of the tumor together without accounting for the amount of tumor removed (for example, 25% versus 100%) is challenging because the proportion of tumor removed may have a clinically meaningful effect on cognitive outcomes. There was also a lack of systematic reporting of treatments received by patients, with some patients not detailing how many participants had received chemotherapy and/or radiotherapy. There was also variation in the doses of such treatments received within and between studies. **Box 1** provides a summary checklist of information that should be provided in future studies in the field to allow the systematic accumulation of clinically relevant data.

Box 1 **Checklist of essential information that should be provided in future studies in the field to allow the systematic accumulation of clinically relevant data**

**Premorbid and patient-related factors**

- Demographic information including age, gender, and ethnicity
- Handedness
- Socioeconomic status
- Educational attainment/IQ
- Baseline cognitive status
- Genetic profile where available
- Evaluation for fatigue and mood disorders
- Clinical symptoms
- Medication history
- Comorbidities
- Alcohol and tobacco consumption
- Performance/functional status
- Functional anatomy (including speech laterality/hemisphere dominance)

**Tumour-related factors**

- Tumour type
- Tumour location
- Tumour laterality
- Tumour grade
- Tumour volume
- Tumour biology
- Tumour stability
- Associated clinical features (epilepsy/seizures, hydrocephalus, cerebral oedema)

**Treatment-related factors**

- Surgery undertaken, including type of surgery and surgical approach (asleep versus awake, use of brain mapping pre- and intraoperatively), extent of tumour resection obtained *via* surgery, complications associated with surgery
- Chemotherapy administered, including drug(s), dosage(s), route and length of administration
- Radiotherapy administered, including method of delivery, dosage(s), targets and length of administration
- Anti-epileptic drug usage, including specific drug(s), dosage(s), route and length of administration
- Steroid usage, including specific drug(s), dosage(s), route and length of administration
- Timing of all of the relevant above treatments (since diagnosis)

**Other factors**

- Cognitive tests administered, ideally a battery of validated robust measures covering broad cognitive domains
- Specific details of the timing of cognitive test administration in relation to the time since diagnosis and treatments administered

An important additional limitation is that many of the studies identified in this systematic review incorporated a range of pathologies into a single group for analysis; for example, one study created a group of low-grade supratentorial brain tumors that incorporated WHO grade I and II gliomas, pituitary and pineal tumors, and non-invasive meningiomas (129). There was also unclear classification of tumors within studies; for example, one study had a ‘LGG’ category of tumors in addition to a separate category for ‘pilocytic astrocytoma’, which is a type of LGG, with no definition of what glioma types comprised the LGG group (92). There is evidence from studies including multiple tumor pathologies that differences exist in cognitive outcomes according to tumor type (66, 67), even when considering tumors in the same anatomical location (46). The underlying mechanisms through which these different tumors can cause cognitive dysfunction are likely to vary, and the effects of surgery are also likely to vary because of their typical locations within the skull (for example, most meningiomas are on the outside of the skull and do not require the surgeon to enter the brain to remove them). Different tumors may exert different effects on cognitive function due to different effects on functional brain tissue, which may be a reflection of the underlying pathophysiology of the disease process or external compression on neural structures altering their function. The theory that cognitive differences may result from tumor type and surgical approach is supported by the findings of a prospective study of 66 patients with either HGG, LGG, or meningiomas, all of whom underwent the same neuropsychological testing focusing on perception and interpretation of emotion (95); the high-grade glioma patients were largely already impaired in the more perceptual tasks before surgery and surgery did not influence their performance. LGG patients, however, who were unimpaired before surgery, showed a significant deficit in perceptual tasks immediately after surgery that largely recovered at the point of a repeat assessment approximately four months after surgery. To contrast, meningioma patients were largely unimpaired in all tasks. Similar results were reported in another study evaluating memory function published by the same group (66).

Tests used to identify cognitive dysfunction may not detect the true extent of morbidity related to brain tumors. For example, in one study evaluating the longer-term effects of surgery on spontaneous speech in patients with HGG and LGG found that surgery deteriorated the quality of spontaneous speech over the period studied (up to 12 months following surgery), but not the performance at the test level (40). This suggests that spontaneous speech has additional value to standardized tests for diagnosing language impairments in brain tumor patients.

## Conclusions

Cognitive dysfunction is common in people living with, or with a history of, glioma and can have a profound effect on their quality of life. This systematic review confirms that multiple factors influence cognitive outcomes in patients with gliomas and are likely to interact in a complex manner as shown in **Figure 3**. Wide differences in study methodologies call for more homogenous study design and study reporting in future studies, using the criteria listed in **Box 1**. The effects of tumour characteristics such as location and laterality, as well as treatments administered, are some of the most studied variables, but the evidence for these is conflicting, which may be the result of methodological and study population differences. Overall, however, tumor location and laterality, surgery, and radiotherapy are tumor- and treatment-related variables with some of the strongest evidence base. Baseline cognitive status appears to be a consistent factor that influences cognitive outcomes, with worse baseline cognition at diagnosis/pre-treatment correlated with worse long-term outcomes. Similarly, much evidence indicates that anti-epileptic drugs have a negative effect on cognition. Genetics also appears to have a role. Many unanswered questions remain. For example, cognitive reserve, brain reserve, and socioeconomic status as well as a number of other variables discussed in this review, and their influence on cognition and recovery, have not been well-studied in the context of gliomas and are areas for focus in future research.

## Data availability statement

The original contributions presented in the study are included in the article/**Supplementary Material**. Further inquiries can be directed to the corresponding author.

## Author contributions

MK, MT, and AT contributed to the conception and design of the review. MK performed the literature search. MK and BH screened the titles and abstracts. MK extracted the data and wrote the first draft of the manuscript. All authors contributed to manuscript revision, read, and approved the submitted version.

## Funding

MK is supported by a studentship from the Economic and Social Research Council awarded *via* University College London (studentship reference: 1924548; grant reference: ES/P000592/1). For the purpose of open access, the author has applied a Creative Commons Attribution (CC BY) license to any Author Accepted Manuscript version arising.

## Conflict of interest

The authors declare that the research was conducted in the absence of any commercial or financial relationships that could be construed as a potential conflict of interest.

## Publisher’s note

All claims expressed in this article are solely those of the authors and do not necessarily represent those of their affiliated organizations, or those of the publisher, the editors and the reviewers. Any product that may be evaluated in this article, or claim that may be made by its manufacturer, is not guaranteed or endorsed by the publisher.
