# Supplementary material for: Influences on cognitive outcomes in adult patients with gliomas: A systematic review
Source: Front Oncol. 2022 Aug 5;12:943600. doi: 10.3389/fonc.2022.943600 (PMC9407441; doi:10.3389/fonc.2022.943600)
Supplement: Supplementary file 1 [file Table_1.docx]

**Supplementary Table 1. Search terms for systematic review**

**MeSH Headings**

| **#** | **Subject heading** | **Tree number(s)** | **Entry terms** | **MeSH Tree Structure** |
| --- | --- | --- | --- | --- |
| **1** | **Brain neoplasms** | C04.588.614.250.195  C10.228.140.211  C10.551.240.250 | Benign Neoplasms, Brain  Brain Cancer  Brain Neoplasm, Primary  Brain Neoplasms, Benign  Brain Neoplasms, Malignant  Brain Neoplasms, Malignant, Primary  Brain Neoplasms, Primary Malignant  Brain Tumor, Primary  Brain Tumor, Recurrent  Brain Tumors  Cancer of Brain  Cancer of the Brain  Intracranial Neoplasms  Malignant Neoplasms, Brain  Malignant Primary Brain Neoplasms  Malignant Primary Brain Tumors  Neoplasms, Brain  Neoplasms, Brain, Benign  Neoplasms, Brain, Malignant  Neoplasms, Brain, Primary  Neoplasms, Intracranial  Primary Brain Neoplasms  Primary Malignant Brain Neoplasms  Primary Malignant Brain Tumors | [**Brain Neoplasms [C04.588.614.250.195]**](https://meshb.nlm.nih.gov/record/ui?ui=D001932)   - [Cerebral Ventricle Neoplasms [C04.588.614.250.195.205]](https://meshb.nlm.nih.gov/record/ui?ui=D002551) - [Infratentorial Neoplasms [C04.588.614.250.195.411]](https://meshb.nlm.nih.gov/record/ui?ui=D015192) - [Neurocytoma [C04.588.614.250.195.648]](https://meshb.nlm.nih.gov/record/ui?ui=D018306) - [Pinealoma [C04.588.614.250.195.766]](https://meshb.nlm.nih.gov/record/ui?ui=D010871) - [Supratentorial Neoplasms [C04.588.614.250.195.885]](https://meshb.nlm.nih.gov/record/ui?ui=D015173)   [**Brain Neoplasms [C10.551.240.250]**](https://meshb.nlm.nih.gov/record/ui?ui=D001932)   - [Cerebral Ventricle Neoplasms [C10.551.240.250.200]](https://meshb.nlm.nih.gov/record/ui?ui=D002551) - [Infratentorial Neoplasms [C10.551.240.250.400]](https://meshb.nlm.nih.gov/record/ui?ui=D015192) - [Neurocytoma [C10.551.240.250.550]](https://meshb.nlm.nih.gov/record/ui?ui=D018306) - [Pinealoma [C10.551.240.250.625]](https://meshb.nlm.nih.gov/record/ui?ui=D010871) - [Supratentorial Neoplasms [C10.551.240.250.700]](https://meshb.nlm.nih.gov/record/ui?ui=D015173)   [**Brain Neoplasms [C10.228.140.211]**](https://meshb.nlm.nih.gov/record/ui?ui=D001932)   - [Cerebral Ventricle Neoplasms [C10.228.140.211.280]](https://meshb.nlm.nih.gov/record/ui?ui=D002551) - [Infratentorial Neoplasms [C10.228.140.211.500]](https://meshb.nlm.nih.gov/record/ui?ui=D015192) - [Neurocytoma [C10.228.140.211.692]](https://meshb.nlm.nih.gov/record/ui?ui=D018306) - [Pinealoma [C10.228.140.211.788]](https://meshb.nlm.nih.gov/record/ui?ui=D010871) - [Supratentorial Neoplasms [C10.228.140.211.885]](https://meshb.nlm.nih.gov/record/ui?ui=D015173) |
| **2** | **Neoplasms, Neuroepithelial** | C04.557.465.625.600  C04.557.470.670  C04.557.580.625.600 | Astroblastoma  Ependymoastrocytoma  Gliomatosis Cerebri  Neuroepithelial Neoplasms  Neuroepithelial Tumors  Spongioblastoma, Polar | [**Neoplasms, Neuroepithelial [C04.557.470.670]**](https://meshb.nlm.nih.gov/record/ui?ui=D018302)   - [Ganglioneuroma [C04.557.470.670.355]](https://meshb.nlm.nih.gov/record/ui?ui=D005729) - [Glioma [C04.557.470.670.380]](https://meshb.nlm.nih.gov/record/ui?ui=D005910)   - [Astrocytoma [C04.557.470.670.380.080]](https://meshb.nlm.nih.gov/record/ui?ui=D001254)     - [Glioblastoma [C04.557.470.670.380.080.335]](https://meshb.nlm.nih.gov/record/ui?ui=D005909)   - [Ependymoma [C04.557.470.670.380.290]](https://meshb.nlm.nih.gov/record/ui?ui=D004806)     - [Glioma, Subependymal [C04.557.470.670.380.290.390]](https://meshb.nlm.nih.gov/record/ui?ui=D018315)   - [Ganglioglioma [C04.557.470.670.380.350]](https://meshb.nlm.nih.gov/record/ui?ui=D018303)   - [Gliosarcoma [C04.557.470.670.380.400]](https://meshb.nlm.nih.gov/record/ui?ui=D018316)   - [Medulloblastoma [C04.557.470.670.380.515]](https://meshb.nlm.nih.gov/record/ui?ui=D008527)   - [Oligodendroglioma [C04.557.470.670.380.590]](https://meshb.nlm.nih.gov/record/ui?ui=D009837)   - [Optic Nerve Glioma [C04.557.470.670.380.795]](https://meshb.nlm.nih.gov/record/ui?ui=D020339) - [Neurocytoma [C04.557.470.670.580]](https://meshb.nlm.nih.gov/record/ui?ui=D018306) - [Neuroectodermal Tumors, Primitive [C04.557.470.670.590]](https://meshb.nlm.nih.gov/record/ui?ui=D018242)   - [Medulloblastoma [C04.557.470.670.590.500]](https://meshb.nlm.nih.gov/record/ui?ui=D008527)   - [Neuroectodermal Tumors, Primitive, Peripheral [C04.557.470.670.590.650]](https://meshb.nlm.nih.gov/record/ui?ui=D018241) - [Pinealoma [C04.557.470.670.657]](https://meshb.nlm.nih.gov/record/ui?ui=D010871) - [Retinoblastoma [C04.557.470.670.725]](https://meshb.nlm.nih.gov/record/ui?ui=D012175) |
| **3** | **Neoplasm Metastasis** | C04.697.650  C23.550.727.650 | Metastases, Neoplasm  Metastasis  Metastasis, Neoplasm  Neoplasm Metastases | [**Neoplasm Metastasis [C04.697.650]**](https://meshb.nlm.nih.gov/record/ui?ui=D009362)   - [Lymphatic Metastasis [C04.697.650.560]](https://meshb.nlm.nih.gov/record/ui?ui=D008207) - [Neoplasm Micrometastasis [C04.697.650.695]](https://meshb.nlm.nih.gov/record/ui?ui=D061206) - [Neoplasm Seeding [C04.697.650.830]](https://meshb.nlm.nih.gov/record/ui?ui=D009366) - [Neoplasms, Unknown Primary [C04.697.650.895]](https://meshb.nlm.nih.gov/record/ui?ui=D009382) - [Neoplastic Cells, Circulating [C04.697.650.900]](https://meshb.nlm.nih.gov/record/ui?ui=D009360)   [**Neoplasm Metastasis [C23.550.727.650]**](https://meshb.nlm.nih.gov/record/ui?ui=D009362)   - [Lymphatic Metastasis [C23.550.727.650.560]](https://meshb.nlm.nih.gov/record/ui?ui=D008207) - [Neoplasm Micrometastasis [C23.550.727.650.695]](https://meshb.nlm.nih.gov/record/ui?ui=D061206) - [Neoplasm Seeding [C23.550.727.650.830]](https://meshb.nlm.nih.gov/record/ui?ui=D009366) - [Neoplasms, Unknown Primary [C23.550.727.650.895]](https://meshb.nlm.nih.gov/record/ui?ui=D009382) - [Neoplastic Cells, Circulating [C23.550.727.650.900]](https://meshb.nlm.nih.gov/record/ui?ui=D009360) |
| **4** | **Mental processes** | F02.463 |  | - [Mental Processes [F02.463]](https://meshb.nlm.nih.gov/record/ui?ui=D008606)   - [Anticipation, Psychological [F02.463.093]](https://meshb.nlm.nih.gov/record/ui?ui=D059031)   - [Cognition [F02.463.188]](https://meshb.nlm.nih.gov/record/ui?ui=D003071)     - [Awareness [F02.463.188.150]](https://meshb.nlm.nih.gov/record/ui?ui=D001364)     - [Cognitive Dissonance [F02.463.188.305]](https://meshb.nlm.nih.gov/record/ui?ui=D003073)     - [Cognitive Reserve [F02.463.188.331]](https://meshb.nlm.nih.gov/record/ui?ui=D058245)     - [Comprehension [F02.463.188.357]](https://meshb.nlm.nih.gov/record/ui?ui=D032882)     - [Consciousness [F02.463.188.409]](https://meshb.nlm.nih.gov/record/ui?ui=D003243)     - [Imagination [F02.463.188.634]](https://meshb.nlm.nih.gov/record/ui?ui=D007092)     - [Intuition [F02.463.188.675]](https://meshb.nlm.nih.gov/record/ui?ui=D019545)     - [Metacognition [F02.463.188.756]](https://meshb.nlm.nih.gov/record/ui?ui=D000067489)   - [Executive Function [F02.463.217]](https://meshb.nlm.nih.gov/record/ui?ui=D056344)   - [Higher Nervous Activity [F02.463.247]](https://meshb.nlm.nih.gov/record/ui?ui=D006613)   - [Intention [F02.463.306]](https://meshb.nlm.nih.gov/record/ui?ui=D033182)   - [Learning [F02.463.425]](https://meshb.nlm.nih.gov/record/ui?ui=D007858)     - [Association [F02.463.425.069]](https://meshb.nlm.nih.gov/record/ui?ui=D001244)     - [Avoidance Learning [F02.463.425.097]](https://meshb.nlm.nih.gov/record/ui?ui=D001362)     - [Conditioning (Psychology) [F02.463.425.179]](https://meshb.nlm.nih.gov/record/ui?ui=D003213)     - [Critical Period (Psychology) [F02.463.425.209]](https://meshb.nlm.nih.gov/record/ui?ui=D003423)     - [Cues [F02.463.425.234]](https://meshb.nlm.nih.gov/record/ui?ui=D003463)     - [Discrimination Learning [F02.463.425.280]](https://meshb.nlm.nih.gov/record/ui?ui=D004193)     - [Generalization (Psychology) [F02.463.425.357]](https://meshb.nlm.nih.gov/record/ui?ui=D005793)     - [Habituation, Psychophysiologic [F02.463.425.393]](https://meshb.nlm.nih.gov/record/ui?ui=D006185)     - [Helplessness, Learned [F02.463.425.420]](https://meshb.nlm.nih.gov/record/ui?ui=D006380)     - [Imprinting (Psychology) [F02.463.425.448]](https://meshb.nlm.nih.gov/record/ui?ui=D007173)     - [Inhibition (Psychology) [F02.463.425.475]](https://meshb.nlm.nih.gov/record/ui?ui=D007266)     - [Memory [F02.463.425.540]](https://meshb.nlm.nih.gov/record/ui?ui=D008568)     - [Neurolinguistic Programming [F02.463.425.575]](https://meshb.nlm.nih.gov/record/ui?ui=D020557)     - [Overlearning [F02.463.425.612]](https://meshb.nlm.nih.gov/record/ui?ui=D010056)     - [Practice (Psychology) [F02.463.425.674]](https://meshb.nlm.nih.gov/record/ui?ui=D011214)     - [Probability Learning [F02.463.425.701]](https://meshb.nlm.nih.gov/record/ui?ui=D011337)     - [Problem-Based Learning [F02.463.425.720]](https://meshb.nlm.nih.gov/record/ui?ui=D018794)     - [Problem Solving [F02.463.425.725]](https://meshb.nlm.nih.gov/record/ui?ui=D011340)     - [Reinforcement (Psychology) [F02.463.425.770]](https://meshb.nlm.nih.gov/record/ui?ui=D012054)     - [Reversal Learning [F02.463.425.798]](https://meshb.nlm.nih.gov/record/ui?ui=D012193)     - [Set (Psychology) [F02.463.425.838]](https://meshb.nlm.nih.gov/record/ui?ui=D012718)     - [Spatial Learning [F02.463.425.874]](https://meshb.nlm.nih.gov/record/ui?ui=D065853)     - [Transfer (Psychology) [F02.463.425.910]](https://meshb.nlm.nih.gov/record/ui?ui=D014163)     - [Verbal Learning [F02.463.425.952]](https://meshb.nlm.nih.gov/record/ui?ui=D014706)   - [Mind-Body Relations, Metaphysical [F02.463.509]](https://meshb.nlm.nih.gov/record/ui?ui=D019222)   - [Mindfulness [F02.463.551]](https://meshb.nlm.nih.gov/record/ui?ui=D064866)   - [Perception [F02.463.593]](https://meshb.nlm.nih.gov/record/ui?ui=D010465)     - [Auditory Perception [F02.463.593.071]](https://meshb.nlm.nih.gov/record/ui?ui=D001307)     - [Body Image [F02.463.593.112]](https://meshb.nlm.nih.gov/record/ui?ui=D001828)     - [Depth Perception [F02.463.593.200]](https://meshb.nlm.nih.gov/record/ui?ui=D003867)     - [Discrimination (Psychology) [F02.463.593.257]](https://meshb.nlm.nih.gov/record/ui?ui=D004192)     - [Eidetic Imagery [F02.463.593.292]](https://meshb.nlm.nih.gov/record/ui?ui=D004538)     - [Field Dependence-Independence [F02.463.593.343]](https://meshb.nlm.nih.gov/record/ui?ui=D005364)     - [Form Perception [F02.463.593.373]](https://meshb.nlm.nih.gov/record/ui?ui=D005556)     - [Illusions [F02.463.593.446]](https://meshb.nlm.nih.gov/record/ui?ui=D007088)     - [Interoception [F02.463.593.465]](https://meshb.nlm.nih.gov/record/ui?ui=D065812)     - [Olfactory Perception [F02.463.593.485]](https://meshb.nlm.nih.gov/record/ui?ui=D055696)     - [Pain Perception [F02.463.593.504]](https://meshb.nlm.nih.gov/record/ui?ui=D058748)     - [Pattern Recognition, Physiological [F02.463.593.524]](https://meshb.nlm.nih.gov/record/ui?ui=D046709)     - [Perceptual Distortion [F02.463.593.603]](https://meshb.nlm.nih.gov/record/ui?ui=D010469)     - [Sensory Deprivation [F02.463.593.696]](https://meshb.nlm.nih.gov/record/ui?ui=D012683)     - [Sensory Thresholds [F02.463.593.710]](https://meshb.nlm.nih.gov/record/ui?ui=D012684)     - [Size Perception [F02.463.593.725]](https://meshb.nlm.nih.gov/record/ui?ui=D012858)     - [Social Perception [F02.463.593.752]](https://meshb.nlm.nih.gov/record/ui?ui=D012938)     - [Space Perception [F02.463.593.778]](https://meshb.nlm.nih.gov/record/ui?ui=D013028)     - [Taste Perception [F02.463.593.817]](https://meshb.nlm.nih.gov/record/ui?ui=D055697)     - [Time Perception [F02.463.593.857]](https://meshb.nlm.nih.gov/record/ui?ui=D013998)     - [Touch Perception [F02.463.593.894]](https://meshb.nlm.nih.gov/record/ui?ui=D055698)     - [Visual Perception [F02.463.593.932]](https://meshb.nlm.nih.gov/record/ui?ui=D014796)     - [Weight Perception [F02.463.593.959]](https://meshb.nlm.nih.gov/record/ui?ui=D014892)   - [Spatial Navigation [F02.463.641]](https://meshb.nlm.nih.gov/record/ui?ui=D065854)   - [Theory of Mind [F02.463.689]](https://meshb.nlm.nih.gov/record/ui?ui=D056345)   - [Thinking [F02.463.785]](https://meshb.nlm.nih.gov/record/ui?ui=D013850)   - [Volition [F02.463.902]](https://meshb.nlm.nih.gov/record/ui?ui=D014836) |
| **5** | **Neurobehavioral manifestation** | C10.597.606  C23.888.592.604  F01.700 | Cognitive Manifestations  Cognitive Symptoms  Neurobehavioral Signs and Symptoms  Signs and Symptoms, Neurobehavioral | [**Neurobehavioral Manifestations [C10.597.606]**](https://meshb.nlm.nih.gov/record/ui?ui=D019954)   - [Anhedonia [C10.597.606.057]](https://meshb.nlm.nih.gov/record/ui?ui=D059445) - [Catatonia [C10.597.606.115]](https://meshb.nlm.nih.gov/record/ui?ui=D002389) - [Communication Disorders [C10.597.606.150]](https://meshb.nlm.nih.gov/record/ui?ui=D003147) - [Confusion [C10.597.606.337]](https://meshb.nlm.nih.gov/record/ui?ui=D003221) - [Consciousness Disorders [C10.597.606.358]](https://meshb.nlm.nih.gov/record/ui?ui=D003244) - [Lethargy [C10.597.606.441]](https://meshb.nlm.nih.gov/record/ui?ui=D053609) - [Memory Disorders [C10.597.606.525]](https://meshb.nlm.nih.gov/record/ui?ui=D008569) - [Intellectual Disability [C10.597.606.643]](https://meshb.nlm.nih.gov/record/ui?ui=D008607) - [Perceptual Disorders [C10.597.606.762]](https://meshb.nlm.nih.gov/record/ui?ui=D010468) - [Psychomotor Disorders [C10.597.606.881]](https://meshb.nlm.nih.gov/record/ui?ui=D011596)   [**Neurobehavioral Manifestations [C23.888.592.604]**](https://meshb.nlm.nih.gov/record/ui?ui=D019954)   - [Anhedonia [C23.888.592.604.039]](https://meshb.nlm.nih.gov/record/ui?ui=D059445) - [Catatonia [C23.888.592.604.115]](https://meshb.nlm.nih.gov/record/ui?ui=D002389) - [Communication Disorders [C23.888.592.604.150]](https://meshb.nlm.nih.gov/record/ui?ui=D003147) - [Confusion [C23.888.592.604.339]](https://meshb.nlm.nih.gov/record/ui?ui=D003221) - [Consciousness Disorders [C23.888.592.604.359]](https://meshb.nlm.nih.gov/record/ui?ui=D003244) - [Lethargy [C23.888.592.604.444]](https://meshb.nlm.nih.gov/record/ui?ui=D053609) - [Memory Disorders [C23.888.592.604.529]](https://meshb.nlm.nih.gov/record/ui?ui=D008569) - [Intellectual Disability [C23.888.592.604.646]](https://meshb.nlm.nih.gov/record/ui?ui=D008607) - [Perceptual Disorders [C23.888.592.604.764]](https://meshb.nlm.nih.gov/record/ui?ui=D010468) - [Psychomotor Disorders [C23.888.592.604.882]](https://meshb.nlm.nih.gov/record/ui?ui=D011596)   [**Neurobehavioral Manifestations [F01.700]**](https://meshb.nlm.nih.gov/record/ui?ui=D019954)   - [Anhedonia [F01.700.039]](https://meshb.nlm.nih.gov/record/ui?ui=D059445) - [Catatonia [F01.700.165]](https://meshb.nlm.nih.gov/record/ui?ui=D002389) - [Confusion [F01.700.250]](https://meshb.nlm.nih.gov/record/ui?ui=D003221) - [Consciousness Disorders [F01.700.315]](https://meshb.nlm.nih.gov/record/ui?ui=D003244) - [Lethargy [F01.700.470]](https://meshb.nlm.nih.gov/record/ui?ui=D053609) - [Memory Disorders [F01.700.625]](https://meshb.nlm.nih.gov/record/ui?ui=D008569) - [Intellectual Disability [F01.700.687]](https://meshb.nlm.nih.gov/record/ui?ui=D008607) - [Perceptual Disorders [F01.700.750]](https://meshb.nlm.nih.gov/record/ui?ui=D010468) - [Polydipsia, Psychogenic [F01.700.812]](https://meshb.nlm.nih.gov/record/ui?ui=D059607) - [Psychomotor Disorders [F01.700.875]](https://meshb.nlm.nih.gov/record/ui?ui=D011596) |
| **6** | **Psychological tests** | F04.711 | None | [Psychological Tests [F04.711]](https://meshb.nlm.nih.gov/record/ui?ui=D011581)   - - [Aptitude Tests [F04.711.141]](https://meshb.nlm.nih.gov/record/ui?ui=D001077)     - [Intelligence Tests [F04.711.141.493]](https://meshb.nlm.nih.gov/record/ui?ui=D007361)       - [Stanford-Binet Test [F04.711.141.493.225]](https://meshb.nlm.nih.gov/record/ui?ui=D013195)       - [Wechsler Scales [F04.711.141.493.822]](https://meshb.nlm.nih.gov/record/ui?ui=D014888)   - [Behavior Rating Scale [F04.711.271]](https://meshb.nlm.nih.gov/record/ui?ui=D000067290)   - [Ecological Momentary Assessment [F04.711.336]](https://meshb.nlm.nih.gov/record/ui?ui=D000072860)   - [Language Tests [F04.711.400]](https://meshb.nlm.nih.gov/record/ui?ui=D007807)   - [Neuropsychological Tests [F04.711.513]](https://meshb.nlm.nih.gov/record/ui?ui=D009483)     - [Bender-Gestalt Test [F04.711.513.180]](https://meshb.nlm.nih.gov/record/ui?ui=D001538)     - [Luria-Nebraska Neuropsychological Battery [F04.711.513.300]](https://meshb.nlm.nih.gov/record/ui?ui=D008182)     - [Mental Navigation Tests [F04.711.513.502]](https://meshb.nlm.nih.gov/record/ui?ui=D000070587)     - [Stroop Test [F04.711.513.703]](https://meshb.nlm.nih.gov/record/ui?ui=D057190)     - [Trail Making Test [F04.711.513.838]](https://meshb.nlm.nih.gov/record/ui?ui=D014145)   - [Personality Tests [F04.711.647]](https://meshb.nlm.nih.gov/record/ui?ui=D010556)     - [Bender-Gestalt Test [F04.711.647.138]](https://meshb.nlm.nih.gov/record/ui?ui=D001538)     - [Personality Inventory [F04.711.647.513]](https://meshb.nlm.nih.gov/record/ui?ui=D010555)       - [Cattell Personality Factor Questionnaire [F04.711.647.513.146]](https://meshb.nlm.nih.gov/record/ui?ui=D002416)       - [Manifest Anxiety Scale [F04.711.647.513.463]](https://meshb.nlm.nih.gov/record/ui?ui=D008347)       - [Millon Clinical Multiaxial Inventory [F04.711.647.513.535]](https://meshb.nlm.nih.gov/record/ui?ui=D058010)       - [MMPI [F04.711.647.513.607]](https://meshb.nlm.nih.gov/record/ui?ui=D008950)       - [Test Anxiety Scale [F04.711.647.513.886]](https://meshb.nlm.nih.gov/record/ui?ui=D013732)     - [Projective Techniques [F04.711.647.622]](https://meshb.nlm.nih.gov/record/ui?ui=D011386)     - [Semantic Differential [F04.711.647.745]](https://meshb.nlm.nih.gov/record/ui?ui=D012659)     - [Word Association Tests [F04.711.647.905]](https://meshb.nlm.nih.gov/record/ui?ui=D014936)   - [Psychometrics [F04.711.780]](https://meshb.nlm.nih.gov/record/ui?ui=D011594) |
| **7** | **Neuropsychology** | F04.096.795.600  H01.158.782.795.110 | None | NA |
| **8** | **Neuropsychiatry** | F04.096.544.504  H02.403.690.754 | None | NA |
| **9** | **Neurocognitive disorders** | F03.615 | Clerambault Syndrome  Delirium, Dementia, Amnestic, Cognitive Disorders  Kandinsky Syndrome  Mental Disorders, Organic  Nonpsychotic Organic Brain Syndrome  Organic Brain Syndrome, Nonpsychotic  Organic Mental Disorders  Organic Mental Disorders, Psychotic  Psychoses, Traumatic | [**Neurocognitive Disorders [F03.615]**](https://meshb.nlm.nih.gov/record/ui?ui=D019965)   - [Amnesia [F03.615.200]](https://meshb.nlm.nih.gov/record/ui?ui=D000647) - [Cognition Disorders [F03.615.250]](https://meshb.nlm.nih.gov/record/ui?ui=D003072) - [Consciousness Disorders [F03.615.300]](https://meshb.nlm.nih.gov/record/ui?ui=D003244) - [Delirium [F03.615.350]](https://meshb.nlm.nih.gov/record/ui?ui=D003693) - [Dementia [F03.615.400]](https://meshb.nlm.nih.gov/record/ui?ui=D003704) - [Dyslexia, Acquired [F03.615.700]](https://meshb.nlm.nih.gov/record/ui?ui=D004411) |
| **10** | **Psychomotor performance** | F02.808  G11.427.700  G11.561.660 | Perceptual Motor Performance  Sensory Motor Performance  Visual Motor Coordination | NA |
| **11** | **Cognitive neuroscience** | F04.096.628.255.500  H01.158.610.030 | None | NA |
| **12** | **Outcome assessment (health care)** | H01.770.644.145.431  N04.761.559.590  N05.715.360.575.575 | Assessment, Outcomes  Outcome Measures  Outcome Studies  Outcomes Assessment  Outcomes Research | [**Outcome Assessment (Health Care) [H01.770.644.145.431]**](https://meshb.nlm.nih.gov/record/ui?ui=D017063)   - [Failure to Rescue, Health Care [H01.770.644.145.431.500]](https://meshb.nlm.nih.gov/record/ui?ui=D000067248)   [**Outcome Assessment (Health Care) [N04.761.559.590]**](https://meshb.nlm.nih.gov/record/ui?ui=D017063)   - [Failure to Rescue, Health Care [N04.761.559.590.200]](https://meshb.nlm.nih.gov/record/ui?ui=D000067248) - [Patient Outcome Assessment [N04.761.559.590.399]](https://meshb.nlm.nih.gov/record/ui?ui=D063868)   - [Critical Care Outcomes [N04.761.559.590.399.250]](https://meshb.nlm.nih.gov/record/ui?ui=D000066891)   - [Lysholm Knee Score [N04.761.559.590.399.500]](https://meshb.nlm.nih.gov/record/ui?ui=D065466)   - [Minimal Clinically Important Difference [N04.761.559.590.399.750]](https://meshb.nlm.nih.gov/record/ui?ui=D000071081)   - [Patient Reported Outcome Measures [N04.761.559.590.399.875]](https://meshb.nlm.nih.gov/record/ui?ui=D000071066) - [Treatment Outcome [N04.761.559.590.800]](https://meshb.nlm.nih.gov/record/ui?ui=D016896) - [Watchful Waiting [N04.761.559.590.900]](https://meshb.nlm.nih.gov/record/ui?ui=D057832)   [**Outcome Assessment (Health Care) [N05.715.360.575.575]**](https://meshb.nlm.nih.gov/record/ui?ui=D017063)   - [Critical Care Outcomes [N05.715.360.575.575.200]](https://meshb.nlm.nih.gov/record/ui?ui=D000066891) - [Failure to Rescue, Health Care [N05.715.360.575.575.300]](https://meshb.nlm.nih.gov/record/ui?ui=D000067248) - [Patient Outcome Assessment [N05.715.360.575.575.399]](https://meshb.nlm.nih.gov/record/ui?ui=D063868)   - [Critical Care Outcomes [N05.715.360.575.575.399.250]](https://meshb.nlm.nih.gov/record/ui?ui=D000066891)   - [Lysholm Knee Score [N05.715.360.575.575.399.500]](https://meshb.nlm.nih.gov/record/ui?ui=D065466)   - [Minimal Clinically Important Difference [N05.715.360.575.575.399.750]](https://meshb.nlm.nih.gov/record/ui?ui=D000071081)   - [Patient Reported Outcome Measures [N05.715.360.575.575.399.875]](https://meshb.nlm.nih.gov/record/ui?ui=D000071066) - [Treatment Outcome [N05.715.360.575.575.800]](https://meshb.nlm.nih.gov/record/ui?ui=D016896) |
| **13** | **Meningioma** | C04.557.580.520  C04.557.645.520  C04.588.614.250.580.500  C10.551.240.500.500 | Angioblastic Meningioma  Angiomatous Meningioma  Benign Meningioma  Cerebral Convexity Meningioma  Clear Cell Meningioma  Fibrous Meningioma  Hemangioblastic Meningioma  Hemangiopericytic Meningioma  Intracranial Meningioma  Intraorbital Meningioma  Intraventricular Meningioma  Malignant Meningioma  Meningiomas, Multiple  Meningiomatosis  Meningotheliomatous Meningioma  Microcystic Meningioma  Olfactory Groove Meningioma  Papillary Meningioma  Parasagittal Meningioma  Posterior Fossa Meningioma  Psammomatous Meningioma  Secretory Meningioma  Sphenoid Wing Meningioma  Spinal Meningioma  Transitional Meningioma  Xanthomatous Meningioma | NA |
| **14** | **Meningeal neoplasms** | C04.588.614.250.580  C10.551.240.500 | Benign Meningeal Neoplasms  Intracranial Meningeal Neoplasms  Leptomeningeal Neoplasms  Malignant Meningeal Neoplasms  Meningeal Cancer  Meningeal Neoplasms, Benign  Meningeal Neoplasms, Intracranial  Meningeal Neoplasms, Malignant  Meningeal Tumors  Neoplasms, Leptomeningeal  Neoplasms, Meningeal  Spinal Meningeal Neoplasms | [**Meningeal Neoplasms [C10.551.240.500]**](https://meshb.nlm.nih.gov/record/ui?ui=D008577)   - [Meningeal Carcinomatosis [C10.551.240.500.150]](https://meshb.nlm.nih.gov/record/ui?ui=D055756) - [Meningioma [C10.551.240.500.500]](https://meshb.nlm.nih.gov/record/ui?ui=D008579)   [**Meningeal Neoplasms [C04.588.614.250.580]**](https://meshb.nlm.nih.gov/record/ui?ui=D008577)   - [Meningeal Carcinomatosis [C04.588.614.250.580.150]](https://meshb.nlm.nih.gov/record/ui?ui=D055756) - [Meningioma [C04.588.614.250.580.500]](https://meshb.nlm.nih.gov/record/ui?ui=D008579) |
| **15** | **Pituitary neoplasms** | C04.588.322.609  C04.588.614.250.195.885.500.600  C10.228.140.211.885.500.600  C10.228.140.617.477.600  C10.228.140.617.738.675  C10.551.240.250.700.500.500  C19.344.609  C19.700.734 | Cancer of Pituitary  Cancer of the Pituitary  Pituitary Adenoma  Pituitary Cancer  Pituitary Carcinoma  Pituitary Tumors | [**Pituitary Neoplasms [C04.588.322.609]**](https://meshb.nlm.nih.gov/record/ui?ui=D010911)   - [ACTH-Secreting Pituitary Adenoma [C04.588.322.609.145]](https://meshb.nlm.nih.gov/record/ui?ui=D049913) - [Growth Hormone-Secreting Pituitary Adenoma [C04.588.322.609.292]](https://meshb.nlm.nih.gov/record/ui?ui=D049912) - [Prolactinoma [C04.588.322.609.792]](https://meshb.nlm.nih.gov/record/ui?ui=D015175)   [**Pituitary Neoplasms [C04.588.322.609]**](https://meshb.nlm.nih.gov/record/ui?ui=D010911)   - [ACTH-Secreting Pituitary Adenoma [C04.588.322.609.145]](https://meshb.nlm.nih.gov/record/ui?ui=D049913) - [Growth Hormone-Secreting Pituitary Adenoma [C04.588.322.609.292]](https://meshb.nlm.nih.gov/record/ui?ui=D049912) - [Prolactinoma [C04.588.322.609.792]](https://meshb.nlm.nih.gov/record/ui?ui=D015175)   [**Pituitary Neoplasms [C19.344.609]**](https://meshb.nlm.nih.gov/record/ui?ui=D010911)   - [ACTH-Secreting Pituitary Adenoma [C19.344.609.145]](https://meshb.nlm.nih.gov/record/ui?ui=D049913) - [Growth Hormone-Secreting Pituitary Adenoma [C19.344.609.292]](https://meshb.nlm.nih.gov/record/ui?ui=D049912) - [Prolactinoma [C19.344.609.792]](https://meshb.nlm.nih.gov/record/ui?ui=D015175)   [**Pituitary Neoplasms [C19.700.734]**](https://meshb.nlm.nih.gov/record/ui?ui=D010911)   - [ACTH-Secreting Pituitary Adenoma [C19.700.734.145]](https://meshb.nlm.nih.gov/record/ui?ui=D049913) - [Growth Hormone-Secreting Pituitary Adenoma [C19.700.734.292]](https://meshb.nlm.nih.gov/record/ui?ui=D049912) - [Prolactinoma [C19.700.734.792]](https://meshb.nlm.nih.gov/record/ui?ui=D015175) |
| **16** | **Skull base** | A01.456.830  A02.835.232.781.750 | Base of Skull  Basicranium  Basis cranii  Cranial Base | [**Skull Base [A01.456.830]**](https://meshb.nlm.nih.gov/record/ui?ui=D019291)   - [Cranial Fossa, Anterior [A01.456.830.150]](https://meshb.nlm.nih.gov/record/ui?ui=D035262) - [Cranial Fossa, Middle [A01.456.830.165]](https://meshb.nlm.nih.gov/record/ui?ui=D035301) - [Cranial Fossa, Posterior [A01.456.830.200]](https://meshb.nlm.nih.gov/record/ui?ui=D003388) - [Infratemporal Fossa [A01.456.830.600]](https://meshb.nlm.nih.gov/record/ui?ui=D000080884) - [Parapharyngeal Space [A01.456.830.800]](https://meshb.nlm.nih.gov/record/ui?ui=D000080886)   [**Skull Base [A02.835.232.781.750]**](https://meshb.nlm.nih.gov/record/ui?ui=D019291)   - [Cranial Fossa, Anterior [A02.835.232.781.750.150]](https://meshb.nlm.nih.gov/record/ui?ui=D035262) - [Cranial Fossa, Middle [A02.835.232.781.750.165]](https://meshb.nlm.nih.gov/record/ui?ui=D035301) - [Cranial Fossa, Posterior [A02.835.232.781.750.400]](https://meshb.nlm.nih.gov/record/ui?ui=D003388) - [Infratemporal Fossa [A02.835.232.781.750.700]](https://meshb.nlm.nih.gov/record/ui?ui=D000080884) - [Parapharyngeal Space [A02.835.232.781.750.850]](https://meshb.nlm.nih.gov/record/ui?ui=D000080886) |
| **17** | **Skull neoplasms** | C04.588.149.721  C05.116.231.754 | Neoplasms, Skull | [**Skull Neoplasms [C05.116.231.754]**](https://meshb.nlm.nih.gov/record/ui?ui=D012888)   - [Jaw Neoplasms [C05.116.231.754.450]](https://meshb.nlm.nih.gov/record/ui?ui=D007573) - [Nose Neoplasms [C05.116.231.754.600]](https://meshb.nlm.nih.gov/record/ui?ui=D009669) - [Orbital Neoplasms [C05.116.231.754.659]](https://meshb.nlm.nih.gov/record/ui?ui=D009918) - [Skull Base Neoplasms [C05.116.231.754.829]](https://meshb.nlm.nih.gov/record/ui?ui=D019292)   [**Skull Neoplasms [C04.588.149.721]**](https://meshb.nlm.nih.gov/record/ui?ui=D012888)   - [Jaw Neoplasms [C04.588.149.721.450]](https://meshb.nlm.nih.gov/record/ui?ui=D007573) - [Nose Neoplasms [C04.588.149.721.600]](https://meshb.nlm.nih.gov/record/ui?ui=D009669) - [Orbital Neoplasms [C04.588.149.721.656]](https://meshb.nlm.nih.gov/record/ui?ui=D009918) - [Skull Base Neoplasms [C04.588.149.721.828]](https://meshb.nlm.nih.gov/record/ui?ui=D019292) |
| **18** | **Skull base neoplasms** | C04.588.149.721.828  C05.116.231.754.829 | Neoplasms, Skull Base | NA |

NA = no further subdivisons made (end of a tree)
