# Supplementary material for: Influences on cognitive outcomes in adult patients with gliomas: A systematic review
Source: Front Oncol. 2022 Aug 5;12:943600. doi: 10.3389/fonc.2022.943600 (PMC9407441; doi:10.3389/fonc.2022.943600)
Supplement: Supplementary file 2 [file Table_2.docx]

| **Search themes** | **Search term(s)** |
| --- | --- |
| Brain tumour | Brain neoplasms.mp. or exp Brain Neoplasms/ |
|  | exp Astrocytoma/ or Neoplasms, Neuroepithelial.mp. or exp Medulloblastoma/ or  exp Cerebellar Neoplasms/ or exp Neoplasms, Neuroepithelial/ or exp Neuroectodermal Tumors, Primitive, Peripheral/ or exp Brain Neoplasms/ |
|  | Neoplasm Metastasis.mp. or exp Neoplasm Metastasis/ |
|  | (Brain tum* or cerebral tum* or glio* or brain metastas* or cerebral metastas* or supratentorial tum* or supratentorial metastas* or infratentorial tum* or infratentorial metastas* or brain cancer or cerebral cancer or brain malignan* or cerebral malignan* or primary brain tum* or primary brain cancer or brain neoplas* or cerebral neoplas* or suptratentorial neoplas* or infratentorial neoplas*).mp. |
|  | Meningioma/ or intracranial tumor.mp. or Pituitary Neoplasms/ or Meningeal  Neoplasms/ |
|  | Skull Base/ or Skull Neoplasms/ or Skull Base Neoplasms/ or skull base tumor.mp. |
|  |  |
| Cognition | Mental processes.mp. or exp Mental Processes/ |
|  | Neurobehavioral manifestation.mp. or exp Neurobehavioral Manifestations/ |
|  | Psychological tests.mp. or exp Psychological Tests/ |
|  | Neuropsychology.mp. or exp Neuropsychology/ |
|  | Neuropsychiatry.mp. or exp Neuropsychiatry/ |
|  | Neurocognitive disorders.mp. or exp Neurocognitive Disorders/ |
|  | Psychomotor performance.mp. or exp Psychomotor Performance/ |
|  | Cognitive neuroscience.mp. or exp Cognitive Neuroscience/ |
|  | (Cogniti* or psycholog* or neuropsycholog* or memory or executive functio* or spatia* or learnin* or percept* or attentio* or intell* or neuropsychiat* or psychologica* or behavio* or neurocogniti*).mp. |
|  |  |
| Outcome/ recovery/ plasticity | Outcome assessment health care.mp. or exp “Outcome Assessment (Health Care)”/ |
|  | (Recover* or outcom* or plastic*).mp. |
|  |  |
