# Supplementary material for: Influences on cognitive outcomes in adult patients with gliomas: A systematic review
Source: Front Oncol. 2022 Aug 5;12:943600. doi: 10.3389/fonc.2022.943600 (PMC9407441; doi:10.3389/fonc.2022.943600)
Supplement: Supplementary file 3 [file Table_3.docx]

| **References** | **Study population** | **Country** | **Year** | **Study type** | **n** | **Test timing** | **Treatment(s) received** | **Tests used/domains tested** | **Main findings** |
| --- | --- | --- | --- | --- | --- | --- | --- | --- | --- |
| (Allen et al. 2020) | WHO grade II brain tumour (n=5), WHO grade III brain tumour (n=25), WHO grade IV brain tumour (n=10) | USA | 2020 | Prospective cohort | 40 | Range of 1.3 to 25 years (median: 6.7 years) from original diagnosis | Surgery (n=34), chemotherapy (n=39), radiotherapy (n=35) | Screening MMSE followed by TMT parts A and B, HVLT-R, Multilingual Aphasia Examination–COWAT, and patient-reported Everyday Cognitive Scale | This study aimed to evaluate the association between performance-based neuro-cognitive and patient-reported cognitive function tests in primary brain tumour patients with an MMSE score of 24 or greater. The results indicated that age, time since diagnosis, and tumour- or treatment-specific variables were not associated with neurocognitive or patient-reported cognitive function. Those that reported worse cognitive impairment also reported greater severity of primary brain tumour-specific and depressive symptoms. |
| (Altieri et al. 2019) | DNET (4%), WHO grade II astrocytomas (17%), WHO grade II oligodendroglioma (27%), anaplastic astrocytoma (13%), glioblastoma (39%) | Italy | 2019 | Retrospective | 23 | Before surgery, 5 days and 1 month after surgery | Surgery (all) | Writing, motor speech, comprehension, expression, reading, pragmatics, attention, memory, problem solving, and visuoperceptive functions evaluated using the NOMS | This study retrospectively reviewed outcomes in a series of 23 patients undergoing awake craniotomy for low- and high-grade brain tumours. The study found that memory was the only cognitive domain that deteriorated after surgery and had not completely recovered at one month post-operatively. |
| (Altshuler et al. 2019) | WHO grade II glioma (n=19), WHO grade III glioma (n=31), WHO grade IV glioma (n=78) | USA | 2019 | Retrospective | 128 | Before surgery and 3-months postoperatively | Surgery (all), radiotherapy (n=119), chemotherapy (n=120) | RBANS, TMT, Stroop Test | This study evaluated the relationship between genetic polymorphisms, neurocognitive function, and ability to return to work in patients with glioma at the point of diagnosis and at 3 months. The genes studied were BDNF, DRD2, and COMT. The study found that patients with so-called higher-performing alleles had better scores on neurocognitive testing with the Repeatable Battery for the Assessment of Neuropsychological Status and Stroop Test, but not the TMT. |
| (Anders et al. 2021) | WHO grade II glioma (n=11), WHO grade III glioma (n=10), WHO grade IV glioma (n=60) | Denmark | 2021 | Prospective cohort | 81 | During the first week of chemoradiation, following surgery (mean of 40.3 and 39.8 days from surgery in the right- and left-hemisphere tumours, respectively) | Surgery (n=75), radiotherapy (n=72), chemotherapy (n=63), other (n=6) | EORTC-QLQ-C30, QLQ-BN20, and tests of functional performance | This study evaluated the role of tumour laterality in health-related quality of life, symptomatology, and functional performance. The study found no group differences in health-related quality of life, but patients with left-hemisphere glioma had more communication deficits and those with right-hemisphere glioma had inferior process skills in relation to performing everyday tasks such as cooking and ironing. |
| (Anderson et al. 1999) | High grade gliomas (n=16), LGG (n=14), meningioma (n=10) | UK | 1999 | Prospective cohort | 40 | Mean of 36 weeks following surgery (range 15-67 weeks) | Biopsy (n=7), surgical resection (n=33), radiotherapy (n=25), chemotherapy (n=9) | Not specified, but reported to cover the domains of memory (verbal and non-verbal), concentration, language function, visuo-spatial abilities, and frontal or executive function. | A trend for neuropsychological functioning to be negatively correlated with psychological distress; only self-reported depression was significantly correlated with cognitive impairment. |
| (Antonsson, Jakola, et al. 2018) | LGG (n=23), HGG (n=9) | Sweden | 2018 | Prospective cohort | 32 | Pre-operatively, early post-operatively, and 3 months post-operatively. | Previous biopsy (n=3), resection (n=6), radiotherapy (n=5), chemotherapy (n=3) | Aphasia (A-ning), high-level language (Bedömning av subtila språkstörningar, assessment of subtle language disorders), morpho-logical ability (sentence analysis, morphological completion), lexical retrieval (BNT, word fluency, FAS [letter fluency], animals [semantic fluency], verbs [semantic fluency]), language comprehension (Token Test) | This study investigated language outcomes in patients with presumed LGGs (although some of these patients were later confirmed to have HGGs). Prior to surgery, performance in lexical retrieval tasks (BNT) was worse when compared to a reference group. At early post-operative assessment, a decline in all language tests was observed, but at 3 months a decline was only found in a single test of lexical retrieval (animals). Patients with tumours in language-dominant areas had the highest proportion of language impairment at all time points. |
| (Antonsson, Johansson, et al. 2018) | LGG (n=20), healthy controls (n=31) | Sweden | 2018 | Prospective cohort | 51 | Pre-operatively and at 3 months post-operatively | Surgery (all) | BNT, FAS, Semantic fluency (animals and verbs), Picture-elicited narrative task, Copy Task | Writing fluency affected before and after surgery, and typing speed is an important factor behind the pre-operative differences. A lexical deficit may explain a decline in overall productivity and increase in pauses before words that was observed after surgery; this is supported by the finding that oral lexical-retrieval scores correlated strongly with writing fluency. |
| (Arbula et al. 2020) | HGG (n=24), LGG (n=9), meningioma (n=14), metastases (n=7) | Italy and USA | 2020 | Prospective cohort | 54 | On average 4 days before and 5.6 days after surgery. | Surgery (all), no other treatments specified | General cognitive functioning and intellectual ability (MMSE and the Italian version of NART - TIB), verbal and spatial short-term memory (Digit Span, Corsi), visuo-spatial abilities (TMT-A), and phonemic fluency (letters C, P, and S). | This study evaluated the role of lesion location in cognitive outcomes in patients with a range of brain tumours. It found that lesions in the left lateral prefrontal areas, including the inferior frontal junction, were associated with the most severe cognitive deficits, over and above tumour histology. |
| (Armstrong et al. 2000) | Low-grade tumours (including gliomas, pituitary tumours, pineal tumour, meningioma, ependymomas, and plasmacytoma) | USA | 2000 | Prospective cohort | 20 | After surgery, before radiotherapy, and 3, 6, and 12 months after radiotherapy. | Radiotherapy (all) after biopsy or surgical resection (n not specified) | Tests of motor function (Praxis, Finger Tapping Test), attention (selective visual attention, auditory selective attention, language (Sentence Repetition Test, word fluency, category fluency), verbal short-term memory (phonologic working memory span, semantic working memory span), short-term memory (visuospatial working memory span, immediate simple figure recall, Rey-Osterrieth Complex Figure immediate recall), cognitive processing speed (PASAT, Symbol Digit Modalities Test), verbal memory (Digit Span Test [Forward], Word Span Test, RAVLT [Learning (total for Trials 1 to 5), Post-Interference Retrieval (T6), Retention (T6/T5), Delayed Recall (T7), Retention after Delay (T7/T6), word recognition], visuospatial perception (Road Map Test, Visual Pursuits Test [number of items completed, percent accurate responses], ROCFT [copy of figure]), visual memory (ROCFT delayed recall, Biber Figure Learning Test [Learning (total for Trials 1 to 5)], Retrieval after interference, Retrieval after delay, Biber Word Recognition), and conceptual set shifting. | Nonverbal memory was normal at baseline, after which it reduced and then rebounded. Visual memory was impaired at baseline and recovered up to 1 year after radiotherapy. No other changes over time were found in other neurocognitive measures or in fatigue or mood. |
| (Armstrong et al. 2002) | Low-grade supratentorial tumours (gliomas, pituitary and pineal tumours, and non-invasive meningiomas) | USA | 2002 | Prospective cohort | 26 | Six weeks after surgery, immediately before radiotherapy, and annually for 6 years | Radiotherapy (all) after biopsy, surgical resection, or no surgical management (n not specified) | Tests of motor function (Praxis, Finger Tapping Test), attention (selective visual attention [Bells Test], Auditory Selective Attention Test, sustained visual attention [Continuous Performance Test]), language (Sentence Repetition Test, Controlled Oral Word Association Test, Animal Naming Test [category fluency]), cognitive processing speed (Paced Auditory Serial Addition Test [PASAT], Symbol Digit Modalities Test [oral version]), verbal memory (Digit Span Test [Forward], Word Span Test, Rey Auditory Verbal Learning Test [Learning (total for Trials 1 to 5), Post-Interference Retrieval (T6), Retention (T6/T5), Delayed Recall (T7), Retention after Delay (T7/T6), Total Recognition Hits, Total Recognition False Alarm Responses]), visuospatial perception (Road Map Test, Visual Pursuits Test [number of items completed, percent accurate responses], ROCFT [copy of figure]), visual memory (Visual Memory Span Test [Forward], Revised Benton Visual Retention Test [total correct], ROCFT [immediate recall, delayed recall], Biber Figure Learning Test [Learning (total for Trials 1 to 5), Post-Interference Retrieval (T6), Retention (T6/T5), Delayed Recall (T7), Retention after Delay (T7/T6), Total Recognition Hits, Total Recognition False Alarm Responses]), and conceptual set shifting (Wisconsin Card Sorting Test [number of categories achieved, perseverative errors]). | Approximately half of the patients demonstrated cognitive decline and treatment-related hyperintensities. However, there was no evidence of a general cognitive decline or progression of T2-weighted MRI white matter hyperintensities after 3 years. |
| (Aydinlar et al. 2020) | GBM (n=2), astrocytoma (WHO grade not specified; n=5), oligodendroglioma (WHO grade not specified; n=2), glioma (n=3) | Turkey | 2020 | Prospective cohort | 12 | The day before surgery, 2 days after surgery, and 3 months after surgery | Surgery (all), no other treatments specified | MoCA - Turkish version, picture description task using the "Cookie Theft" picture from the BDAE test | This study used electrophysiological mapping to identify an association between the preservation of long latency response evoked by electrophysiological mapping of the inferior frontal gyrus and postoperative speech function after tumour removal. The findings showed that electrical stimulation was able to elicit a long latency response in 75% of patients, and 33.3% of patients showed a temporary impairment in fluent speech postoperatively that recovered fully within 3 months. |
| (Barzilai et al. 2018) | LGG | Israel | 2019 | Retrospective | 49 | Pre-operatively and 3-28 (mean 6.06 months) postoperatively | Surgery (all) | Attention and working memory (WAIS-III Hebrew Digit Span, WMS-III Spatial Span), visuomotor organization (pre-op: Rey complex figure test - copy, post-op: Taylor alternative figure test - copy), memory and learning (pre-op: Rey complex figure test - delayed memory, post-op: Taylor alternative figure test - delayed memory, RAVLT), language (Hebrew naming test, COWAT: semantic fluency), executive function (COWAT: phonemic fluency, Stroop test-interference score), and IQ prediction (WAIS-III Hebrew similarities). | Resection of LGG found to significantly improve memory and executive function (dominant hemisphere tumours) and memory alone (non-dominant hemisphere tumours). Positive staining for GFAP and p53 positivity were found to be associated with improved cognitive outcomes. No association between cognitive outcomes and pre-operative tumour volume, residual tumour volume, extent of resection, or IDH1 status. |
| (Berger et al. 2019) | LGG | Israel | 2019 | Retrospective | 82 | Before surgery and 3-months postoperatively | Surgery (all), radiotherapy (8% in no infarct group, 11% in infarct group) | Memory (verbal and nonverbal), executive function and attention (Go-NoGo, Stroop), vernal function, visual spatial processing, problem solving) | This study used a battery of computerised cognitive tests to identify the influence of stroke following surgery for LGG on cognitive outcomes, as well as other variables. In the 33 participants that underwent neurocognitive testing before and after surgery, the study found that neurocognitive functions between those that did and did not develop an infarct were generally stable, but those that developed a stroke experienced a decline in verbal rhyming ability. |
| (Bette et al. 2020) | GBM (n=30), WHO grade III glioma (n=8), WHO grade II glioma (n=15), pineocytoma (n=8), pilocytic astrocytoma (n=3), ganglioglioma (n=2), DNET (n=2), neurocytoma (n=2), subependymoma (n=1) | Germany | 2020 | Prospective cohort | 71 | Mean of 5 days (range 0-56 days) before surgery and 8 days (range: 3-53) after surgery | Surgery (all), postoperative chemotherapy (n=32), postoperative radiotherapy (n=31) | MMSE, tests of attention (Test Battery of Attentional Performance, TMT-A), memory (Wechsler Memory Test, Verbal Learning and Memory Test, ROCFT), executive function (TMT-B, Fegensburg Word Fluency Test, Stroop Test), as well as functional independence (KPS) and quality of life (SF-36). | This study evaluated cognitive function in a range of tumours before and after surgery. Cognitive outcomes varied by tumour grade. No cognitive deterioration was observed in patients with WHO grade I tumours following surgery. Patients with WHO grade II tumours had significantly worse results in executive function testing, whereas those with WHO grade IV tumours showed deterioration in the attention tests. Other risk factors for postoperative deterioration of cognitive function included low KPS scores, postoperative radiotherapy, and location of the tumour in the temporal lobe. |
| (Blonski et al. 2012) | WHO II diffuse glioma | France | 2012 | Retrospective | 10 | A median of 17.9 months (range: 3.1-70.1 months) after surgery | Maximal surgical resection (all), chemotherapy (all) | Global efficiency (MMSE), pre-morbid intelligence (French National Adult Reading Test), handedness (Edinburgh Inventory), information processing and psychomotor speed (digit-symbol substitution test - subtest WAIS-III, TMT-A), attention (Bell's test, Rey figure - copy), episodic memory (verbal - Grober and Buschke; non-verbal: Rey Figure), working memory (verbal - Digit Span, letter-digit sequencing - subtest WAIS-III; non-verbal - visuospatial span), language (Naming Test, Literal and categorical word fluency), visuospatial abilities (Visual Object Space Perception - incomplete letters and cube analysis subtests, PEGV, Naming Test, Rey Figure, Bell's test), and executive functions (verbal abstraction ability - similarities [WAIS subtest]; deduction rules - Brixton test; flexibility - TMT-A and -B; inhibition - Stroop colour-word test; verbal initiation - literal and categorical fluency). | Of the 10 patients, 3 had no neuropsychological deficits, and 7 had slight impairment in verbal episodic memory and executive functions. Global quality of life was roughly preserved (assessed using the EORTC QLQ C30 + BN20). Cognitive deficits were seen to relate mainly to tumour location; executive deficits were mainly seen in patients with frontal tumours, and hippocampal profiles of memory impairment were related to temporal tumours. Interestingly, in one patient, significant executive deficits were seen despite the tumour being mesio-temporal in origin. |
| (Boone et al. 2016) | WHO I-III gliomas | France | 2016 | Prospective | 27 | Median 3 years after treatment | Surgery (n=25), radiotherapy (n=19), chemotherapy (n=6) | Global cognitive efficiency (MMSE, progressive matrices 47, Goldberg scale), language (Token Test, BNT), visuospatial abilities (Albert cancellation test, Rey figure copy), memory (Digit and spatial span, Free and Cued Selective Reminding Test, Door Test), action speed and executive functions (Digit Symbol Substitution Subtest, Stroop, TMT, Verbal Fluency Test, Modified Card Sorting Test, Six Elements Test, Strategy Index of Free and Cued Selective Reminding Test, Behavioral Dysexecutive Syndrome Inventory). | Cognitive impairment was seen in 51.9% of survivors, with effects on action speed (38.5%), cognitive (33%), behavioural (21.7%) executive functions, oral expression (29.6%), episodic memory (29.6%), and visuoconstructive abilities (19.2%). The largest effect sizes were observed for the Digit Symbol Substitution test, global hypoactivity, free recall, Stroop time, BNT, TMT-B, verbal fluency and the ROCFT. Overall neuropsychological score was not correlated with tumour characteristics or treatment, including: WHO grade, delay of assessment following surgery, delay of assessment following complementary treatment, NIHSS motor score, surgical treatment, chemotherapy, radiotherapy, and seizure history. Poor functional outcome was significantly associated with overall neuropsychological score. |
| (Borde et al. 2021) | Multiple types, including intrinsic and extra-axial tumours (although exact proportions not specified) | India | 2021 | Prospective cohort | 50 | Before surgery, 1 week post-operatively, and 3 months post-operatively | Surgery (all), no other treatments specified | FAB (assesses conceptualisation, mental flexibility, programming, conflicting instructions, inhibitory control, and environmental autonomy), MMSE, Verbal Learning and Memory Test | This study aimed to correlate pre- and post-operative cognitive dysfunction in patients with frontal tumours. The study found that only 12 of 50 participants has 'good' FAB scores before surgery. After surgery, 26 patients had a good FAB score, and this increased to 44 of the 50 participants at 3 months. |
| (Braun et al. 2006) | HGG, LGG | Germany | 2006 | Prospective cohort | 14 | Pre- and post-operatively (exact timing not specified) | Surgery (all), radiotherapy (n=3), chemotherapy (n=4), and combined radiotherapy/chemotherapy (n=3) | General working memory capacity (MMSE, Performance Test, Clock Drawing, TMT, Block Span Forward, Block Span Backward, Visuospatial Memory Test), visuospatial short-term memory (Verbal Fluency, WMS - Revised I and II), verbal memory (Alertness with Signal, Alertness without Signal, Dual Task, Go/No Go), complex items combining verbal and visuospatial memory tasks (WCST, Odd Man Out) | Patients underwent fMRI to detect the motor cortex and Broca's area, with this imaging integrated into neuronavigation to facilitate surgery. In all patients the general level of working memory was preserved following surgery. Visuo-spatial performance either remained unchanged or deteriorated slightly, and alertness worsened slightly. However, performance with verbal test items improved after surgery. |
| (Breen et al. 2020) | LGG | USA | 2020 | RCT | 203 | Baseline and then every 4 months for years 1-2, every 6 months for years 3-5, and annually thereafter. | Biopsy (n=102), subtotal resection (n=71), gross total resection (n=30) | MMSE | This RCT compared two different doses of radiation therapy (50.4 Gy in 28 fractions versus 64.8 Gy in 36 fractions) following surgery. The outcome measures included oncologic and cognitive outcomes. The study found that, in patients with a normal baseline MMSE, only one patient (5%) had a clinically significant decrease in MMSE from the prior timepoint, and the remainder were stable. No participants had a reduction in MMSE at 10, 12, or 15 years. Cognitive function appeared to be stable after radiation. There was no significant difference in the long-term changes in MMSE between the two radiation dose groups. |
| (Brennum et al. 2018) | WHO grade II gliomas (79%), WHO grade gliomas III (21%) | Denmark | 2018 | Retrospective | 92 | Pre-operatively and 6 and 12 months post-operatively. | Surgery (all), no other treatments specified | Not specified, but reported to cover the domains of executive function, language, memory, attention, and mood) | This retrospective study evaluated whether pre- and intra-operative factors were linked to an increased risk of postoperative deficits in 92 patients that underwent awake craniotomy with cortical and subcortical mapping. The study found that a decrease in cognitive function following surgery was common in the first few days, with a significant improvement observed at 6 months and a further improvement at 12 months. |
| (Brown et al. 2003) | LGG | USA | 2003 | Analysis of data collected as part of an RCT | 187 | Baseline (study entry) and after the completion of protocol therapy (every 4 months for 2 years, every 6 months for 3 years, and yearly until year 15) | Surgery (all), low-dose (50.4 Gy) radiotherapy (n=95), conventional (64.8 Gy) radiotherapy (n=83) | MMSE | This study evaluated the neurocognitive effects of cranial radiotherapy at two different doses, using data collected from an RCT. The study found that most patients maintained a stable neurocognitive status after focal radiotherapy. Patients with an abnormal baseline MMSE score were more likely to have improved cognitive abilities than deterioration after radiotherapy. Only a small proportion of patients experienced cognitive decline following radiotherapy. There was no relationship found between changes in cognitive function and baseline variables such as radiation dose, conformal versus conventional radiotherapy, number of radiation fields, age, sex, tumour size, Neurologic Function Score, seizures, or seizure medications. |
| (Bunevičius et al. 2017) | Primary brain tumours (meningioma, high grade glioma, low grade glioma, pituitary adenoma, acoustic neuroma, other diagnosis) | Lithuania, Italy, and USA | 2016 | Prospective cohort | 230 | Pre-operative only | Gross total resection (n=201), subtotal resection (n=26), biopsy (n=3), chemotherapy (n=22), radiotherapy (n=61) | MMSE | Low tri-iodothyronine levels were common in the study population (74%) and associated with lower MMSE scores. |
| (Butterbrod et al. 2021) | Glioma (n=263), meningioma (n=242) | The Netherlands | 2021 | Prospective cohort | 505 | One day before, 3 months, and 12 months after surgery | Surgical debulking (all), adjuvant treatment - radiotherapy and/or chemotherapy (39% in e4 carrier group, 42% in non-carrier group) | Dutch translation of the CNS Vital Signs computerised test battery, comprising of: Verbal Memory Test, Visual Memory test (VIM), SDC, SAT, CPT, Stroop test I and Stroop test III | This study investigated the pre-surgical cognitive performance and postsurgical changes in patients with glioma and meningioma with and without the APOE e4 allele. It found no difference in pre-treatment performance between carriers and non-carriers, and no significant main effect of e4 carrier status or interaction between time and carrier status on any of the tests in the entire sample or in the sample receiving adjuvant treatment. The authors concluded that the study found no evidence of increased vulnerability to pretreatment cognitive dysfunction or cognitive decline within 1 year after surgery in glioma and meningioma patients carrying the APOE e4 allele. |
| (Campanella et al. 2015) | High-grade glioma (n=29), low-grade glioma (n=21), meningioma (n=16) | Italy, UK | 2015 | Prospective cohort | 66 | Pre-operatively and post-operatively (usually within the week after surgery) | Surgery | Language (Token Test), executive functions (FAB), attention (Visual Search, Stars Cancellation), perception (Birmingham Object Recognition Battery subtest 8, BFRT), visuospatial skills (Rey Figure - immediate), long-term memory (Rey Figure - delayed, Narrative Memory), short-term memory (Digit Span Forward and Backward, Corsi Spatial Span). | HGG patients were largely already impaired in the more perceptual tasks before surgery and surgery did not influence their performance. LGG patients, however, who were unimpaired before surgery, showed a significant deficit in perceptual tasks immediately after surgery that largely recovered at the point of a repeat assessment approximately 4 months after surgery. To contrast, meningioma patients were largely unimpaired in all tasks. |
| (Campanella et al. 2017) | Low-grade glioma | Italy, Sweden, and UK | 2017 | Prospective | 50 | Median of 40 (range 12-181) months following surgery | Surgical resection (all), chemotherapy (n=5), radiotherapy (n=5), chemotherapy and radiotherapy (n=2) | Logical reasoning (Raven Coloured Matrices), picture naming (Category-Specific Picture Naming), verbal comprehension (Token Test), verbal fluency (Letter Fluency), auditory repetition/writing/reading (AAT), sustained and divided attention (Visual Search), attentional switching (TMT), executive functions (FAB), short-term memory (Digit Span Forward and Backward, Corsi Spatial Span), long-term memory (Short Story Recall, ROCFT - delayed recall), visuospatial skills (ROCFT - immediate copy, Behavioural Inattention Test). | The cognitive profile of the patients at 3.5 years following surgery was similar to the healthy population in all domains except short-term memory. Short-term memory was not influenced by variables linked to surgery, such as tumour laterality or lobe affected, or tumour volume, or EOR; it was, however, linked to the number of antiepileptic medications prescribed to the patient. |
| (Campanella et al. 2018) | HGG (n=130), LGG (n=76), meningioma (n=54) | Italy, Sweden, UK | 2018 | Retrospective | 260 | Day before surgery and 5-7 days after surgery | Surgery (all) | Verbal short-term memory (Digit Span Forward), spatial short-term memory (Corsi Spatial Span), verbal long-term memory (Narrative Memory), visuospatial long-term memory (Delayed Recall of ROCFT) | This study attempted to map anatomical 'hotspots' for short- and long-term memory functions. It found that patients with HGG had impaired memory prior to surgery, but patients with meningioma were largely unimpaired. Patients with LGG, although unimpaired before surgery, showed a significant drop in performance immediately after, with a good recovery within a few months. Verbal memory tasks identified specific anatomical hotspots that can be considered 'eloquent' for verbal memory functions, unlike visuospatial tasks. |
| (Campanella et al. 2021) | LGG (n=32), HGG (n=46), meningioma (n=22) | Italy | 2021 | Prospective cohort | 100 | An average of 3.01 (±5.32) days before surgery and an average of 7.29 (±3.84) days after surgery | Surgery (all) | Picture Naming, Token Test, Verbal Fluency, Auditory Repetition, Reading, Writing | This study explored whether the concept of cognitive reserve can be applied to cognitive functions in patients with brain tumours. Different proxies for cognitive reserve (education level, premorbid IQ, current IQ, working and leisure activity) were investigated in terms of their role in protecting language against brain tumours and surgery effects, when considering interactions with demographic, anatomical, and clinical/biological variables. The study found premorbid IQ was the best predictor of pre-operative language integrity. Furthermore, patients with worse pre-operative language integrity and low-to-moderately aggressive tumours showed a mitigating effect of current IQ over surgery consequences. The authors concluded that different cognitive reserve proxies play a role in moderating cognitive decline following brain tumours and surgery. |
| (Capo et al. 2020) | Recurrent gliomas: HGG (n=23; anaplastic n=16, grade IV n=7), LGG (n=17; astrocytoma n=15, oligodendroglioma n=2) | Italy | 2020 | Retrospective | 40 | Before the initial surgery, 4 months following the first surgery, 1 week before recurrence surgery, and 4 months following recurrence surgery. | Surgery (all), radiotherapy (n=19) | Spatio-temporal orientation, executive functions (working memory, verbal fluency), verbal short-term memory (forward), praxis (ideomotor limb apraxia and oral apraxia), language processing tasks (object naming, action naming [Battery for the Analysis of Language Disorders] and language comprehension [Token Test]). | This study aimed to evaluate cognitive function in patients undergoing second surgery for recurrent glioma. It found no significant difference in cognition before and after second surgery, and the number of patients with scores in the normal range did not change from before the first surgery to after the second surgery. The only significant change in the level of performance related to phonological fluency. The authors concluded that repeated glioma surgery is possible without major cognitive damage in the short-term period (four months) after surgery. |
| (Caramanna et al. 2022) | GBM | The Netherlands, Italy, Germany, France | 2022 | Analysis of data collected as part of an RCT | 321 | Within three days of documented use of corticosteroids | Not specified | HVLT-R, COWAT, TMT A and B | This study evaluated the effect of corticosteroid use on neurocognitive function in patients with recurrent glioblastoma, using data from the EORTC trial 26101. It found that patients on corticosteroids had worse neurocognitive function in all tested domains (memory, expressive language, visual-motor scanning speed, executive functioning) compared to those not on corticosteroids. There were significant inverse correlations between corticosteroid use and HVLT-R Free Recall and Delayed Recall scores. |
| (Cayuela et al. 2019) | Oligodendroglioma (WHO grade II and III) | Spain | 2019 | Retrospective | 48 | Minimum of 2 years from completion of treatment (patients split into subgroups) | Surgical resection and radiotherapy alone (21%), surgical resection, radiotherapy, and chemotherapy (79%) | Verbal memory (HVLT-R), visuospatial abilities and visual memory (Rey-Osterrieth Complex Figure Test Copy and Delayed Recall), verbal fluency (COWAT), processing speed (TMT-A and -B). MMSE was used in those unable to complete these tests. | In patients assessed more than 5 years after completion of treatment, severe cognitive impairment was detected in 38%. Cognitive deficits were found in patients assessed 2-5 years after completion of treatment, but no structural brain abnormalities were detected in this group. Cognitive deterioration was strongly associated with loss of grey matter volume and increase white matter damage. After 10 years, over two-thirds of patients had cognitive impairment focused on visual memory and executive functioning, which was associated with grey matter atrophy and leukoencephalopathy; in this group, there was a strong association between grey matter volume loss and increased leukoencephalopathy. |
| (Chang 2018) | LGG (n=7), HGG (n=10), arteriovenous malformation (n=1), chronic inflammation (n=1) | Taiwan | 2018 | Prospective cohort | 19 | Pre-operatively, 1 week, and 3 months after surgery | Surgery (all) | Subtests of the Chinese version of the short-form BDAE were used pre- and postoperatively to assess language function: word discrimination, complex ideational material, repeating phrases/sentences (common and rare phrases/sentences), responsive naming, and visual confrontation naming. Visual object naming (using DO80) and semantic-association tests (using the Pyramid and Palm Tree Test) were used to evaluate intraoperative linguistic performance. | This study evaluated the degree to which recovery of language function following awake brain tumour surgery could be achieved by spontaneous neuroplasticity. To achieve this, changes in postoperative language function were compared to quantified intraoperative linguistic performance. The study found that patients with language impairment during awake surgery had improved language function post-operatively. The intraoperative scores in DO80 and the Pyramid and Palm Tree Test were significantly correlated with postoperative scores in the domains of the short-form BDAE scores, particularly the Pyramid and Palm Tree Test. |
| (Chapman et al. 2016) | Pituitary adenoma (n=9), low-grade glioma (n=8), meningioma (n=6), and other. | USA | 2016 | Prospective cohort | 27 | Before radiotherapy, 6 months and 18 months following radiotherapy | Surgical resection (85%), biopsy (4%), no surgery (11%); temozolomide chemotherapy (15%), no chemotherapy (85%); radiotherapy (all). | Hopkins Verbal Learning Test (revised edition) Total and Percent Retained components of short-term and delayed verbal memory (HVLT-T and HVLT-PR), COWAT, and TMT-B preceded by TMT-A. | In univariate analysis, decreasing axial diffusion and increasing radial diffusion in the parahippocampal cingulum white matter, as measured using DTI, during radiotherapy predicted declines in verbal memory and verbal fluency. In multivariate analysis, baseline neurocognitive score was the only clinical variable that predicted verbal memory change; no clinical variables predicted verbal fluency change. In a multivariate model, increased radial diffusion at the end of radiotherapy significantly predicted decline in verbal fluency 18 months after radiotherapy. |
| (Correa et al. 2007) | LGG | USA | 2007 | Prospective cohort | 40 | Median of 72 months (range 27-142 months) since the diagnosis in the radiotherapy/chemotherapy group, and 22 months (range 3-82 months) in the no radiotherapy/chemotherapy treatment group | Biopsy (n=2), surgical resection (n=36), radiotherapy and chemotherapy (n=1), radiotherapy (n=11), chemotherapy (n=4) | Attention (Digit Span Forward, Digit Span Backward), executive (Brief Test of Attention, TMT-A and -B, Auditory Consonant Trigams - 9 s and 18 s trials, Stroop Color-Word Test-Interference, Verbal Fluency Test), verbal memory (HVLT - Learning / Delayed / Discrimination Index / Percentage Retention), psychomotor (Grooved Pegboard Test-Dominant / Non-Dominant), language (BNT, Animal Fluency), visual-construction (Line Orientation Test). | Patients were grouped into one of two groups for analysis, depending on whether or not they had received radiotherapy and/or chemotherapy. The LGG patients that had received radiotherapy and/or chemotherapy had lower scores than untreated patients across several cognitive domains. In addition, patients who completed treatment longer than 3 years ago and had long disease duration had significantly lower scores on the Non-Verbal Memory domain. The use of multiple antiepileptic medications, treatment history, and disease duration together contributed to low Psychomotor domain scores. Of the patients treated with radiotherapy and/or chemotherapy, 62% had white matter confluence on MRI, compared to only 9% in those that had not received radiotherapy and/or chemotherapy. Comparisons between APOE e-4 carriers (n = 9) and non-carriers (n = 27) on cognitive domain scores revealed no statistically significant differences, but APOE e-4 carriers had lower mean scores on the Verbal Memory domain than the non-APOE-e-4 carriers. |
| (Correa et al. 2008) | LGG | USA | 2008 | Prospective cohort | 25 | Study entry, then 6 months and 12 months later. | None (n = 16), radiotherapy (n=5), radiotherapy and carboplatin chemotherapy (n=1), chemotherapy alone (n=3) | Executive function (Brief Test of Attention, TMT-A and -B, Verbal Fluency), verbal memory (HVLT-Learning, HVLT-Delayed, HVLT-Discrimination Index), non-verbal memory (Brief Visual Spatial Memory Test-Learning, Brief Visual Spatial Memory Test-Delayed, Brief Visual Spatial Memory Test-Discrimination Index), motor (Grooved Pegboard Test Dominant, Grooved Pegboard Test Non-dominant), mood and quality of life (Beck Depression Inventory, Functional Assessment of Cancer Therapy-Brain) | During the initial evaluation, patients that had received some form of oncological treatment (chemotherapy/radiotherapy) had impaired performance in motor speed alone, but scored 1 standard deviation below normative values on tests of executive functions. To contrast, patients that had received no oncological treatment had no cognitive impairment. There was a significant variation over time in nonverbal memory (delayed recall), with patients that had received oncological treatment having an improved performance at the 6 month point to a level similar to the untreated patients, although both groups of patients (treated and untreated) declined in performance slightly at the 12-month point. Of the 25 patients in the study, 15 underwent additional cognitive evaluation 12 to 27 months after the 12-month follow-up assessment, and in this sub-group significant changes were observed between the 12 month point and long-term follow-up in the domains of phonemic verbal fluency, mood, and quality of life; of note, the untreated patients that were seen at short intervals showed a slight improvement whereas the treated patients seen at longer intervals experienced a decline. The study showed disease duration and treatment with radiotherapy/chemotherapy to be associated with reduced performance in non-verbal recall and in some facets of executive function and quality of life. |
| (Correa et al. 2018) | LGG (n=6), HGG (n=14) | USA | 2018 | Prospective cohort | 20 | Two time points: first, a mean of 4 years (SD 3.4 years) after completing treatment, and second a mean 5.21 years later (SD 0.83 years). | Radiotherapy and chemotherapy (85%) or radiotherapy alone (15%). | Digit Span subtest of the WMS-III (LDSF; LDSB), BTA, TMT-A and -B, Phonemic Verbal Fluency (VF), HVLT-R (Learning, Delayed Recall, and Discrimination Index). | There was a significant decline in attention and an almost-significant decline in verbal learning. Comparisons were made according to the APOE status of the patients. This revealed significant differences over time in attention/working memory, with a decline noted in the APOE ε-4 carriers. There were no significant differences seen in imaging-based analyses for β-amyloid deposition (using 18F-florbetaben PET) between APOE ε-4 carriers and non-ε-4 carriers. The results of this study indicate that glioma patients may experience worsening attention and executive functions several years following treatment, and that the APOE ε-4 allele may modulate cognitive decline independently from increased β-amyloid deposition. |
| (Correa et al. 2019) | HGG (n=57), LGG (n=34), primary CNS lymphoma (n=42), other (n=17) | USA | 2019 | Not specified | 150 | Mean of 45 months (SD 50.7) since treatment completion | Radiotherapy and chemotherapy (n=105), chemotherapy alone (n=45) | Attention (Digit Span subtest [Digit Span Forward; Digit Span Backward]; WMS-III; BTA), Graphomotor Speed and Executive Functions (TMT-A, TMT-B, Phonemic Verbal Fluency Test), Verbal Memory (HVLT-R: Learning, Delayed Recall, and Discrimination Index). | This study aimed to address whether genes associated with late-onset Alzheimer's disease, inflammation, cholesterol transport, dopamine and myelin regulation, as well as DNA repair, influenced cognitive outcomes in patients with brain tumours. The study found strong associations between attention, executive functions, memory and 33 single-nucleotide polymorphisms in genes involved in late-onset Alzheimer's disease, inflammation, cholesterol transport, dopamine regulation, myelin repair, DNA repair, cell cycle regulation, and response to oxidative stress. Of note, the genetic findings were not associated with white matter abnormalities on brain MRI scans. |
| (Coşman et al. 2019) | HGG (n=23), LGG (n=6), meningioma (n=8), metastasis (n=6) | Romania | 2019 | Retrospective | 43 | Pre-operatively, seven days, and one month postoperatively | Surgery (all) | BDAE, Denomination d’Object (DO80), HVLT, TMT-B, BLAST, and speech exercise tests | This study evaluated cognitive outcomes in Romanian brain tumour patients undergoing surgery. It found that, at admission, 11.6% of patients were impaired across all measured items and 6.9% in none of the measured items. Impairment was significantly more likely in the HGG patients than the LGG, meningioma, and metastases patients. A general decrease in cognitive function was observed in 20.93% of patients, with 88.88% of these being HGG patients. A favourable outcome was observed in 32.55% of patients, largely LGG and meningioma patients. |
| (Costello et al. 2004) | High-grade brain tumours (n=8), low-grade brain tumours (n=8), and a 'control' group of nonmalignant brain tumours (n=8) | UK and Italy | 2004 | Prospective cohort | 16 | Between 4 and 30 days following surgery, and prior to radiotherapy; a second assessment was carried out between 4.25 and 10.25 months after the first assessment. | Surgery (all) and radiotherapy (15 out of 16) | Intellectual (WAIS-III, NART), memory (Recognition Memory Test, Adult Memory and Information Processing Battery), word processing (Graded Naming Test), perception (VOSP), executive function (HSCT, BSAT, Stroop, Word Fluency, FAS). | There was a differential pattern of cognitive performance between the low- and high-grade brain tumour groups following radiotherapy. The low-grade tumour group's performance was superior across all five main neuropsychological measures, and their pattern of improvement was similar to that of the nonmalignant brain tumour group that had not received radiotherapy. |
| (Douw et al. 2009) | LGG | The Netherlands | 2009 | Prospective cohort | 65 | Mean of 12 years (range 6-28 years) | Biopsy (n=23), surgical resection (n=42), radiotherapy (n=32) | Letter-digit Substitution Test, Concept-shifting Test, Stroop Colour-Word Test, Visual Verbal Learning Test, Memory Comparison Test, Categoric Word Fluency | Patients were compared in their performance of cognitive tasks at two time points - first, a mean of 6 years and second, a mean of 12 years following diagnosis. Patients who had radiotherapy had more deficits that affected attentional functioning at the second assessment point, regardless of fraction dose of radiotherapy administered, when compared to those who did not receive radiotherapy. The patients that received radiotherapy also did worse in measures of executive functioning and information processing speed between the two assessment periods, and their attentional functioning deteriorated significantly between the two assessment points. White-matter hyperintensities and global cortical atrophy were associated with worse cognitive functioning across several domains. In summary, long-term LGG survivors that did not receive radiotherapy had stable radiological and cognitive status, but patients who received radiotherapy demonstrated a progressive decline in attentional functioning, even at fraction doses considered 'safe' (≥2 Gy). |
| (Fang et al. 2014) | LGG (n=32), HGG (n=13), healthy volunteers (n=18) | China | 2014 | Prospective cohort | 63 | Not specified | Surgical resection (all) | Attention Network Test, Digit Span Test, Color Trail Test II, Stroop Test | Patients were analysed according to whether they had a HGG or LGG, and by tumour location. The study found orientation network dysfunction in the parietal lobe tumour group, and execution network deficit in both the frontal and parietal lobe groups. No significant difference was detected in the temporal lobe group compared to healthy controls. The HGG group exhibited more serious functional impairment than the LGG group. No significant correlation was observed between postoperative recovery time and attention impairment. |
| (Forster et al. 2020) | WHO grade IV glioblastoma (n=17), WHO grade III oligodendroglioma (n=2), WHO grade III anaplastic astrocytoma (n=1), WHO grade II diffuse astrocytoma (n=1) | Germany | 2020 | Prospective | 21 | Preoperatively, at the end of the hospital stay, and after a median follow-up period of 5.3 (range: 3.1–12.8) months | Gross total resection (n=15), subtotal resection (n=6), combined radiochemotherapy (n=17), radiotherapy (n=1) | Attention (TMT-A and -B), language (BNT), executive functioning (Regensburg Word Fluency Test, TMT Switching Ratio - TMT-B/A, Verbal Learning and Memory Test - Subtest Intrusion), memory (Verbal Learning and Memory Test - Subtests Total, Recall, Delayed Recall, Delayed Recognition), figurative memory (Pentagon Drawing Test - Delayed Recall), visuospatial functioning (Pentagon Drawing Test - Copy), bimanual coordination (Alternating Opening and Closing of Fists, Alternating Pronation and Supination, Luria Sequence, Finger Tapping Sequence), object recognition (Tactile Object Recognition, Tactile Letter Recognition), orientation (MMSE), depression and anxiety (Hospital Anxiety and Depression Scale). | This study evaluated the neuropsychological outcomes following glioma resection in the corpus callosum. The study found gross total resection was possible in 15 of the 21 patients included. All cognitive domains were affected in up to two-thirds of patients preoperatively, and within the first few postoperative days, the proportion of impaired patients increased across all studied neurocognitive domains. However, after 6 months, there were significantly fewer patients with impairments in attention, executive functioning, memory and depression. The authors concluded that the study results support the potential benefits of resections of tumours located in the corpus callosum. |
| (Friedman et al. 2003) | Third ventricle region tumours: extra-axial intraventricular (n=18) and infiltrative tumour (n=15) | USA | 2003 | Retrospective | 33 | Mean 31 months after diagnosis (median, 8 months; range, 1 week to 250 months). Pre-operative evaluation also available in 2 patients. | Surgery for the tumour (n=26), including treatment for hydrocephalus (n=20) | Intellectual tests (Verbal IQ, Information, Digit Span Total, Arithmetic, Comprehension, Similarities, Block Design, Digit Symbol), verbal memory (Word Recall-Immediate [Verbal Selective Reminding Test, Hopkins/Sum of Trials], Word Recall-Delayed [Verbal Selective Reminding Test], Word Recognition [Hopkins/Discrimination Index], Story Recall-Immediate and -Delayed [WMS-Revised/Logical Memory]), visual memory (BVRT error score), attention/executive functioning (Digit Span Forward and Digit Span Backward [WAIS-R], Booklet Category Test, TMT-A and -B), manual speed and dexterity (Grooved Pegboard-Right and -Left), visual perceptual ability (Facial Recognition), language ability (Multilingual Aphasia Examination; Visual Naming, Fluency [COWAT], Language Comprehension (Token Test). | Relative to normative values, a significantly elevated frequency of cognitive impairments spanning the domains of memory, executive functioning, as well as fine manual speed and dexterity was observed. No difference in neuropsychological scores were observed between those that did and did not undergo surgery. No differences were observed based on the surgical approach used, tumour infiltration, or history of cranial radiotherapy treatment. For the two patients with pre- and post-operative cognitive testing data available, memory impairments were identified prior to and following surgery, and one patient exhibited a major improvement after surgery in a measure of mental flexibility and problem solving. |
| (Gehring et al. 2011) | LGG (n=54), anaplastic glioma (n=10) | The Netherlands | 2011 | Analysis of data collected as part of an RCT | 64 | Baseline, immediately following cognitive rehabilitation (or an equivalent time point for the control group) and at 6-months follow-up | Radiotherapy (n=39 in intervention group), chemotherapy (n=7); surgery rates not specified but cohort based on the subjects studied in (Gehring et al. 2009), of whom approximately two-thirds underwent surgical resection, one-third underwent biopsy, and a few underwent no surgery at all. | Screening tests (Dutch Adult Reading Test, Drie-Minuten-Toets [Three-Minute-Test], Stroop Color-Word Test, Letter Digit Substitution Test, Memory Scanning Test, Visual Verbal Learning Test-direct and delayed recall, Concept Shifting Test, Category Fluency-animals [from the Groningen Intelligence Test]), neuropsychological tests for the evaluation of intervention effects: 1. attention (Stroop Color-Word Test, Digit Span from WAIS-R, Letter Digit Substitution Test, Memory Scanning Test, Test of Everyday Attention), 2. verbal memory (Visual Verbal Learning Test-direct and delayed recall), 3. executive functions (Concept Shifting Test, Letter Fluency, Category Fluency-animals, Behavioural Assessment of the Dysexecutive Syndrome, Test of Everyday Attention); cognitive symptoms (Cognitive Functioning Scale-from the Medical Outcomes Study, Burden [study-specific measure], Cognitive Failure Questionnaire, SF-36 from the Medical Outcomes Study, Multidimensional Fatigue Inventory, Community Integration Questionnaire), additional subjective measures (motivation, evaluation) | This study was a follow-on from an RCT (Gehring et al. 2009) but focused on specific patient factors that predicted responsiveness to the cognitive rehabilitation programme, as opposed to the group-level effects analysed in the 2009 study. The study created four categories of potential predictors of improvement: sociodemographic variables, clinical variables, self-reported cognitive symptoms, and objective neuropsychological test performance. The study found that almost 60% of participants had improved reliably following the cognitive rehabilitation programme, and significant predictors of improvement included age and education. Younger patients were more likely to benefit from the intervention. In the control group, higher education was associated with improvement. Age should thus be accounted for when using cognitive rehabilitation programmes, and modify such programmes to improve the likelihood of their success in older participants. |
| (Giovagnoli et al. 2007) | Several types of low-grade brain tumours, including ganglioglioma (n=8), oligodendroglioma (n=5), fibrillary astrocytoma (n=3), DNET (n=2), pilocytic astrocytoma (n=2), xanthoastrocytoma (n=1), hamartoma (n=1), ependymoma (n=1) and subependymoma (n=1), as well as healthy controls (n=36). | Italy | 2007 | Prospective cohort | 60 | Average of 11.25 (±13.16) months after surgery patients with left temporal lobe epilepsy and 8.50 (±7.44) months after surgery in the patients with right temporal lobe epilepsy. | Mesial lesionectomy (n=12), lesionectomy (n=6), lateral plus mesial temporal cortectomy including the lesion (n=6). | Verbal tests (Word Learning, Short Story, Distractor Test), visual tests (Design Learning, Rey Figure, Distractor Test), general intelligence (RCPM), attention (Attentive Matrices) | This study compared neuropsychological status in patients with temporal lobe epilepsy associated with low-grade brain tumours. Patients with left temporal lobe epilepsy had significant verbal impairment compared to patients with right temporal lobe epilepsy. Visual deficits were present in both groups relative to controls. Following surgery, there was no significant decrease in mean verbal or visual memory scores related to the operated side. Some memory abilities associated with the contralateral temporal lobe improved. Memory scores postoperatively were related to preoperative scores, the laterality of the surgery, age, and education. Preoperative memory performance was the strongest predictor of postoperative memory performance. |
| (Goldstein et al. 2003) | Low-grade supratentorial tumours (including astrocytoma, oligodendroglioma, ependymoma, meningioma, pineal, pituitary tumours, and others) | USA | 2003 | Prospective cohort | 55 | Six weeks following surgery | Surgery - biopsy / resection (n=49), none (n=6) | Auditory Selective Attention Test, Bells Test, Symbol Digit Modality Test - Oral Version, Visual Pursuits Test of the Employee Aptitude Survey, WCST, Digit Span Test, Visual Memory Span Test, PASAT | Patients were grouped into a "superficial" regional group and classified in multiple ways according to brain regions and laterality. These subjects were compared to deep tumour patients and a demographically normal control group. The results indicated that patients with brain tumours performed worse than normal controls in some tests, but better in others. Differences between tumour groups were only found in the Digits Forward test, where the deep tumour group performed significantly worse than the superficial regional group and the combined anterior and combined posterior tumour groups. The deep tumour group performed similarly to the other patient groups in the remaining attention tests, indicating that these tumours are associated with particularly poor attentional performance. |
| (Haldbo-Classen et al. 2020) | Meningioma (n=21), pituitary adenoma (n=17), WHO grade II glioma (n=18), WHO grade III glioma (n=12), medulloblastoma (n=7), other rare brain tumours (n=3) | Denmark | 2020 | Cross-sectional | 78 | Mean of 4.6 years (range 1–9 years) since radiotherapy | Gross total resection (n=34), partial tumour resection (n=30), biopsy (n=11), chemotherapy (n=19), radiotherapy (all) | Processing speed (TMT-A, WAIS-IV Coding), attention and working memory (PASAT, WAIS-IV Digit Span), verbal learning and memory (HVLT-R Total and HVLT-R Delayed), verbal fluency (COWAT Animals and Letter S), executive function (TMT-B, Stroop Interference test) | This study evaluated the relationship between radiotherapy dose to the hippocampus and learning and memory functions a mean of 4.6 years (range 1–9 years) since radiotherapy. The study found high radiotherapy dose to the left hippocampus was associated with impaired verbal learning and memory. To contrast, radiotherapy dose to the left hippocampus, left temporal lobe, left frontal lobe, and total frontal lobe were associated with verbal fluency impairment. Radiotherapy dose to the brain and thalamus were associated with impaired processing speed. |
| (Hendriks et al. 2018) | LGG (WHO grade I, n=6; WHO grade II, n=43), HGG (WHO grade III, n=10) | The Netherlands | 2018 | Prospective cohort | 59 | Baseline one week prior to surgery and postoperatively after one year | Surgical resection (all) | Attention (Digit Span, Stroop Color Word Test), information speed (Stroop Color Word Test, TMT, Letter Digit Modalities Test), visual construction (ROCFT), verbal memory (Auditory Verbal Learning Test), visual memory (Location Learning test, ROCFT), working memory (Digit Span, Memory Comparison Test), execution (TMT, Categoric Word Fluency Test, Letter Word Fluency Test, BADS) | MRI scans were used to create resection cavity maps to compare patients with and without new cognitive deficits one year following surgery relative to baseline (preoperatively). Cognitive improvement in any domain was found in 10 (17%) patients, cognitive decline in any domain was found in 25 (42%), and decline in more than one domain in 10 (17%). Attention (n=10; 17%) and information processing speed (n=9; 15%) were the commonest deficits identified. The surgical resection regions that were associated with decline in more than one cognitive domain were located most commonly in the right hemisphere. No specific region was association with decline in attention. Decline in information speed was associated with several regions, including the frontal pole and the corpus callosum. |
| (Hoffermann et al. 2017) | Meningioma (n=76), HGG (n=55), LGG (n=23), metastasis (n=26), vestibular schwannoma (n=12), others (lymphoma, haemangioblastoma, epidermoid; n=3) | Austria | 2017 | Prospective cohort | 196 | One-to-two days pre-operatively and 3-4 months postoperatively | Surgical resection (all); number of patients that received chemotherapy and/or radiotherapy not specified | Verbal short-term memory (Digit Span), working memory (Two Back), alertness (Simple Reaction), selective attention (Go/No Go), selective attention and cognitive flexibility (Inverted Go/No Go), verbal learning and recognition (Verbal Memory), figural learning and recognition (Figural Memory), phonematic literal word fluency (Phonematic Fluency) | Participants completed a computer-based neurocognitive assessment tool before and after surgery. The study found that the majority of patients with malignant gliomas, metastases, and meningiomas had significant deficits in multiple neurocognitive domains; however, most of them either improved or did not decline following surgery. Brain tumour location did not appear to be correlated significantly with neurocognitive deficits. |
| (Hornak et al. 2003) | A combination of tumour and non-tumour pathologies. One patient each with oligodendroglioma and astrocytoma. | UK | 2003 | Prospective cohort | 35 | Range of 1 to 22 years between surgery and taking part in the study. | Surgery (all) | Vocal Emotion, Facial Expression, Subjective Emotional Change Questionnaire, Social Behaviour Questionnaire | Unilateral surgical removal of the prefrontal cortex, including the fronto-orbital or anterior cingulate cortex, was found to result in emotional dysregulation with impaired voice and face expression identification. |
| (Incekara et al. 2018) | LGG (n=47), HGG (n=30) | The Netherlands | 2018 | Prospective cohort | 77 | Between the initial diagnosis (mean 9 days; SD 20 days) and surgery (mean 44 days; SD 30 days) | Surgery (all) | Language (AAT, Token Test, BNT, Category Fluency, Letter Fluency), memory (15 Words Test, imprinting and recall), attention/executive function (TMT-A, -B, and BA, Stroop I, II, III, and interference) | This study aimed to evaluate whether cognitive functioning in patients with gliomas were associated with white matter tract changes. It found significant correlations between the fractional anisotropy of the arcuate fasciculus and results of the repetition test for the language domain, and between fractional anisotropy of the inferior frontooccipital fasciculus and results of the imprinting test for the memory domain and the attention test for the attention and executive function domain. It is also worth noting that the subjects in this study were all assumed to have a LGG preoperatively based on clinical and imaging characteristics, but the postoperative histopathological analysis confirmed non-LGG tumours in over one-third of cases. |
| (Irle et al. 1994) | Tumour types not specified, but study comprised of brain tumour patients (n=141), clinical control patients (n=29), and normal controls (n=18) | Germany | 1994 | Prospective cohort | 188 | 1-5 days pre-operatively and 2-10 days postoperatively. | Surgery (all) | Short form of WAIS, TMT-A and -B, Token Test, Stroop Test, Benton Test, paired associate learning using semantically interfering lists of words, visual memory design using incomplete figures and words, Verbal and Visual Span of WMS-R, and a mood questionnaire. | Study subjects were divided into groups according to the location of the tumour. Negative mood changes were identified after the resection of brain tumours involving heteromodal cortices located either prefrontal or temporoparietal, whereas positive mood changes were identified after lateral frontal resections. Postoperatively reported levels of fatigue, irritability/anger, and anxiety/depression were positively correlated with the extent of cognitive impairment, significantly so for paired associate learning, Similarities, Block Design, Picture Completion, and Visual Span. In the frontal and parietal lesion group, there were significantly positive relationships between the post-operative levels of fatigue, irritability/anger, and anxiety/depression and the number of deficits in the Stroop Test, visual memory tests, Block Design, Similarities, and Picture Completion tests. However, when analysing by lesion group in multivariate analysis, basic cognitive and attentional performance did not seem to contribute significantly to the reported mood levels. |
| (Jaeckle et al. 2021) | WHO grade III anaplastic oligodendroglioma | USA, The Netherlands, Germany, Canada, Switzerland | 2021 | RCT | 36 | Baseline and within the first 3 months of treatment | Gross total resection (n=17), subtotal resection (n=16), biopsy (n=3), with participants randomised to either radiotherapy alone (n=12), radiotherapy with concomitant temozolomide (n=12), or temozolomide alone (n=12) | HVLT-R Immediate Recall, COWAT, TMT-A and -B, HVLT-R Delayed Recall and Delayed Recognition | This study was a RCT of adult patients with newly diagnosed WHO grade III oligodendroglioma comparing radiotherapy alone to chemoradiotherapy and chemotherapy alone. The primary outcome was overall survival, but the study found no difference in neurocognitive decline from baseline to 3 months between the three arms. |
| (Jagaroo et al. 2000) | Multiple types, including intrinsic and extra-axial tumours (n=27), and controls (n=10) | USA | 2000 | Prospective cohort | 37 | Between 0.5 and 90 months after surgery (in those that underwent surgery) | Surgery (n=25), radiotherapy (n=16) | Benton Line Orientation Test, Cross Test, Shape Rotation Test, Royer-Holland Test of Transformational Rotations, Hooper Visual Organization Test, Form Assembly Test | Patients were grouped into one of four tumour groups - depending on either left or right posterior parietal cortex or prefrontal cortex location of the tumour. None of the four groups were significantly different with regards to visuospatial performance when compared to the control group of normal adults. |
| (Jehna et al. 2017) | LGG (n=11), HGG (n=16) | Austria and Germany | 2017 | Prospective cohort | 27 | Prior to surgery. | Not specified (some patients had developed recurrence after prior surgery) | Spontaneous speech (semi-structured interview, semantic and phonemic Verbal Fluency Tests), the Token Test, and a short form of the BNT. | The study aimed to investigate the relationship between language performance and the arcuate fasciculus on diffusion tensor imaging. The results indicated that patients without language deficits tended to have an arcuate fasciculus that was symmetric or lateralized to the right, but patients with deficits in language significantly more often demonstrated a left-lateralized posterior segment of the arcuate fasciculus. Patients with HGG had more severe language deficits than those with LGG. The only significant predictors of language deficits in this group included the laterality index of the posterior arcuate fasciculus segment and tumour grade. |
| (Kaleita et al. 2004) | Multiple types, including glioblastoma multiforme (37.2%), anaplastic astrocytoma (16.7%), low-grade astrocytoma (12.7%), and oligodendroglioma (10.1%) | USA | 2004 | Prospective cohort | 79 | Not specified | Neurosurgical debulking (65.8%), radiotherapy (75.3%), and previous or current chemotherapy (51%). | TMT-A and -B, Digit Span, and Symbol Digit Modalities | The study found that frontal tumour location and increasing age were predictors of cognitive outcomes in brain tumour patients; the latter even persisted in absolute terms when comparisons were based on age-standardized versions of neurocognitive outcomes. Major depressive disorder was marginally associated with outcomes. Interestingly, however, surgical interventions and radiotherapy were not strongly associated with test performances. |
| (Kelm et al. 2017) | LGG (n=20), HGG (n=41) | Germany | 2017 | Prospective cohort | 61 | Not specified | Surgery (all) | AAT, object naming, verb generation, counting | This study investigated whether clinical outcomes in patients undergoing awake craniotomy for gliomas were different in those having surgery with and without a neuropsychologist present during the operation. The study found no difference in permanent surgery-related language deterioration between patients that underwent surgery with a neuropsychologist and those that underwent surgery without. |
| (Kessels et al. 2000) | Neuroepithelioma (n=2), gangliocytoma (n=1), meningioma (n=4), benign cyst (n=1), astrocytoma (n=1), ganglioglioma (n=1), and healthy controls (n=24) | The Netherlands | 2000 | Prospective cohort | 34 | More than 1 year after surgery | Surgery (all) | Object-location memory (object-location binding, positional memory, combined process), visuospatial construction, object memory | Spatial memory problems were identified mainly in patients with lesions in either the right hemisphere or in the parietal lobe. |
| (Klein et al. 2001) | Anaplastic astrocytoma (n=36), oligodendroglioma (n=7), glioblastoma (n=25), non-small cell lung cancer (n=50), age- and gender-matched controls (n=118) | The Netherlands | 2001 | Not specified | 236 | Following surgery but before radiotherapy (glioma patients) or chemoradiotherapy (non-small cell lung cancer patients) | Biopsy (n=11), resection (n=57), no surgery (all non-small cell lung cancer patients) | Overall cognitive performance (MMSE), intelligence (Dutch Adult Reading Test), perception (Line Bisection Test, Facial Recognition Test, Judgment of Line Orientation Test, Letter-Digit Substitution Test), memory (Visual Verbal Learning Test, Working Memory Task), attention and executive function (Stroop Color-Word Test, Categoric Word Fluency, Concept Shifting Test) | The aim of this study was to evaluate health-related quality of life and cognitive function in patients with HGG following surgery compared to a group of patients with non-small cell lung cancer and age- and sex-matched healthy controls. The study found worse neurocognitive function in the glioma patients compared to the non-small cell lung cancer group. Poor performance in timed tasks was largely attributable to visual and motor deficits in the glioma group. Tumour laterality affected neurocognitive function in a predictable manner (e.g. patients with right-sided tumours had significantly lower scores on tasks involving right-hemisphere function including line bisection compared to those with left-sided tumours), whereas EOR had no significant effect. Corticosteroid use was associated with better recognition memory, whereas anti-epileptic drug use correlated negatively with working memory capacity. |
| (Klein et al. 2002) | LGG patients (n=195), low-grade haematological patients (n=100), and healthy controls (n=195) | The Netherlands | 2002 | Prospective cohort | 490 | Mean of 6.1 years (SD 4.1) in the radiotherapy group and 5.1 years (SD 2.9) in the non-radiotherapy group | Biopsy (n=55), surgical resection (n=49), radiotherapy (n=104) | Intelligence (Dutch Adult Reading Test), perception and psychomotor speed (Line Bisection Test, Facial Recognition Test, Judgment of Line Orientation Test, Letter-Digit Substitution Test), memory (Visual Verbal Learning Test, Working Memory Task), attention and executive function (Stroop Colour-Word Test, Categoric Word Fluency Task, Concept Shifting Test) | Patients with LGG had worse performance in all cognitive domains relative to those with low-grade haematological disorders and even more so when compared to the healthy controls. The administration of radiotherapy was associated with worse cognitive outcomes, but in the memory domain was only affected in those receiving fraction doses more than 2 Gy. The use of antiepileptic medication was significantly associated with disability in attentional and executive function. The authors concluded that the tumour itself has the most effect on cognitive function and that radiotherapy mainly affects cognitive function when high fraction doses are administered. |
| (Klein et al. 2003) | LGG | The Netherlands | 2003 | Prospective cohort | 156 | Not specified (but cohort are derived from a previous study (Klein et al. 2002), where testing was performed at a mean of 6.1 years (SD 4.1) in the radiotherapy group and 5.1 years (SD 2.9) in the non-radiotherapy group | Biopsy (33%-57%), surgical resection (43%-67%), radiotherapy (42-61%) [ranges provided as patients were split into six groups according to their epilepsy burden and treatment status] | Line Bisection Test, Facial Recognition Test, Judgment of Line Orientation Test, Letter-Digit Substitution Test, Visual Verbal Learning Test, Working Memory Task, Stroop Color-Word Test, Categoric Word Fluency Task, Concept Shifting Test | Information processing speed, psychomotor function, attentional functioning, verbal and working memory, and executive functioning were significantly worse in the LGG group than in healthy controls. Higher epilepsy burden was associated with significant reductions in attentional and memory functioning, which could primarily be attributed to the use of anti-epileptic medication: those on one or more anti-epileptic medications had significantly impaired information processing capacity, psychomotor function, attentional functioning, working memory capacity and executive functioning than those no taking anti-epileptic medications. Only verbal memory performance was similar between those taking and not taking anti-epileptic medications. |
| (Klein et al. 2021) | WHO grade II astrocytoma (n=37), WHO grade II oligoastrocytoma (n=24), WHO grade II oligodendroastrocytoma (n=37) | The Netherlands, France, Israel, Spain, Belgium, Switzerland, USA | 2021 | Analysis of data collected as part of an RCT | 99 | Baseline (within 6 weeks before randomisation and before the start of treatment), six months and 12 months | Total removal (n=17), partial removal (n=34), biopsy (n=47), with patients randomised to receive either radiotherapy (n=52) or temozolomide (n=46) | Memory (Visual Verbal Learning Test-Immediate Recall, Total Recall, Learning Capacity, and Delayed Recall) | This study was a follow-on from an RCT comparing radiotherapy to temozolomide chemotherapy alone as a primary oncological treatment for high-risk LGG. This study focused on memory function in the study participants. The study found no baseline difference in memory between the two arms. Over time, patients in both arms showed improvement in Immediate Recall and total Recall, although the improvement in radiotherapy patients was delayed. Memory functioning was not found to be associated with radiotherapy gross, clinical, or planned target volumes. The authors concluded that in the first year after treatment, there is no evidence of a significant deleterious effect on memory function of radiotherapy compared to temozolomide chemotherapy. |
| (Kleinberg et al. 1993) | Various types of high-grade and low-grade gliomas | USA | 1993 | Prospective | 30 | Median of 42 months (range: 16-183 months) | Biopsy (n=5), partial resection (n=5), complete resection (n=20), radiotherapy (all) | A self-designed memory scale (normal/mild/moderate/severe) | This study evaluated memory function, performance status, and employment history in patients with gliomas that were alive and disease-free more than one year following cranial radiotherapy. The study found no progressive deterioration in neuropsychological function after completion of cranial radiotherapy. |
| (Laack et al. 2005) | LGG | USA | 2005 | Prospective cohort | 20 | Baseline (before radiotherapy) and approximately 18-month intervals for as long as 5 years after completing radiotherapy | Gross total resection (n=2), biopsy (n=18), low-dose (50.4 Gy) radiotherapy (n=10), conventional (64.8 Gy) radiotherapy (n=10) | MMSE, WAIS-R, Auditory-Verbal Learning Test, BVRT, TMT-A and -B, Stroop | This study evaluated the effects of cranial radiotherapy on cognitive function in patients with LGG. The study found baseline test scores were below average compared to age-specific norms. Compared to the baseline test scores, scores at the second evaluation were higher across all psychometric measures but the difference was not statistically significant. No changes in cognitive performance were seen when analysing results by age, treatment, tumour location, tumour type, or extent of resection. |
| (Lang et al. 2017) | LGG (n=5), HGG (n=11) | Canada | 2017 | Prospective cohort | 13 | Pre-operatively and also on average 32.4 ± 1.3 days after surgery | Surgery (all), adjuvant therapy (n=2) | The National Institutes of Health Toolbox Cognitive Battery (NIHTB-CB). NIHTB-CB consists of 7 tasks: Dimensional Change Card Sort, Flanker Inhibitory Control and Attention, Picture Sequence Memory, Picture Vocabulary, Oral Reading Recognition, Pattern Comparison Processing Speed, and List Sorting Working Memory | In this study, pre-operative cognitive status was correlated with fMRI-determined patient-specific connectivity in the frontoparietal network, and whether connectivity could predict neuropsychologic outcome following surgery was also evaluated. The study found higher average connectivity within the frontoparietal network to be associated with lower composite cognitive scores and higher connectivity of the parietal region of the tumour-affected hemisphere to be associated specifically with lower fluid cognition. Furthermore, lower connectivity of the parietal region of the non-tumour hemisphere was associated with worse neuropsychologic outcome 1 month after surgery. |
| (Latini et al. 2021) | WHO grade II astrocytoma (n=12), WHO grade III astrocytoma (n=11), WHO grade II oligodendroglioma (n=4), WHO grade III oligodendroglioma (n=9) | Sweden | 2021 | Not specified | 46 | Before surgery | None | Digit Span, Coding, TMT trials 1–5, RAVLT Swedish Version, Brief Visuospatial Memory Test-Revised, ROCFT, Zoo Map Test, Color-Word Interference Test trials 1–4, Hospital Anxiety and Depression Scale, BeSS, BNT, Token Test (Version C), Neurolingvistisk afasiundersökning (A-ning), Word fluency (FAS, Animals and Verbs), LS, Reading Words and Nonwords, Oralmotorisk diadochokinesi, STAVUX | This study investigated the relationship between preoperative assessment and intraoperative findings in eloquent tumours operated on through awake surgery. The possible mechanisms of tumour-induced brain reorganisation were also studied. The study found impaired neuropsychological function to be associated with invasive tumours, crucial location (left opercular-insular/sub-insular region), and a higher risk of eloquent tumours. Preoperative neuropsychological impairment was the only identified variable that predicted the intraoperative finding of eloquent tumours. |
| (Lee et al. 2020) | WHO grade II tumour (n=4), anaplastic tumour (n=5), glioblastoma (n=17) | USA | 2020 | Retrospective review of a prospective registry | 26 | One day postoperatively, postoperatively day 2–3, and 1-3 months following surgery | Surgery (all) | Quick Aphasia Battery, Text Reading, 4-syllable Repetition, Picture Naming, Syntax, Auditory Stimulus Naming | This study evaluated the effect of resection of brain regions identified as having high functional connectivity through magnetoencephalography on language performance. The study found that 70% of patients that underwent resection of high functional connectivity tissue developed early postoperative language deficits compared to 33% of patients where no high functional connectivity tissue was resected. Among those who underwent high functional connectivity tissue resection, those with a higher proportion of such tissue being resected were more likely to have a language deficit. By 2.2 months following surgery (range: 1–6 months) the correlation between high functional connectivity tissue resection and auditory stimulus naming as well as syntax had resolved in patients without stroke or early tumour progression. |
| (Leonetti et al. 2021) | LGG (n=80), HGG (n=65) | Italy | 2021 | Prospective cohort | 145 | Baseline (pre-operative) and 1, 3, 6, and 12 months post-operatively | Surgery (all), chemotherapy (n=46 in LGG group, n=64 in HGG group), radiotherapy (n=42 in LGG group, n=64 in HGG group) | Language (Token Test, Picture Naming Test, Verbal Fluency-Phonemic and Semantic), attention and executive functions (Attentive Matrices, TMT, Stroop Test), memory (Digit Span Backward, RAVLT, Recall Rey Figure), praxis (Ideomotor Apraxia Test, Oro-facial Apraxia Test) | This study aimed to evaluate the prevalence of mood disorders and decline in quality of life in glioma patients, and factors associated with these two variables. In the patients with LGG, from pre-operatively to one year following surgery, mood disorders and low quality of life were associated with the presence of cognitive deficits. |
| (Leroy et al. 2021) | WHO grade II (n=12), WHO grade III (n=4), WHO grade IV (n=4) | France, Switzerland | 2021 | Prospective | 20 | Preoperatively and 3 months after surgery | Surgery (all), combined chemoradiotherapy (n=6), chemotherapy or radiotherapy alone after surgery (n=4) | Overall cognitive efficiency (MoCA), working memory, verbal and visual memory, learning, executive tasks, attention, language (speaking and listening), visuoconstructional and gnosic functions, reasoning, facial emotion recognition (Batterie de Cognition Sociale), ability to infer a character's intention (V-Comics) | This study evaluated the outcomes following intraoperative MRI-guided surgery under general anaesthesia for frontotemporoinsular gliomas without cortical or subcortical mapping. The average extent of resection was 92%. No significant neuropsychological impairment was noted by the study authors, except for empathy distinction in less than 40% of patients. |
| (Liu et al. 2015) | WHO grade II glioma (n=21), WHO grade III glioma (n=84), WHO grade IV glioma (n=125), unclassified (n=3) | USA, Taiwan | 2015 | Prospective | 233 | Before surgery | None | Memory (HVLT-R), processing speed (TMT-A), executive function (TMT-B, COWAT) | This study evaluated the effect of genetic variants on neurocognitive function in 233 newly diagnosed glioma patients before surgical resection. The genetic analysis included the genotype frequencies of 10,967 single nucleotide polymorphisms in 580 genes related to five pathways (inflammation, DNA repair, metabolism, cognitive, and telomerase). The study found 18 polymorphisms associated with processing speed and 12 with executive function. The strongest signals for processing speed were in IRS1 rs6725330 in inflammation pathway, ERCC4 rs1573638 in DNA repair pathway, and ABCC1 rs8187858 in metabolism pathway. The strongest associations with executive function were in NOS1 rs11611788 and IL16 rs1912124 in inflammation pathway, and POLE rs5744761 in DNA repair pathway. Significant gene polymorphism-dosage effects were observed for processing speed and executive function. |
| (Liu et al. 2021) | HGG (n=9), LGG (n=8) | China | 2021 | Not specified | 17 | Not specified | Surgery (all) | WAIS-Chinese revision, digital symbol substitution test, mapping test, digit span test, similarity test, math test, visuospatial test and visual memory test (WMS-Chinese revision) | This study evaluated the relationship between neural activity determined on resting-state fMRI and neurocognitive function in patients with unilateral temporal gliomas. It found that intrinsic regional activity in the contralesional hippocampus and parahippocampal regions was associated negatively with visuospatial scores, but no correlation was observed between intrinsic regional activity and Digital Span Test, Digital Symbol Substitution Test, Memory, Mapping, Similarity, or Math Test scores. |
| (Loit et al. 2019) | GBM (n=61), lower-grade glioma (including WHO grade II and III, n=54) | France, Canada | 2019 | Retrospective | 115 | Prior to and 4 months after surgery | Total or sub-total resection (n=44 in GBM group, n=48 in lower-grade glioma group), partial resection (n=17 in GBM group, n=6 in lower-grade glioma group) | Semantic fluency | This study attempted to correlate stroke occurrence with cognitive outcomes in patients following glioma surgery, with a focus on semantic fluency scores. The study found that the presence of small infarcts correlated with a slight decrease in semantic fluency scores at 4 months. |
| (MacPherson et al. 2017) | High-grade tumours (n=18), low-grade tumours (n=22), meningioma (n=24), and healthy controls (n=142) | UK and Italy | 2017 | Prospective cohort | 206 | Average of 13.94 (SD 28.29) months after 'damage' | Surgery (all) | Executive function (Phonemic Fluency 'S', Stroop), fluid intelligence (Raven's Advanced Progressive Matrices), speed (TMT-A), verbal short-term memory (Digit Span subtest from the WAIS-III), naming (Graded Naming Test), perception (Object Decision Test from the VOSP) | This study aimed to evaluate the independent effects of two cognitive reserve proxies, education and NART IQ, on a range of cognitive domains in patients with focal frontal lesions. Only NART IQ predicted executive and naming performance. Neither education nor NART IQ predicted performance on fluid intelligence, processing speed, verbal short-term memory, or perceptual abilities. Education and NART IQ did not modify the effect of lesion severity on cognitive impairment. Age significantly predicted performance on executive tests and most of the other cognitive measures except verbal short-term memory and naming. Age was the only predictor for fluid intelligence, suggesting that age plays a role in executive performance over and above the contribution of cognitive reserve proxies with focal frontal lesions. The authors concluded that the studied cognitive reserve proxies do not appear to modify the relationship between cognitive impairment and frontal lesions. |
| (MacPherson et al. 2020) | High-grade tumour (n=27), low-grade tumour (n=37), meningioma (n=49), stroke (n=53) | UK | 2020 | Prospective cohort | 166 | Average of 23.74 (SD 48.11) months and 19.78 (SD 56.98) months in the frontal and non-frontal groups, respectively | Surgery (all tumour patients) | Executive abilities (phonemic fluency S), intelligence (WAIS-III – full Scale IQ), speed of information processing (TMT-A), and naming (Graded Naming Test) | This study evaluated whether cognitive reserve predicts cognitive performance of patients with non-frontal lesions compared to frontal lesions. NART IQ was found to predict executive, intelligence, and naming performance. Age was also found to significantly predict performance on executive and processing speed tests. Being part of the frontal group predicted executive and naming performance, while being part of the non-frontal group predicted intelligence. The authors concluded that age, lesion group, and literacy attainment have independent roles in predicting cognitive performance following stroke or brain tumour, but the relationship between cognitive reserve and focal brain damage does not differ in relation to frontal and non-frontally located lesions. |
| (Mandonnet 2019) | IDH-mutant insular gliomas | France | 2019 | Retrospective | 12 | Preoperatively and 4 months postoperatively | Surgery (all) | Language (DO80 and literal as well as categorical word fluencies), nonverbal semantic association (Pyramid and Palm Tree Test), mathematics (3 basic arithmetic mental computations), praxis (copy of the Rey figure), memory (forward and backward digital span, verbal span, free and cued selective (16 items or 48 items) reminding test, delayed copy of the Rey figure), attention (d2 attention test), spatial awareness (Bell test, line bisection), executive functions (Stroop test, TMT-A and -B), emotion recognition. | This study aimed to evaluate the neuropsychological outcomes following surgery for insular gliomas. The study found a slight deterioration in lexical abilities and verbal memory in patients with left-sided tumours, and performance in cognitive flexibility often declines irrespective of tumour laterality. |
| (Mandonnet et al. 2015) | LGG (n=10), HGG (n=15) | France, the Netherlands, UK | 2015 | Retrospective | 25 | Pre-operatively, 3-5 days postoperatively, and 3 months postoperatively | Surgery (all), chemotherapy (n=7), radiotherapy (n=1) | Language (DO80, Literal and Categorical Word Fluency, Pyramidal and Palm Tree Test), praxis (copy of the Rey figure), calculus (3 functions-1 addition, 1 subtraction, 1 multiplication), memory (Forward and Backward Digital Span, Verbal Span, Free and Cued Selective Reminding Test, long-term reproduction of the Rey Figure), attention (D2 of the Attention Test), executive functions (Stroop test), TMT-A and -B, PASAT. | This study evaluated the outcomes in a cohort of patients with glioma undergoing awake craniotomy. Cognitive testing before and after surgery was performed in 10 patients with LGG. The study found no change in cognitive function in 4 of these patients, improvement in some test scores in 2 patients, and deterioration in some test scores in 3 patients. |
| (Maschio et al. 2020) | HGG (n=8), glioblastoma (n=7), LGG (n=8), meningioma (n=2), metastasis (n=1) | Italy | 2020 | Prospective cohort | 26 | Baseline and 6 month follow-up | Gross total resection (n=11), partial resection (n=12), biopsy (n=3), chemotherapy (n=22), radiotherapy (n=11) | Global cognitive status (MMSE), nonverbal abstract reasoning (RCPM), selective visual attention and oculomanual coordination (TMT and Visual Search), immediate and delayed recall-verbal learning (RAVLT), immediate and delayed recall-visuospatial learning (ROCFT), visuospatial and visuoconstructive abilities (ROCFT-copy), verbal processing speed (phonemic and semantic fluency), executive functions (Tower of London). | This study evaluated the effectiveness of perampanel as an add-on therapy in patients with brain tumour-related epilepsy, including effects on quality of life, cognition, and mood. The study population comprised of patients with a number of brain tumour types and uncontrolled seizures. The study found no change in neurocognitive outcomes associated with the use of perampanel at six months compared to baseline. |
| (Mattavelli et al. 2019) | HGG (n=17), LGG (n=17) | Italy | 2019 | Not specified | 34 | Before and after surgery (±7 days) for all tests, with a subgroup of patients tested again at 3 months for the visual (n=21) and auditory (n=20) emotion recognition tasks. | Surgery (all) | Attentional matrices, Token Test, RCPM, Picture-naming Task, Ekma 60 Faces Test | This study focused on visual and auditory emotion recognition in patients with gliomas before and after surgery. The study found that patients' performance in visual and auditory emotion recognition decreased after surgery but recovered 3 months after surgery. Patients with left-sided brain damage had greater deterioration in facial expression recognition following surgery than right-sided brain damage patients. The study also showed that deficits in specific expression recognition was associated with lesions in discrete locations. |
| (Motomura et al. 2018) | LGG | Japan | 2018 | Retrospective | 9 | Pre-operative, intraoperative, and 6 months post-operatively | Supratotal resection (all) | WAIS-III, WMS-R, FAB | This study aimed to evaluate surgical outcomes of awake brain mapping to achieve supratotal resection. It found that neurocognitive outcomes improved significantly after surgery in terms of WAIS-III scores for verbal IQ and verbal comprehension, and WMS-R scores for generalised memory and delayed recall. The authors conclude that this supports the role of awake surgery for supratotal resection of diffuse frontal LGGs. |
| (Motomura et al. 2019) | Diffuse frontal LGGs | Japan | 2019 | Retrospective | 50 | One week before surgery and 6 months after surgery | Surgery (all) | WAIS-III and WMS-R | This study aimed to evaluate neurocognitive and functional outcomes of patients with frontal LGGs in dominant and non-dominant hemispheres after awake brain mapping. The study found significant improvements in perceptual organisation in WAIS-III following awake craniotomy, as well as WMS-R scores for visual memory and delayed recall. No significant deterioration was observed in neurocognitive functions. |
| (Muto et al. 2018) | Low-grade glioma | France, Japan and USA | 2018 | Prospective cohort | 39 | Preoperatively (mean 24.1 ± 21.2 days before surgery) and postoperatively (mean 14.6 ± 13.2 months after surgery) | Surgery (all; total resection in 46.2%, subtotal resection in 41% and partial resection in 12.8%) | BDAE, timed picture naming task using DO80, fluency semantic and phonologic tasks, timed semantic association task, and timed reading task. Other tests were performed on a case by case basis, including for praxis (Rey figure), algebra (addition, subtraction, and multiplication tasks), memory (spatial and temporal orientation, forward and backward digital span, verbal span, free and cued selective recall task, and long-term reproduction of the Rey figure), attention (dual-task test, D2 test of attention, and bells test), and executive function (timed TMT-A and -B, timed PASAT, and timed Stroop test). | This study evaluated the effect of surgery on postoperative cognitive function and ability to work in patients with diffuse LGG. The study found that all three patients without pre-operative cognitive deficit had preserved cognitive function postoperatively. A significant inverse relationship between worsened post-operative cognitive function and extent of resection was observed. An independent interaction between extent of resection and improved postoperative cognition was also observed. |
| (Nakada et al. 2020) | WHO grade II glioma (n=14), WHO grade III glioma (n=13), WHO grade IV glioma (n=6) | Japan | 2020 | Retrospective | 33 | Before surgery and 3-months postoperatively | Surgery (all), chemoradiotherapy (n=19; WHO grade III and IV patients, inferred from text) | Visuospatial cognition (Line Bisection Task), spatial working memory (Spatial 2-Back Test) | This study evaluated the resection rate and preservation of specific cognitive functions (visuospatial cognition and spatial working memory) in 33 patients undergoing awake surgery for right frontal HGG and LGG. The study found that patients who underwent awake mapping for visuospatial cognition and spatial working memory were more likely to have intact function in these cognitive domains postoperatively. The authors concluded that awake surgery for right frontal gliomas with intraoperative mapping is more likely to result in successful preservation of visuospatial cognition and spatial working memory. |
| (Nakajima et al. 2017) | LGG (n=9), HGG (n=9) | Japan | 2017 | Prospective cohort | 18 | Preoperatively, 1 week and 3 months postoperatively | Surgery (all), chemotherapy (n=9, all HGG patients), radiotherapy (n=3, all HGG patients) | Processing speed (Letter Cancellation Test), fluency (Verbal Fluency Test), spatial working memory (Spatial 2-Back Test), emotion (Expression Recognition Test), theory of mind and social cognition (Picture Arrangement task of WAIS), visuospatial cognition (Line Bisection Test) | This study aimed to investigate the characteristics and causes of persistent cognitive dysfunction in patients with right-sided gliomas. The study found that patients with preoperative neuropsychological deficits are more likely to have ongoing deficits in the chronic phase relative to those that were normal. In addition, visuospatial dysfunction was found to commonly persist until the chronic phrase, and potentially be due to damage to the superior longitudinal fasciculus I and II. |
| (Nakajima et al. 2019) | WHO grade II glioma (n=24), WHO grade III glioma (n=19), WHO grade IV glioma (n=23) | Japan | 2019 | Retrospective | 66 | Pre-operatively, 1 week, and 3 months after surgery | Gross total resection (n=27), subtotal resection (n=11), partial resection (n=28) | Working memory (2-back test), expression recognition test, picture arrangement task of the WAIS-III, visual cognition (line bisection task), picture naming test. Other tests were performed but not analysed statistically, including: figure copying test, clock drawing test, cancellation test. | This study evaluated neurological and neuropsychological outcomes in patients that have undergone awake craniotomies. It found that almost all functions recovered within 3 months following surgery. Visuospatial cognitive deficits, however, remained in 8.2% of patients. There was no significant difference in overall neuropsychological impairments by WHO grade, but picture naming deficits at 3 months were more likely in WHO grade 4 tumours. All patients with left hemisphere lesions recovered from visuospatial cognitive deficits, whereas 14.3% of patients with right-sided lesions had persistent visuospatial cognitive deficits. Visuospatial cognitive deficits persisted at 3 months in 10.7% of patients with frontal lesions and 7.7% with parietal lesions. For working memory and mentalising, however, there were no specific lesion-based characteristics identified. Furthermore, there was no relationship between tumour biology and cognitive outcomes. |
| (Ng et al. 2020) | LGG | France | 2020 | Retrospective | 47 | The day before surgery and 3 months after surgery | Supratotal resection (n=12), total resection (n=16), subtotal resection (n=19) | Language and semantics (DO80 naming task, Pyramids and Palm Trees Test, and phonological and semantic verbal fluencies); psychomotor speed and attention (subtest “code” from the WAIS–Fourth Edition, subtests “color naming” and “reading” from the Stroop task, and TMT-A), executive functioning and working memory (TMT-B, phonological and semantic verbal fluencies, Stroop interference minus naming tasks, and forward and backward digit span from the WAIS-IV), episodic verbal memory (Rappel libre et Rappel indicé à 16 items [a French adaptation of the Free and Cued Selective Reminding Test]), visuospatial functioning (ROCFT, Taylor complex figure, Bells test, and line bisection task). | This study investigated neuropsychological outcomes in patients with incidentally found LGGs who underwent awake surgery. It found that 87.2% of patients had stable or improved neurocognitive outcomes after surgery, the remainder had a slight impairment in at least one cognitive domain. Mean pre- and postoperative z-scores were comparable except for in the psychomotor speed and attention domains. There was a significant correlation between executive functioning preoperatively and tumour volume, but extent of resection and histomolecular profile did not affect neuropsychological outcomes. |
| (Noll et al. 2015) | WHO grade II glioma (n=15), WHO grade III glioma (n=20), WHO grade IV glioma (n=37) | USA | 2015 | Not specified | 72 | Prior to surgery | None | Attention (WAIS-R/III digit span), learning and memory (HVLT-R total recall, delayed recall, and recognition discrimination index), processing speed (WAIS-R/III digit symbol, TMT A), executive function (TMT B, WAIS-R/III similarities, MAE Controlled Oral Word Association), language (MAE Token Test), visuospatial function (WAIS-R/III Block Design), motor function (Grip Strength Difference, Grooved Pegboard Difference) | This study evaluated the role of tumour grade on neurocognitive function prior to surgery or other treatments. The study found neurocognitive impairments (defined as z-scores ≥-1.5) to be commoner in patients with grade IV tumours compared to patients with lower-grade tumours in verbal learning, executive functioning, and language ability. Mean performances were significantly different according to tumour grade on measures of verbal learning, executive functioning, language and processing speed, with the patients with grade IV tumours showing worse performance than the lower-grade tumour patients. Group differences in mean performances remained significant when controlling for tumour volume determined on MRI (T1-weighted and fluid attenuated inversion recovery sequences). Performance did not differ by seizure status, anti-epileptic drugs use, or steroid use. |
| (Noll et al. 2016) | LGG (n=23), HGG (n= 80) | USA | 2016 | Prospective cohort | 103 | Prior to surgery | None | Attention (WAIS-R/III digit span), learning and memory (HVLT-R total recall, delayed recall, and recognition discrimination index), processing speed (WAIS-R/III digit symbol, TMT A), executive function (TMT B, WAIS-R/III similarities, MAE Controlled Oral Word Association), language (MAE Token Test), visuospatial function (WAIS-R/III Block Design), motor function (Grip Strength Difference, Grooved Pegboard Difference) | This study reviewed neurocognitive function according to tumour location in patients with glioma. In total, 75% of patients had impaired cognition on testing. Patients with right temporal lobe gliomas were most often impaired in verbal memory and executive functioning, at similar rates to the left temporal glioma group. Attention and object naming performance were significantly worse in the left temporal lobe glioma group. Attention, verbal learning and memory, and language were significantly worse in the left temporal lobe glioma group. Significant group-level differences in cognitive performance remained even after controlling for tumour volume on MRI and tumour histology. Overall, the study confirmed that patients with left temporal lobe tumours have more marked neurocognitive impairment across multiple domains. |
| (Noll et al. 2017) | LGG (n=23), HGG (n= 80) | USA | 2017 | Prospective cohort | 103 | Prior to surgery | None | Attention (WAIS-R/III digit span), learning and memory (HVLT-R total recall, delayed recall, and recognition discrimination index), processing speed (WAIS-R/III digit symbol, TMT A), executive function (TMT B, WAIS-R/III similarities, MAE Controlled Oral Word Association), language (MAE Token Test), visuospatial function (WAIS-R/III Block Design), motor function (Grip Strength Difference, Grooved Pegboard Difference) | This study evaluated the relationships between neurocognitive functioning, mood disturbance, and health-related quality of life in patients with brain tumours. The study found that, in patients with temporal lobe glioma, depressive symptoms were strongly related to most aspects of health-related quality of life but not neurocognitive functioning. Verbal learning and memory, executive functioning, and processing speed had direct relationships with numerous aspects of health-related quality of life. |
| (Noll et al. 2021) | WHO grade II glioma (n=8), WHO grade III glioma (n=1), WHO grade IV glioma (n=6) | USA | 2021 | Prospective cohort | 15 | Within 2 weeks prior to surgery and again within 12 weeks after surgery but before radiotherapy or chemotherapy | Gross total resection (n=7), near total resection (n=3), subtotal resection (n=5) | Attention (WAIS-IV Digit Span and Arithmetic), learning and memory (HVLT-R Total Recall, Delayed Recall and Delayed Recognition), processing speed (WAIS-IV Coding and Symbol Search, TMT-A), executive function (TMT-B, WAIS-IV Similarities, MAE Controlled Oral Word Association, Semantic [Animal] Fluency), language (MAE Token Test, BNT Naming), visuospatial function (WAIS-IV block design), clinical trial battery composite (HVLT-R, TMT, COWAT) | This study evaluated the impact of brain tumour resection on functional connectomics and the relationship with neurocognitive outcome in patients with left perisylvian gliomas. The study found most of the patients had postoperative decline on one or more neurocognitive function measures, with a significant postoperative decline in verbal memory, processing speed, executive functioning, receptive language, and a composite index. The study also found an inverse relationship between neurocognitive function and changes in network properties assessed through resting-state fMRI. |
| (Norrelgen et al. 2020) | WHO grade II astrocytoma (n=12), WHO grade III astrocytoma (n=3), WHO grade II oligodendroglioma (n=5), WHO grade III oligodendroglioma (n=2), cavernoma (n=2), ganglioglioma (n=2), glioblastoma (n=1) | Sweden | 2020 | Not specified | 27 | Preoperatively, 3 and 12 months postoperatively | Surgery (all) | Comprehension of grammar (Test for Reception of Grammar: Version 2), vocabulary and word retrieval (BNT), verbal cognitive speed (Months Backwards Test), comprehension of instructions (Token Test), high-level language functions (BESS), phonemic word fluency (FAS), sematic word fluency (Animals, Verbs), verbal cognitive speed (AQT - A Quick Test of Cognitive Speed), reading speed (DLS), vocabulary (Group Tests in Reading and Writing for Upper Secondary School) | This study evaluated high-level language outcomes 3 and 12 months after awake surgery for brain tumours and cavernomas. The results indicated that overall high-level language ability had not significantly deteriorated at the group level 3 or 12 months following awake surgery. There were 13% and 9% of patients with a decline of scores of 5% or more at 3 and 12 months, respectively. A marked decline at 3 and 12 months postoperatively was observed in semantic fluency (animals and verbs), which was the only cognitive domain to have significantly declined at 12 months relative to preoperative levels. Phonemic fluency was not significantly reduced at 3 months, but improved markedly between 3 and 12 months. Although verbal cognitive speed did not decline significantly postoperatively, 40% of patients had a decline of 5% or more at 12 months. The authors concluded that their results indicate a decline in the processing speed of verbal material postoperatively in the patient group. |
| (Owen et al. 1996) | Multiple tumour types, and non-tumour diagnosis such as intracranial aneurysms and arteriovenous malformations | UK | 1996 | Prospective cohort | 92 | Average of 4 years and 10 months after surgery (range: 3 months to 26 years) | Surgery (all) | Spatial Working Memory Task, Visual and Verbal Working Memory Tasks | Spatial working memory was most affected in the frontal lobe group and were related to the inefficient use of a search strategy known to improve performance in the task. To contrast, visual and verbal working memory were not affected in the frontal lobe group; visual but not verbal working memory was significantly impaired in the temporal lobe and amygdalo-hippocampectomy groups. |
| (Pallud & Dezamis 2017) | LGG (n=49), HGG (n=58) | France | 2017 | Prospective cohort | 107 | Prior to surgery, 3-5 days postoperatively and a mean of 12.1 ± 11.4 months. | Surgery (all), radiotherapy (n=10), chemotherapy (n=11) | BDAE, timed picture naming task using DO80, fluency semantic and phonological tasks, a timed semantic association task, and a timed reading task. In addition, depending on the tumour location and patient symptoms, additional testing was performed for example: praxis (Rey Figure), calculus (addition, subtraction, and multiplication tasks), memory (spatial and temporal orientation, forward and backward digital span, verbal span, free and cued selective recall task, long-term reproduction of the Rey figure), attention (dual-task test, D2 of the attention test, and bells test), executive function (timed TMT parts A and B, timed paced auditory serial addition test, and timed Stroop test). | This study evaluated functional and oncological outcomes in a group of patients with gliomas undergoing awake craniotomy. In 85% of patients, neurological and/or cognitive function deteriorated after surgery. However, after six months of postoperative follow-up, 89.9% of cognitive impairments improved and 30.8% of patients with a pre-operative language impairment improved (1.9% had worsening of language function). |
| (Peiffer et al. 2013) | Glioblastoma (n=7), anaplastic glioma (n=11), primitive neuroectodermal tumours (n=4), benign or low-grade tumours (n=35) | USA | 2013 | Retrospective | 57 | Variable but at least six months after radiotherapy | Radiotherapy (all) | TMT-A and -B, modified ROCFT, California or Hopkins Verbal Learning Test, Digit Span, COWAT | This modelling study aimed to assess neuroanatomical targets associated with radiation-induced cognitive decline using dose volume histogram analyses of specific brain regions of interest correlated with neurocognitive performance. The study found that brain regions predictive of global cognitive outcomes at radiation doses <60 Gy included the corpus callosum, left frontal white matter, right temporal lobe, bilateral hippocampi, subventricular zone, and cerebellum. Regions that did not predict global cognitive outcomes at any dose included total brain volume, frontal pole, anterior cingulate, right frontal white matter, and the right precentral gyrus. |
| (Peper & Irle 1997) | LGG (n=6), high-grade glioma (n=11), solitary metastases (n=4), meningiomas (n=16), others (n=3), and matched controls (n=12). | Germany | 1997 | Prospective cohort | 52 | "Approximately 8 days postoperatively" | Surgery (all) | Selection of Category Labels, Matching Emotion Categories Between Auditory and Visual Stimuli, Matching Emotion Dimensions Between Auditory and Visual Stimuli, general cognitive functioning (WAIS-IQ), visuo-perceptual functions (BVRT, Block Design, Fragmented Figure Recognition, TMT-A), and verbal-conceptual performance (Similarities, Verbal Span, Omission Errors in a paired associate learning paradigm). | The impairment of arousal and emotional valence was found after resective surgery in various brain regions, but particularly in the right temporoparietal region. |
| (Postma et al. 2002) | LGG | The Netherlands | 2002 | Prospective cohort | 39 | Median of 5 years (range 1-19) between radiotherapy and testing, or 4 years (range 1-10) from diagnosis in those who did not receive radiotherapy | Biopsy (n=15), resection (n=24), radiotherapy (n=23) | Information processing capacity (Letter-Digit Substitution Test), memory performance (Visual Verbal Learning Test), attention (Stroop Color-Word Test), and graphomotor speed (Concept Shifting Test). | Cerebral atrophy was commoner (n=14 of 23, 61%) in patients treated with prior radiotherapy than those not treated with radiotherapy (n=1 of 16, 6%). Furthermore, white matter abnormalities were detected in six patients, all of whom had received radiotherapy previously. There were significant correlations between these radiological findings and cognitive performance. |
| (Prabhu et al. 2014) | LGG | USA | 2014 | Analysis of data collected as part of an RCT | 251 | Baseline and years 1, 2, 3, and 5 | Surgery (all), radiotherapy (n=all), chemotherapy (125) | MMSE | This study evaluated the effect on cognition of adding chemotherapy (PCV; procarbazine, lomustine, and vincristine) to radiotherapy treatment in patients with WHO II glioma. Patients were randomised to one of two treatment groups (chemotherapy and radiotherapy or radiotherapy alone). The study found no significant increase in cognitive decline, as measured using the MMSE, when adding PCV chemotherapy to radiotherapy treatment during the five-year follow-up period. Few patients experienced a significant decline in MMSE scores, and both study arms experienced a significant gain in average MMSE scores over time, with no difference between the two groups. |
| (Prat-Acín et al. 2021) | WHO grade II diffuse astrocytoma (15 operated on awake with intraoperative brain mapping, 15 operated under general anaesthesia) | Spain | 2021 | Not specified | 30 | Before surgery, intraoperatively, and 6 months after surgery | Gross or near (>90%) total resection (n=7 in awake group, n=0 in control group); inferred subtotal resection (n=8 in awake group, n=15 in control group) | Number counting 1–10 to record areas of speech arrest, DO80 oral picture naming task, Pyramid and Palm Trees Test nonverbal semantic association task, simultaneous task of left arm movement and naming to check multi-tasking abilities, working memory and attention, a line bisection test to check disturbances of spatial cognition, modified version of the "Reading the Mind in the Eyes" test to check mentalizing, and a famous faces task to check face-naming process. | This study aimed to evaluate the utility of intraoperative brain mapping during awake surgery for LGGs in the right non-dominant hemisphere, comparing to a control group of patients with tumours in the same location operated on under general anaesthesia without brain mapping. The study found a higher mean extent of resection in the awake brain mapping group (86.66% versus 60.33%). Worse neuropsychological outcomes were noted in the control group, particularly in patients with tumours in parietal and insular locations. |
| (Quiñones et al. 2021) | LGG | Spain | 2021 | Case series | 5 | One week before surgery and approximately 4 months after surgery | Surgery (all) | General cognitive status (Spanish version of the MMSE), verbal and non-verbal intelligence (Spanish version of the Kaufman Brief Intelligence test), language production (Picture Naming Test) | This study evaluated clinical and imaging (fMRI, MEG) findings in 5 bilingual (Spanish-Basque) patients with LGG, exploring language reorganisation following awake surgery with brain mapping. The study found that all patients preserved linguistic function in both of their spoken languages following surgery. Reorganisation of the language network was found in both languages after surgery. |
| (Racine et al. 2015) | LGG | USA | 2015 | Retrospective | 22 | Before surgery and a median of 215 days (range 119-724 days) after surgery | Surgery (all), radiotherapy or chemotherapy (n=3) | MoCA, Grooved Pegboard, Symbol-Digit Modality Test (Written & Oral Versions), Delis-Kaplan Executive Function Scale (TMT, Design Fluency), Neuropsychological Assessment Battery (Digit Span, Visual Discrimination), California Verbal Learning Test–2nd Edition, WMS–4th Edition (Logical Memory), ROCFT (Copy and Immediate Recall Trials), Verbal Fluency (FAS and Animals), and BNT. | This study investigated preoperative baseline cognitive deficits in LGG patients and the effects of surgical resection with awake mapping. Prior to surgery, 92% of patients/caregivers reported cognitive or mood changes. Although neurological examinations and MoCA scores were largely normal, nearly half of individuals had deficits in several tests of memory and language. Most cognitive performance postoperatively was stable or improved, particularly in relation to language, although some patients declined in relation to memory tasks. |
| (Raysi Dehcordi et al. 2013) | LGG (n=15), HGG (n=27) | Italy | 2013 | Not specified | 42 | Preoperatively, six months, and one year after surgery | Surgical resection (all) | RCPM, 15 Rey's Words Test, Digit Span, TMT-A and -B, Fluency Verbal Test, Tower of London Test, ROCFT, Copying Drawings of Geometric Figures, Visual Memory, Test of Selective Attention, Denomination. | This study compared pre- and post-operative cognitive functioning in patients with HGG and LGG. The study found that memory and processing speed improved postoperatively in both HGG and LGG patients. Lesion size, oedema, and laterality of the tumour all influenced cognitive outcomes. |
| (Reijneveld et al. 2001) | Suspected LGG (n=24), proven LGG (n=24) | The Netherlands | 2001 | Prospective cohort | 48 | Suspected LGG group: mean 4.4 ± 4.5 years from diagnosis to testing; proven LGG group: mean 5.5 ± 3.7 years from diagnosis to testing | Suspected LGG group: none; proven LGG group: biopsy (n=6), surgical resection (n=17), unknown (n=1) | Visual Verbal Learning Test, Working Memory Task, Letter–Digit Substitution Test, Categoric Word Fluency, Concept Shifting Test, Stroop Color Word Test, and Working Memory Task | This study compared functional status, quality of life, and cognitive function in patients suspected of having a LGG but not receiving treatment, and patients who underwent early surgery that were confirmed to have LGG. Cognitive status was worse in both the suspected and proven LGG groups relative to healthy controls, but those with suspected LGG that did not receive treatment performed better than the proven LGG group that did. The authors concluded that a conservative management approach to patients suspected of having LGG did not negatively affect cognition. |
| (Rijnen et al. 2019) | WHO grade II glioma (astrocytoma or oligodendroglioma; n=77) | The Netherlands | 2020 | Prospective cohort | 77 | Baseline (pre-operative) and 3 months post-operatively | Surgery (all), radiotherapy without chemotherapy (n=32), radiotherapy and chemotherapy (n=6) | Verbal Memory Test, Visual Memory Test, Finger Tapping Test, Symbol Digit Coding Test, Stroop Test, Shifting Attention Test, Continuous Performance Test, Letter Fluency Test | This study evaluated the cognitive performance of patients with LGG before and after surgery, specifically evaluating the effects of hemispheric tumour location and surgery type (with or without intraoperative stimulation mapping). Cognitive impairments were found in patients irrespective of the hemispheric location of their tumour. Patients who were operated on with the use of intraoperative stimulation mapping performed slightly worse on cognitive tests after surgery than those who had surgery without mapping. |
| (Robinson et al. 2015) | LGG (n=9), HGG (n=10), meningioma (n=4) | Australia | 2015 | Prospective cohort | 23 | Mean of 2.1 months (SD 3.1) after surgery | Surgery (all) | Cognitive screening (MoCA), pre-morbid IQ (NART), abstract reasoning, non-verbal (Raven’s Advanced Progressive Matrices), verbal (Proverb Interpretation Test), attention (Digit Span subtest from the WAIS-III, Elevator Counting with Distraction from the Test of Everyday Attention), verbal and visual memory (Recognition Memory Tests, Words, and Topography), visual perception (Incomplete Letters Test from the Visual Object and Space Perception Battery), language (Graded Naming Test, Word Comprehension – Synonyms Test), and executive functions (Phonemic Word Fluency, HSCT). | This study aimed to evaluate the sensitivity of the MoCA screening tool compared to a brief but tailored cognitive assessment in identifying cognitive deficits in patients with brain tumours. The study found that those considered impaired with the MoCA had lower naming and word fluency score than those considered intact with the MoCA, but test scores between the two groups were broadly comparable. This indicates that the MoCA is overall of poor sensitivity in detecting cognitive impairments, and a more detailed cognitive assessment is needed in these patients. |
| (Roman-Goldstein et al. 1995) | CNS lymphoma (n=9), germ cell tumour (n=3), astrocytoma (n=2), primitive neuroectodermal tumour (n=1) | Canada, Australia, USA | 1995 | Prospective | 15 | Baseline (24-48 hours before initial BBB disruption procedure) and after completion of 1 year of therapy and one yearly intervals thereafter. | Chemotherapy (all) | WAIS-R, WMS-R, TMT-A and -B, California Verbal Learning Test, ROCFT, Finger Tapping Speed, and Grip Strength. In children less than 8 years of age, the Stanford Binet Intelligence Scales (4th ed) was the only test administered. For children between 9 and 16 years of age, the Wechsler Intelligence Scale for Children–Revised, was substituted for the Wechsler Adult Intelligence Scale of Learning and Memory–Revised, the Wide Range Assessment of Learning and Memory was substituted for the Wechsler Memory Scale–Revised, the California Verbal Learning Test was omitted, and the age-appropriate version of the TMT was administered. | This study evaluated whether osmotic BBB disruption is associated with MRI abnormalities or cognitive impairment in patients with brain tumours, and the potential correlations between MRI and cognitive function. The study found no cognitive deterioration following treatment with osmotic BBB disruption and intraarterial chemotherapy. Some of the subjects developed MRI abnormalities but these did not correlate with cognitive test results. |
| (Romero-Garcia et al. 2021) | WHO grade I glioma (n=2), WHO grade II glioma (n=7), WHO grade III glioma (n=5), WHO grade IV glioma (n=3) | UK | 2021 | Prospective cohort | 17 | Before surgery (n=17), after surgery (n=8), after 3 months (n=11), after 12 months (n=11) | Complete resection (n=9), partial resection (n=8), adjuvant chemoradiotherapy (n=12) | Memory (free verbal memory, overall verbal memory, episodic memory, orientation, forward digit span, backward digit span) | This study aimed to evaluate brain tumour interactions with brain networks to identify protective features and risk factors for memory recovery after surgery for diffuse nonenhancing glioma. Resection and radiation treatment had an impact on memory in most patients. Memory recovery after surgery showed various trajectories, including progressive impairment, impairment followed by recovery, no change, and improvement after surgery and during recovery. Years of education showed no relationship with preoperative memory performance or with memory recovery. Follow-up recovery was not associated with tumour location or the type of treatment received by patients (surgery vs surgery and adjuvant chemoradiotherapy). Tumour volume before surgery and after surgery was not correlated with memory recovery. Brain tumours and associated treatments can affect brain networks essential for memory and influence memory recovery following treatment. |
| (Rossi et al. 2019) | LGG (n=357), HGG (n=92) | Italy | 2019 | Prospective cohort | 449 | Pre-operatively, 5 days, and 1-3 months after surgery (additional neuropsychological data at 1 year were available in 60 patients) | Surgery (all) | Milano-Bicocca Battery (testing language, memory, praxis abilities, attentive and executive functions, and fluid intelligence). | This study aimed to evaluate the feasibility and functional outcomes following supratotal resection of presumed LGGs (based on radiological appearances), and provided neuropsychological outcomes in a subgroup (n=100) of the study. Extent of resection (total vs supratotal) did not significantly affect memory, language, or fluid intelligence scores, but across the whole group of patients a decrease in mean test scores immediately after surgery was observed, followed by a recovery after 3 months. Praxis was better immediately after surgery in the total resection group relative to the supratotal resection group, but this difference reversed after 3 months. Those in the supratotal group experienced a better recovery of executive functions. Cognitive profiles were not influenced by previous surgery or molecular profiles. Female gender was associated with a better language performance immediately after surgery, as well as a faster recovery, compared to males, but scores were comparable at 1 year follow-up. |
| (Rossi et al. 2021) | HGG (n=26), LGG (n=69) | Italy | 2021 | Retrospective | 95 | Before surgery, and 5 days, 1–3 months, and 1 year after surgery | Gross total resection (n=70), partial or subtotal resection (n=25) | Milano-Bicocca Battery, assessing five neurocognitive domains: language, memory, visuospatial abilities and praxis, attentive and executive functions, and fluid-intelligence | This study evaluated the neurosurgical outcomes and functional effect of resection of giant insular gliomas, with a focus on neuropsychological and quality of life outcome measures. The study found 50.5% of patients had pathological scores in at least one cognitive domain preoperatively, most commonly memory (48.4%) and language (33.3%). Five days after surgery, most patients declined in at least one of the cognitive domains analysed, but substantial recovery was observed in all domains at 3 months postoperatively, but language, attention, and executive functions were the domains that exhibited the slowest recovery rate. At one year following surgery, only 6.3% of patients had new-onset language deficits, all relating to speech production (naming ± verbal fluency), and three also in comprehension. The study found that extensive intraoperative mapping for cognitive, visual and haptic functions decreased long-term neurological, neuropsychological and quality of life morbidity and also increase the extent of resection. Ischaemic insults in eloquent brain regions were found to be the leading factor associated with long-term neurological and neuropsychological morbidity. |
| (Sanai et al. 2008) | LGG (n=124), HGG (n=126) | USA | 2008 | Retrospective | 250 | Preoperatively, one week after surgery, and six months after surgery | Surgery (all) | Language testing (counting from 1-50, naming objects pictured on a computer-generated slide show, reading single words projected sequentially on a computer screen, repeating complex sentences, and writing words and sentences on paper). | This study evaluated language function after surgical resection of gliomas with awake language mapping. The study found that 159 (63.6%) patients had intact speech prior to surgery. One week after surgery, baseline language function remained stable in 194 (77.6%) patients; it worsened in 21 (8.4%) patients and new speech deficits developed in 35 (14.0%) patients. At six months following surgery, only 1.6% of surviving patients had a language deficit. Language localisation was found to vary widely in the dominant hemisphere between subjects. |
| (Santini et al. 2012) | LGG (n=14), HGG (n=8) | Italy | 2012 | Prospective cohort | 22 | Pre-operatively, post-operatively (timing not specified), and 3-6 months following surgery | Total surgical resection (n=17), subtotal surgical resection (n=5) | Intelligence (Raven Colored Matrix), executive functions (Word Fluency/FAS, TMT-A and -B), memory (Verbal Digit Span, 15 Rey Word List-immediate and delayed recall, ROCFT), language (Picture Object Naming, BASA subtests of language), praxis (Copy Design, Limb Praxis, Orofacial Praxis), gnosis (Body Part Naming, Finger Naming), depression (Beck Depression Inventory), anxiety (STAI) | This study evaluated the relationships between tumour variables, mood, cognition, and language. Prior to surgery, 45% of patients were depressed and 23% were anxious. Cognitive impairment was noted in 59% and language impairment in 50%. Following surgery, a general decline in cognitive performance was observed, that was statistically significant for memory and attention only. Similarly, language function declined, in a statistically significant manner for picture naming. Depression was unchanged and anxiety improved after surgery. Tumour histology but not demographic variables or extent of resection correlated with postoperative cognitive changes, with an improvement in cognitive performance most likely in the high-grade tumour group. There were no correlations between mood, cognition, and language function. In the subset of LGG patients followed-up at 3–6 months following surgery, some improvement was noted but not always to the preoperative levels. |
| (Sarubbo et al. 2011) | LGG (all) | Italy | 2011 | Not specified | 12 | Pre- and post-operatively (specific time not specified) | Surgery (all) | A range of tests were used depending on the tumour location. The commonest tests used were: general evaluation of cognitive functions (MMSE), visual naming test for anomia (Laiacona-Capitani Naming Test), understanding evaluation (Token Test). | This study evaluated the outcomes following surgery for awake craniotomy for LGG. It found no change in cognitive status following surgery. |
| (Satoer et al. 2014) | HGG (n=17), LGG (n=27), unknown (n=1) | The Netherlands | 2014 | Not specified | 45 | Pre-operatively (mean 1.4 months, SD 1.06), approximately 3 months (mean 3.4 months, SD 0.72) and 1 year (mean 1.01 years, SD 0.16) postoperatively. | Biopsy (n=4), partial resection (n=26), subtotal resection (n=7), total resection (n=2), chemotherapy (n=4), radiotherapy (n=19), chemotherapy and radiotherapy (n=6) | Language (subtests of AAT - Token Test/Repetition/Reading Aloud/Writing to Dictation, BNT, Category Fluency, Letter Fluency-parallel versions), memory (15 word test, encoding, recall, recognition-parallel versions), attention and executive functions (TMT-A, -B and BA) | Impairments were detected in all cognitive domains pre- and post-operatively (language, memory, attention, and executive functions). After surgery, permanent improvement was observed in a memory test (verbal recall) and deterioration was found in a language test (category fluency). Between 3 months and 1 year, improvement was observed in two language tests (naming and letter fluency). Tumour- or treatment-related factors did not influence cognitive changes. The authors concluded that the results highlight the importance of longer-term cognitive testing, given that cognitive recovery took longer than 3 months especially within the language domain. |
| (Satoer et al. 2017) | LGG (n=2), HGG (n=2) | Belgium and the Netherlands | 2017 | Case series | 4 | Preoperatively and either (a) six weeks and six months postoperatively, or (b) three months and one year postoperatively | Surgery (all), radiotherapy (n=3) | Language (BNT or object naming [DuLIP], action naming, category and letter fluency, and AAT subtests: repetition, writing to dictation, reading aloud, and Token Test), memory (15- word test-imprinting, recall, digit span), attentional and executive function (design fluency, TMT-A and -B, Stroop Color Word Tests I–III). | This study compared the cognitive outcomes in patients with HGG and LGG that underwent awake craniotomy for tumours in critical language areas (Broca, Wernicke, and arcuate fasciculus). The study found that not all patients with tumours in areas of the brain deemed 'critical' for language have cognitive disturbance, and surgery improved or worsened existing cognitive impairments. |
| (Satoer et al. 2018) | LGG (n=11), HGG (n=7) | The Netherlands | 2018 | Prospective longitudinal study | 18 | Pre-operatively, 3 months and 1 year after surgery. | Surgery (all) | TTR, MLUw, Incomplete Sentences, Self-corrections and Repetitions, BNT and CF | This study evaluated the longer-term effects of surgery on spontaneous speech in patients with LGG and HGG. The study found deficits in Incomplete sentence at all time points (pre-operatively, 3 months and 1 year postoperatively), TTR at 3 months and 1 year postoperatively, MLUw at 1 year postoperatively, and Self-corrections at 3 months postoperatively. There was no decline observed between pre-operatively and 3 months postoperatively. However, deficits in MLUw, Self-corrections, Repetitions and Incomplete sentences were observed between both 3 and 1 year postoperatively and between preoperatively and 1 year postoperatively. BNT and CF were impaired at all timepoints without differences between test-moments. Tumour location and grade did not influence spontaneous speech. As the study indicated that surgery deteriorated the quality of spontaneous speech long-term without impairing performance at test level, the authors concluded that spontaneous speech has additional value over and above standardised tests for the diagnosis of language impairments. |
| (Scarone et al. 2009) | LGG | France | 2009 | Retrospective | 15 | Language modalities were tested within the first 48 postoperative hours and at 3 months. Writing was evaluated in the immediate postoperative period, at 3 months, then every 6 months after surgery. | Surgery (all) | Language testing (auditory comprehension, counting, object naming, and spontaneous speech) through complete neurological examination, BDAE (in 11 patients), and writing testing. | This study aimed to evaluate the functional areas involved in the writing network in 15 patients that had surgery for LGG. Only patients with lesions of the supplementary motor area did not recover from agraphia during post-operative follow-up, in 50% of cases. |
| (Shankar & Rajshekhar 2003) | LGG (all WHO grade II astrocytoma) | India | 2003 | Mixed prospective and retrospective | 30 | Not specified | Biopsy (all), radiotherapy (all), chemotherapy (2 patients but unclear how many of these were in the group with cognitive testing completed) | Memory (Post Graduate Institute scale), language (Expressive Speech, Receptive Speech, Reading and Writing scales of the Luria-Nebraska Neuropsychological Battery | This study evaluated the radiological and clinical outcomes of patients with insular LGGs. Of the 11 patients (out of 30) that underwent cognitive testing, six had a slight improvement in language, whereas three worsened and two remained the same. Regarding memory function, 7 showed an improvement and 4 deteriorated. There was a non-significant trend for improved memory and language during follow-up. |
| (Sherman et al. 2015) | LGG | USA | 2015 | Prospective cohort | 20 | Baseline (within 8 weeks of initiating proton radiation therapy) and at yearly intervals for 5 years | Gross total resection (n=4), subtotal resection (n=12), biopsy (n=4), radiotherapy (all) | WAIS-III, BNT, Auditory Naming Test, Conners' CPT, Wechsler Memory Scale, TMT A and B, COWAT, WCST, HVLT, Brief Visuospatial Memory Test | This study evaluated the effect of proton radiation therapy in adults with low-grade glioma. It found overall stability in cognitive functioning, and tumour laterality was important; patients with left hemisphere tumours were more impaired in verbal measures at baseline, but there was greater improvement in verbal memory over time in patients with left hemisphere tumours compared to right hemisphere tumours. |
| (Shih et al. 2015) | LGG | USA | 2015 | Prospective cohort | 20 | Baseline (within 8 weeks of initiating radiation therapy), and at 6 months, 12 months, 24 months, 36 months, 48 months, and 60 months. | Gross total resection (n=4), subtotal resection (n=12), biopsy (n=4), radiotherapy (all) | WAIS-III, BNT, Auditory Naming Test, CPT, WMS, TMT-A and -B, COWAT, WCST, HVLT, Brief Visuospatial Memory Test | This study evaluated the potential treatment toxicity in patients with LGG who received proton radiation therapy treatment. The study found that intellectual functioning was within normal range for the group at baseline and remained stable over time. In addition, visuospatial ability, attention/working memory, and executive functioning were within normal limits, but baseline neurocognitive impairments were identified in language, memory, and processing speed in 8 of the 20 patients included in the study. There was no overall decline in cognitive functioning over time. |
| (Sunyach et al. 2003) | Oligodendrogliomas or oligoastrocytomas (WHO grade III, n=18; WHO grade II, n=8) | France | 2003 | Not specified | 26 | Median of 65 months | Incomplete surgical resection (all), conformal radiotherapy (n=12), chemotherapy followed by conformal radiotherapy (n=4), chemotherapy followed by delayed conformal radiotherapy at the time of tumour progression (n=10) | MMSE | This study described a single-centre's experience with use of conformal radiotherapy in the treatment of patients with oligodendroglioma or oligoastrocytoma, and associated outcomes. In the 11 (out of 26) participants who were disease free at the time of analysis underwent cognitive testing, it found mean MMSE scores of 27 out of 30 (range 20-30). |
| (Surma-aho et al. 2001) | LGG | Finland | 2001 | Prospective cohort | 51 | Mean of 7 years (range 3-16) in those who received radiotherapy, and mean of 10 years (range 2-16) in those who did not | Surgery (all), radiotherapy (n=28), chemotherapy - teniposide and lomustine (n=2). | WAIS (Digit Span, Similarities, Block Design, Digit Symbol subtests), Modified BVRT | Those who had radiotherapy (n=28) were compared to those that did not (n=23). Cognitive performance was significantly worse in the group of patients that had postoperative radiotherapy, and was not accounted for by factors such as histologic diagnosis, location/extent of resection/progression of the tumour, or patient factors. White matter abnormalities on imaging were more severe in the radiotherapy group, and such changes were only correlated with poor memory performances in the radiotherapy group. |
| (Tabrizi et al. 2019) | LGG | USA | 2019 | Prospective cohort | 20 | Baseline and 6, 12, 24, 36, 48, and 60 months following proton radiotherapy. | Biopsy (n=4), subtotal resection (n=12), gross total resection (n=4), proton radiotherapy (all), chemotherapy (n=2) | Not all specified, but tests covering eight cognitive domains: intellectual functioning, visuospatial ability, attention and working memory, processing speed, executive function, language, verbal memory, visual memory, and tests from the Clinical Trial Battery composite. | This study evaluated the effects of proton radiotherapy in patients with LGG. It found that the majority of patients maintained stable neurocognitive function over a median follow-up of 36 months (range: 0-60 months) following proton radiotherapy, and some improved relative to baseline. |
| (Talacchi et al. 2011) | HGG (n=17), LGG (n=12) | Italy | 2011 | Prospective cohort | 29 | Before surgery and 2-3 months after surgery | Surgery (all) | Intelligence (Raven Coloured Matrix), executive functions (Word Fluency-FAS, TMT-A and -B), memory (Verbal Digit Span, 15 Rey-Osterrieth Word List - immediate and delayed recall, Spatial Supraspan Learning, ROCFT), language (visual object naming), praxis (Copy Design, Limb Praxis, Orofacial Praxis), gnosis (Body Part Denomination, Finger Denomination), depression (Beck Depression Inventory), anxiety (STAI). | This study aimed to evaluate the effects of the tumour and surgery on cognitive outcomes without the effects of treatment such as radiotherapy and chemotherapy, by not including patients who had received these treatments. The study found that at baseline, using test- and domain-based criteria, 79% and 38% of patients had cognitive impairment, respectively; the former related to tumour factors including oedema, larger tumour volume, and higher tumour grade. The most commonly affected functions were verbal memory, visuospatial memory, and word fluency, which were partly associated with depression. Some patients remained cognitively unchanged following surgery, whereas others improved and others declined. Some participants remained intact. The EOR did not influence outcomes. Improvement of previously impaired functions correlated with high-grade tumours, and worsening in executive functions was related to tumour size and partly explained by post-operative MRI findings. |
| (Taphoorn et al. 1994) | LGG patients that received radiotherapy (n=20), LGG patients that did not receive radiotherapy (n=21), controls - patients with haematological malignancies without CNS involvement (n=19) | The Netherlands | 1994 | Prospective cohort | 60 | Mean of 3.5 years (range 1-12 years) between diagnosis and testing | Biopsy (10 in the LGG radiotherapy group, 6 in the LGG no-radiotherapy group), subtotal surgical resection (8 in the LGG radiotherapy group, 12 in the LGG no-radiotherapy group), gross total surgical resection (2 in the LGG radiotherapy group, 3 in the LGG no-radiotherapy group). | Stroop Color-Word Test, a series of mazes derived from the Wechsler Intelligence Scale for Children, RAVLT, a Categoric Fluency Task, d2-test AKA Concentration Endurance Test, Benton Facial Recognition Test, Judgment of Line Orientation test. | This study evaluated the impact of radiotherapy on patients with LGG. Th study found that patients with LGG that received and did not receive radiotherapy were significantly more cognitively affected than the controls with haematological malignancy. Furthermore, there was no significant difference in cognitive outcomes between the LGG patients that did and did not receive radiotherapy. |
| (Teixidor et al. 2007) | LGG | Spain, France | 2007 | Not specified | 23 | Preoperatively, immediately post-operatively, and at 3 months after surgery | Surgery (all) | Picture naming test - DO80, BDAE - French adaptation 1982 | This study evaluated verbal working memory in patients with LGG located in language areas of the brain. Prior to surgery, 91% of patients had verbal working memory disorders; immediately after surgery, 96% of patients experienced a worsening of verbal working memory. At 3 months, among the 8 patients that were examined, 5 recovered their preoperative verbal working memory score and 3 significantly improved it. |
| (Tomasino et al. 2020) | HGG (n=14), LGG (n=26), metastases (n=3), cavernoma (n=4), pilocytic astrocytoma (n=1), DNET (n=1) | Italy | 2020 | Not specified | 49 | Before surgery, during surgery, immediately after surgery, and 4 months postoperatively | Surgical resection (all) | Real-time Neuropsychological Testing, with the specific cognitive tasks determined on the functional role of the area involved in the surgical resection as well as the individuals' preoperative neuropsychological profile | The aim of this study was to evaluate how reading abilities change during and after surgery in patients with brain tumours. In a subset of 33 patients, 4-month follow-up data on reading ability were obtained. Impaired reading performance was seen in 7 patients prior to surgery, during surgery in 18 individuals, immediately after surgery in 26 individuals and at follow-up in 5 individuals. |
| (van Dellen, de Witt Hamer, et al. 2012) | LGG | USA and the Netherlands | 2012 | Prospective cohort | 10 | Prior to surgery and a mean of 16 weeks (range 11-25 weeks) following surgery | Surgery alone (all) | Concept Shifting Test, Categoric Word Fluency Task, RAVLT, Stroop Color-Word Test, Memory Comparison Test, Letter-Digit Substitution Test | Increased alpha band resting-state network functional connectivity detected on magnetoencephalographic recordings was found to correlate with improved cognitive outcome after resection surgery. |
| (van Dellen, Douw, et al. 2012) | LGG (n=13), HGG (n=12), non-glial lesions (n=10), and healthy controls (n=36) | The Netherlands | 2012 | Prospective cohort | 71 | Prior to surgery (duration of epilepsy: average of 44 months [SD 64 months] in the LGG group, 20 months [SD 39 months] in the HGG group, and 228 months [SD 141 months] in the non-glioma group). | None (all patients were pre-operative at the time of testing) | Attention, executive functioning, mental flexibility, mental processing speed (Stroop Color-Word Test), executive functioning (Categoric Verbal Fluency), storage and retrieval of verbal memory (Visual Verbal Learning Test) | The study compared functional networks in patients with LGG, HGG, non-glial lesions, and healthy controls using magnetoencephalography. The study revealed that network characteristics correlated with clinical presentation in relation to seizure frequency in LGG patients, and with poorer cognitive performance in both LGG and HGG patients. Decreased synchronisability and decreased global integration in the theta band were associated with the occurrence of seizures and cognitive decline. |
| (van Dokkum et al. 2019) | Diffuse LGG in language-related regions | France | 2019 | Not specified | 39 | Prior to and 3 months after surgery | Surgery (all) | D080 picture naming task | This study aimed to evaluate the relationship between resting state network plasticity and picture naming in patients with LGG before and after awake resection. Picture naming performance scores were available at both time points for 34 of the participants. Of these 34 participants, 16 had a decreased performance following surgery, 11 improved and 7 had no change in performance after surgery. |
| (van Kessel et al. 2019) | WHO grade II glioma (n=116), WHO grade III glioma (n=94), WHO grade IV glioma (n=570) | The Netherlands | 2019 | Retrospective | 780 | Before antitumour treatment | None | Attention and executive functioning (WAIS-III digit span forward, TMT switching ratio [TMT-B/TMT-A], phonologic fluency, Stroop/Delis Kaplan Executive Function System Inhibition Ratio), memory (WAIS-III Digit Span Backward, RAVLT-Dutch version immediate, delay, recognition, ROCFT delay, Semantic Fluency), language (BNT, Token Test short version of AAT), visuospatial functioning (Benton Judgment of Line Orientation, ROCFT direct), psychomotor speed (Stroop/Delis Kaplan Executive Function System I, Stroop/Delis Kaplan Executive Function System II, TMT-A) | This study aimed to evaluate neurocognitive function in patients with glioma prior to treatment, and to investigate the factors that influence neurocognitive function. Of 780 patients, 168 had complete neurocognitive testing data for analysis. All cognitive domains were significantly impaired at the group level and the most affected domains were executive functioning (26.5%), psychomotor speed (23.2%), and memory (19.3%). Patients with HGG were more affected than those with LGG. Domain-specific impairments were observed in association with tumour volume, IDH status, WHO grade, and histology. |
| (van Kessel et al. 2022) | WHO grade II glioma (n=69), WHO grade III glioma (n=33), WHO grade IV glioma (n=95) | The Netherlands, Belgium | 2022 | Retrospective | 197 | Before treatment | None | Attention and executive functioning (WAIS-III digit span forward, TMT switching ratio [TMT-B/TMT-A], phonologic fluency, Stroop/Delis Kaplan Executive Function System Inhibition Ratio), memory (WAIS-III Digit Span Backward, RAVLT-Dutch version immediate, delay, recognition, ROCFT delay, Semantic Fluency), psychomotor speed (Stroop/DKEFS I, Stroop/DKEFS II, TMT-A) | This study evaluated the relationship between cognitive performance (executive function, memory, and psychomotor speed) and the intratumoural expression levels of molecular markers in patients with diffuse glioma prior to treatment. After correction of tumour volume and location, significant associations were identified as follows: between expression levels of CD3 and IDH-1 and psychomotor speed; between IDH-1, ATRX, NLGN3, BDNF, CK2Beta, EAAT1, GAT-3, SRF, and memory performance; and between IDH-1, P-STAT5b, NLGN3, CK2Beta, and executive functioning. There were also independent assocations identified between P-STAT5b, CD163, CD3 and Semaphorin-3A after correcting for histopathological grade. The authors concluded that variations in glioma biology can influence cognitive function through mechanisms that include disturbed neuronal communication. |
| (Vendrell et al. 1995) | Multiple tumour types (n=13), traumatic brain injury (n=13), vascular lesions (n=5), abscess (n=1), and matched controls (n=32) | USA | 1995 | Prospective cohort | 64 | Mean 28.91 months after onset or surgery (range: 2 - 112 months) | Surgery (n=24) | Stroop test | Significant differences were found between patients and controls in errors but not reaction time. The region most related to errors was the right prefrontal lateral cortex. Left-sided lobectomies did not impair Stroop performance. The study results indicate the role of the right prefrontal cortex in sustained attention and go against the idea that the left prefrontal cortex has a role in the inhibition of verbal automatic responses. |
| (Vilkki et al. 2002) | LGG (n=23), high-grade glioma (n=10), but also other pathologies including arteriovenous malformation (n=8) and other (n=6). | Finland | 2002 | Prospective cohort | 47 | Between one and four months after surgery | Surgery (all), radiotherapy (n=10) | WAIS-R (Digit Span, Similarities, and Block Design subtests), Letter Fluency, Repetitive Pattern Drawing, Figural Fluency, and two dual-task combinations of these. | Performance in letter fluency testing was worse in those with left-hemisphere lesions than right-hemisphere lesions. |
| (Vogt et al. 2018) | WHO I DNET, n=28; WHO I ganglioglioma, n=95; WHO I pilocytic astrocytoma, n=24; WHO II pleomorphic xanthoastrocytoma, n=9; WHO II diffuse astrocytoma, n=10 | Germany | 2018 | Retrospective | 166 | Pre-operative and one year after surgery | Surgery (lesionectomy, n=108; extended resection, n=58) | Premorbid crystallized IQ (Mehrfachwahl-Wortschatz-Test, MWT-B), language functions (Token Test and a phonemic verbal fluency task), verbal learning and memory (Verbaler Lernund Merkfähigkeitstest, VLMT), nonverbal memory (revised five-trial version of the Diagnosticum für Cerebralschädigung, DCS-R), executive functions (a response inhibition task of the cerebraler Insuffizienztest, c.IT., and the digit span backwards task) | This study aimed to evaluate cognitive and epilepsy-related features in patients with long-term epilepsy-associated tumours of the temporal lobe. The study found memory to be impaired preoperatively in 67.1%, executive functions in 44.7%, and language in 45.5%. Of the pathologies included in the study, those with pilocytic astrocytomas had the worst cognitive performance. Significant memory declines observed in 27.1% of all patients were predicted by left-sided surgery, mesial pathology, and the extended hippocampal resection. Executive functions were influenced by the load of antiepileptic drugs and either remained stable (in 72% of patients) or improved (in 21.6%) after surgery. Of note, language functions were unchanged in 89.5% of patients. The authors concluded that cognitive impairments are common in patients with lesions of the temporal lobe, and that different tumour types have different cognitive outcomes, with astrocytoma patients as the group least likely to benefit from surgery. |
| (Wang et al. 2021) | WHO grade II (n=39), WHO grade III (n=30), WHO grade IV (n=31), other gliomas (n=15), lung metastases (n=20), breast metastases (n=8), renal metastases (n=3), metastases from other cancers (n=19) | China | 2021 | Prospective longitudinal study | 165 | Preoperatively, 7 days, 1 month, 3 months, 6 months, and 1 year after surgery | Gross total resection (n=132), subtotal resection (n=11), biopsy (n=22) | MMSE, MoCA, as well quality of life and activities of daily living measures | This study evaluated quality of life, activities of daily living, and cognitive function in patients with primary and metastatic brain tumours to investigate the correlation between them and the prognosis of patients. The worst performance in the tests were observed at day 7 following surgery, and recovered 1 month following surgery. Patients with left-sided tumours had significantly worse cognitive function before surgery and 7 days, 1 month, and 6 months after surgery (but not at 1 year). |
| (Wefel et al. 2016) | Glioblastoma (n=52), anaplastic astrocytoma (n=67) | USA | 2016 | Not specified | 119 | Prior to surgery | None | Attention (WAIS-R/III digit span), learning and memory (HVLT-R total recall, delayed recall, and recognition discrimination index), processing speed (WAIS-R/III digit symbol, TMT A), executive function (TMT B, WAIS-R/III similarities, MAE Controlled Oral Word Association), language (MAE Token Test), visuospatial function (WAIS-R/III Block Design), motor function (Grip Strength Difference, Grooved Pegboard Difference) | This study evaluated the role of IDH1 genetic mutation status in 119 patients with malignant gliomas prior to surgical resection. The study found neurocognitive impairment (defined as z-scores ≥-1.5) to be significantly more common in patients with IDH1 wild type tumours in memory, processing speed, visuoconstruction, language, executive functioning, and manual dexterity. Mean performances were significantly lower in patients with IDH1 wild type tumours in measures of learning and memory, processing speed, language, executive functioning, and dexterity, relative to those with IDH1 mutant tumours. There was no significant difference in lesion volume between the wild type and mutant groups. Tumour and lesion volume (on T1-weighted and fluid attenuated inversion recovery MRI sequences) were significantly inversely associated with neurocognitive function tests in patients with IDH1 wild type tumours, but only with a single measure in those with IDH1 mutant tumours. |
| (Weniger & Irle 2002) | Tumour types not specified. The study group comprised of patients following surgery for tumours (n=45) or intracerebral haemorrhage (n=23), as well as a clinical control group (n=16) and healthy control subjects (n=15). | Germany | 2002 | Prospective cohort | 99 | Not specified | Surgery (all tumour patients, 17 intracerebral haemorrhage patients) | German version of WAIS-R (Information, Similarities, Block Design, and Picture Completion subtests), Wechsler Memory Scale - Revised (Logical Memory I and II subtests and Verbal and Visual Span subtests), TMT A and B, Benton Facial Recognition Test | Study subjects were divided into groups according to the location of the lesion. Deficits in recognizing emotional facial expression were identified in the group that underwent surgical removal of brain tumours involving both heteromodal and limbic/paralimbic cortices. |
| (Wolf et al. 2016) | LGG (n=5), HGG (n=16), ependymoma WHO grade III (n=1) | Germany | 2016 | Prospective cohort | 22 | One day before surgery, on the day of discharge (7 ± 2 days), and 3 months following surgery. | Surgery (all), chemotherapy and/or radiotherapy (n=10) | NIHSS, Addenbrooke's Cognitive Examination-Revised, ZN Backwards (from WMS-R), VLMT (A) Learning Performance, VLMT 30 (Delayed Recall), VLMT (Correct Recognition), FG (from WMS-R), ZST (from HAWIE-R), TMT-B | This study evaluated the effects of neurological and neuropsychological changes following glioma surgery on quality of life. The study found that 5/22 patients deteriorated and 522 patients improved neurologically surgery. Depending on the specific test chosen, up to 57.1% of patients experienced deterioration in some neuropsychological domain, but most recovered during the three-month observation period. There was no correlation between quality of life and a patient's neurological or neuropsychological status. |
| (Wu et al. 2011) | LGG (n=18 in insular tumour group, n=14 in control tumour group), HGG (n=15 in insular tumour group, n=19 in control tumour group) | USA | 2011 | Prospective cohort | 66 | Before and after surgery (median time to postoperative assessment was 44 days [range 8-205 days] in the insular tumour group and 54 days [range 8-126 days] in the control group; this was not significantly different. | Surgery (all), chemotherapy (n=4 in both the insular and other tumour groups), radiotherapy (n=4 in the insular group and n=9 in the control group) | Attention (WAIS-III Digit Span), processing speed (WAIS-III Digit Symbol, TMT part A), executive function (TMT-B, WAIS-III Similarities, COWAT), learning and memory (HVLT-R), language (BNT or MAE Visual Naming, MAE Token Test), visuoconstruction (WAIS-III Block Design), motor (Grip Strength, Grooved Pegboard). | This study compared neurocognitive function in patients with insular gliomas compared to gliomas in nearby brain regions. The authors found that preoperative neurocognitive impairment was common in both insular gliomas and gliomas located elsewhere. Patients with insular gliomas had significantly worse preoperative performance on naming tests. In both tumour groups, a decline in postoperative cognition occurred in most domains, with no statistically significant difference in rates of decline between the two tumour groups. However, there was a trend for patients with insular gliomas to have a greater decline in learning and memory. |
| (Yavas et al. 2012) | LGG | Turkey | 2012 | Prospective cohort | 43 | At baseline (after surgery before radiotherapy), at the end of radiotherapy, and during follow-up (every 3 months for the first 2 years, and every 6 months between 2 and 5 years) | Gross total resection (n=10), subtotal resection (n=28), radiotherapy (all) | MMSE, EORTC QLQ-C30 (contains one cognitive domain) | This study evaluated quality of life in patients treated for LGG. It found cognitive function scores at 3 years were significantly worse for patients using antiepileptic drugs, despite similar baseline scores. |
| (Yoshii et al. 2008) | LGG (n=8), HGG (n=23), meningioma (n=34) | Japan | 2008 | Not specified | 31 | Preoperatively, within 1 month of "fully recovering" from surgery, and after radiotherapy (note that not all subjects underwent assessments at all these timepoints) | Surgery (all); some patients appear to have received radiotherapy but the exact numbers are not indicated. | Japanese version of the Modified MMSE | This study evaluated the effects of surgery and the tumour itself on cognition. Cognitive function was normal in patients with right-sided HGG and LGG tumours, but decreased before surgery in patients with left-sided HGGs and did not normalize after surgery. Cognitive performance before and after surgery did not correlate with tumour grade or EOR. |
| (Yuan et al. 2020) | HGG (n=53), LGG (n=77), healthy controls (n=38) | China | 2020 | Prospective cohort | 168 | Prior to surgery | None | MMSE, Aphasia Battery for Chinese Speakers (Chinese standardised adaptation of the Western Aphasia Battery) | This study evaluated the language and control network reorganisation in patients with left-hemisphere HGG and LGG prior to treatment, and included a healthy control comparator group. It found that tumour grade was negatively correlated with language scores and language network integrity on fMRI. Patients with HGGs had significantly lower language scores than those with LGGs, and had more severe language and cingulo-opercular/fronto-parietal network disruptions than healthy controls or patients with LGGs. |
| (Zarino et al. 2020) | WHO grade I/II gliomas (n=9), WHO grade III/IV gliomas (n=26) | Italy | 2020 | Prospective | 35 | Before surgery, a few days after surgery, and 3 months after surgery | Gross total resection (n=14), subtotal resection (n=16), missing data (n=5) | Comprehension (Token Test), verbal production (Object Naming, Verb Naming, Face Recognition, Phonemic Fluency, Semantic Fluency), repetition (Word Repetition, Non-Word Repetition, and Sentence Repetition Tests from the BADA [Batteria per l'analisi dei deficit afasici, Battery for the Aanalysis of Aphasic Disorders] Test) | This study aimed to evaluate for the presence of language impairments before and after surgery in patients with high- and low-grade insular gliomas, and examine the relationship between language impairment and tumour volume preoperatively and extent of resection 3 months postoperatively. The study found that patients with pure left-sided insular lesions had language impairments before and after surgery, with overall, patients with left-sided lesions showing worsening performing immediately after surgery followed by a partial recovery. Tumours that involved not only the insula but the adjacent networks were associated with more severe deficits. There was no correlation found between tumour volume, extent of resection, and language impairment. The authors concluded that the left insular lobe is an important hub in language networks, and surgery for insular gliomas is possible with appropriate planning as well as the use of intraoperative awake mapping. |
| (Zhang et al. 2021) | WHO grade I glioma (n=2), WHO grade II glioma (n=79), WHO grade III glioma (n=28), WHO grade IV glioma (n=28) | China | 2021 | Prospective | 137 | Preoperatively | None | MMSE, Aphasia Battery for Chinese Speakers (Chinese standardised adaptation of the Western Aphasia Battery) | This study evaluated lesion topography data in patients with left-sided HGGs and LGGs involving the language network. Education was significantly correlated with language scores in the HGG group. Tumour location in the left posterior middle temporal gyrus predicted deficits in spontaneous speech and naming scores in the HGG group. Tumour volume was not correlated with language scores in LGG or HGG patients. In relation to the ABC scores, 21% of LGG patients and 50% of HGG patients met the clinical criterion for a diagnosis of aphasia. In a multivariate analysis controlling for age, sex, education, tumour volume and imaging collection site, AQ, spontaneous speech, comprehension, and naming scores were still significantly lower in the HGG group compared to the LGG group. The authors stated that a lack of significant lesion-language mapping results in the LGG group indicated large functional reorganisation in this population group, and highlights the importance of glioma grade in macrostructural plasticity mechanisms that modulate brain-behaviour relationships. |
| (Zimmermann et al. 2020) | WHO grade IV glioma (n=4), WHO grade III anaplastic astrocytoma (n=3), WHO grade III anaplastic oligodendroglioma (n=3), WHO grade II diffuse astrocytoma (n=3), WHO grade I pilocytic astrocytoma (n=1), WHO grade I ganglioglioma (n=1), cavernoma (n=2), WHO grade I meningioma (n=1), and 3 healthy controls | Germany and Austria | 2020 | Not specified | 22 | Preoperatively, 1-5 days as well as 6-9 days postoperatively | Surgery (all) | All 5 subpanels of the AAT (Token Test, Speech Repetition, Written Language, Picture Naming, Speech Comprehension) | This study evaluated reorganisation of language-relevant areas of brains affected by lesions (predominantly tumours) using fMRI and magnetoencephalography. The study found that all but one of the 18 patients had intact language function as defined by results in the AAT. One third of the patients developed language deficits in picture-naming experiments 1-5 days postoperatively. Upon re-evaluation, between 6 and 9 days following surgery (after dexamethasone therapy), only 2 (11%) patients had persistent impaired language. Language function reorganisation was identified on fMRI and MEG in 29% of patients. As expected, testing was normal in the healthy controls. |

**Abbreviations**: AAT, Aachen Aphasia Test; APOE, apolipoprotein E; BADS, Behavioural Assessment of the Dysexecutive Syndrome; BASA, Boston Assessment of Severe Aphasia; BBB, blood-brain barrier; BDAE, Boston Diagnostic Aphasia Examination; BFRT, Benton Facial Recognition Test; BLAST, Brief Language Assessment for Surgical Tumor Patients; BNT, Boston Naming Test; BSAT, Brixton Spatial Anticipation Test; BTA, Brief Test of Attention; BVRT, Benton Visual Retention Test; CF, Category Fluency; COWAT, Controlled Oral Word Association Test; CPT, Continuous Performance Test; DNET, dysembryoplastic neuroepithelial tumour; DuLIP, Dutch Linguistic Intraoperative Protocol; EOR, extent of resection; EORTC-QLQ, European Organisation for Research and Treatment of Cancer Quality of Life Questionnaire (C30 [core] and BN20 [brain-specific] modules); FAB, Frontal Assessment Battery; fMRI, functional magnetic resonance imaging; GBM, glioblastoma; GFAP, glial fibrillary acidic protein; HAWIE-R, German version of the Wechsler Intelligence Test Battery; HGG, high-grade glioma; HSCT, Hayling Sentence Completion Test; HVLT(-R), Hopkins Verbal Learning Test(-Revised); IQ, intelligence quotient; KPS, Karnofsky Performance Status; LDSB, Longest Digit Span Backward; LDSF, Longest Digit Span Forward; LGG, low-grade glioma; MAE, Multilingual Aphasia Examination; MEG, magnetoencephalography; MLUw, Mean Length of Utterance Words; MMSE, Mini-Mental State Examination; MoCA, Montreal Cognitive Assessment; MRI, Magnetic Resonance Imaging; NART, National Adult Reading Test; NIHSS, National Institutes of Health Stroke Scale; NOMS, National Outcomes Measurement System; PASAT, Paced Auditory Serial Addition Test; RAVLT, Rey Auditory Verbal Learning Test; RBANS, Repeatable Battery for the Assessment of Neuropsychological Status; RCPM, Raven's Coloured Progressive Matrices; RCT, randomized controlled trial; ROCFT, Rey Osterrieth Complex Figure Test; SAT, Shifting Attention Test; SD, standard deviation; SDC, Symbol Digit Coding; SF-36, Short Form-36; STAI, State-Trait Anxiety Inventory; TMT, Trail Making Test; TMT-A, Trail Making Test-Part A; TMT-B, Trail Making Test-Part B; TTR, Type Token Ratio; VLMT, Verbal Learning and Memory Test; VOSP, Visual Object and Space Perception battery; WHO, World Health Organization; WAIS(-R), Wechsler Adult Intelligence Scale(-Revised); WCST, Wisconsin Cart Sorting Test; WMS(-R), Wechsler Memory Scale(-Revised).

**References**

Allen, D. et al., 2020. Assessing Discrepancies in Neurocognitive and Patient-Reported Measures of Brain Tumor Survivors. *Oncology nursing forum*, 47(1), pp.E1–E12.

Altieri, R. et al., 2019. Glioma surgery: From preservation of motor skills to conservation of cognitive functions. *JOURNAL OF CLINICAL NEUROSCIENCE*, 70, pp.55–60.

Altshuler, D.B. et al., 2019. BDNF, COMT, and DRD2 polymorphisms and ability to return to work in adult patients with low- and high-grade glioma. *Neuro-Oncology Practice*, 6(5), pp.375–385.

Anders, H. et al., 2021. Hemispheric tumor location and the impact on health-related quality of life, symptomatology, and functional performance outcomes in patients with glioma: an exploratory cross-sectional study. *Disabil Rehabil*, 43(10), pp.1446–1452.

Anderson, S.I., Taylor, R. & Whittle, I.R., 1999. Mood disorders in patients after treatment for primary intracranial tumours. *British Journal of Neurosurgery*, 13(5), pp.480–485.

Antonsson, M., Jakola, A., et al., 2018. Post-surgical effects on language in patients with presumed low-grade glioma. *Acta Neurol Scand*, 137(5), pp.469–480.

Antonsson, M., Johansson, C., et al., 2018. Writing fluency in patients with low-grade glioma before and after surgery. *International journal of language & communication disorders*, 53(3), pp.592–604.

Arbula, S. et al., 2020. Focal left prefrontal lesions and cognitive impairment: A multivariate lesion-symptom mapping approach. *Neuropsychologia*, 136, p.107253.

Armstrong, C.L. et al., 2002. Late cognitive and radiographic changes related to radiotherapy: initial prospective findings. *Neurology*, 59(1), pp.40–48.

Armstrong, C.L. et al., 2000. Radiotherapeutic effects on brain function: double dissociation of memory systems. *Neuropsychiatry, neuropsychology, and behavioral neurology*, 13(2), pp.101–111.

Aydinlar, E.I. et al., 2020. Intraoperative motor speech mapping under general anesthesia using long-latency response from laryngeal muscles. *Clinical Neurology and Neurosurgery*, 190, p.105672.

Barzilai, O. et al., 2018. Improvement in cognitive function after surgery for low-grade glioma. *Journal of Neurosurgery*, 130(2), pp.426–434.

Berger, A. et al., 2019. Incidence and impact of stroke following surgery for low-grade gliomas. *Journal of Neurosurgery*, pp.1–9.

Bette, S. et al., 2020. Perioperative neurocognitive functions in patients with neuroepithelial intracranial tumors. *J Neurooncol*, 147(1), pp.77–89.

Blonski, M. et al., 2012. Combination of neoadjuvant chemotherapy followed by surgical resection as a new strategy for WHO grade II gliomas: a study of cognitive status and quality of life. *J Neurooncol*, 106(2), pp.353–366.

Boone, M. et al., 2016. Prevalence and profile of cognitive impairment in adult glioma: a sensitivity analysis. *J Neurooncol*, 129(1), pp.123–130.

Borde, P. et al., 2021. An analysis of neurocognitive dysfunction in brain tumors. *Indian journal of psychiatry*, 63(4), pp.377–382.

Braun, V. et al., 2006. Brain tumour surgery in the vicinity of short-term memory representation--results of neuronavigation using fMRI images. *Acta Neurochir (Wien)*, 148(7), pp.733–739.

Breen, W.G. et al., 2020. Final report from Intergroup NCCTG 86-72-51 (Alliance): a phase III randomized clinical trial of high-dose versus low-dose radiation for adult low-grade glioma. *Neuro-oncology*, 22(6), pp.830–837.

Brennum, J. et al., 2018. Glioma surgery with intraoperative mapping—balancing the onco-functional choice. pp.1–8.

Brown, P.D. et al., 2003. Effects of radiotherapy on cognitive function in patients with low-grade glioma measured by the folstein mini-mental state examination. *Journal of clinical oncology : official journal of the American Society of Clinical Oncology*, 21(13), pp.2519–2524.

Bunevičius, A. et al., 2017. Preoperative low tri-iodothyronine concentration is associated with worse health status and shorter five year survival of primary brain tumor patients. *Oncotarget*, 8(5), pp.8648–8656.

Butterbrod, E. et al., 2021. The APOE ε4 allele in relation to pre- and postsurgical cognitive functioning of patients with primary brain tumors. *Eur J Neurol*, 28(5), pp.1665–1676.

Campanella, F. et al., 2015. Acute effects of surgery on emotion and personality of brain tumor patients: surgery impact, histological aspects, and recovery. *Neuro-oncology*, 17(8), pp.1121–1131.

Campanella, F. et al., 2021. Cognitive reserve protects language functions in patients with brain tumours. *Neuropsychologia*, 154, p.107769.

Campanella, F. et al., 2018. Localizing Memory Functions in Brain Tumor Patients: Anatomical Hotspots over 260 Patients. *World Neurosurgery*, 120, pp.e690–e709.

Campanella, F. et al., 2017. Long-Term Cognitive Functioning and Psychological Well-Being in Surgically Treated Patients with Low-Grade Glioma. *World Neurosurgery*, 103, pp.799–808.e9.

Capo, G. et al., 2020. Cognitive Functions in Repeated Glioma Surgery. *Cancers*, 12(5).

Caramanna, I. et al., 2022. Corticosteroids use and neurocognitive functioning in patients with recurrent glioblastoma: evidence from European Organization for Research and Treatment …. *Neuro-Oncology Practice*.

Cayuela, N. et al., 2019. Cognitive and brain structural changes in long-term oligodendroglial tumor survivors. *Neuro-oncology*, 21(11), pp.1470–1479.

Chang, W.-H., 2018. Intraoperative linguistic performance during awake brain surgery predicts postoperative linguistic deficits. *J Neurooncol*, 139(1), pp.215–223.

Chapman, C.H. et al., 2016. Diffusion tensor imaging predicts cognitive function change following partial brain radiotherapy for low-grade and benign tumors. *Radiother Oncol*, 120(2), pp.234–240.

Correa, D.D. et al., 2018. A pilot study of neuropsychological functions, APOE and amyloid imaging in patients with gliomas. *J Neurooncol*, 136(3), pp.613–622.

Correa, D.D. et al., 2007. Cognitive functions in low-grade gliomas: disease and treatment effects. *Journal of Neuro-Oncology*, 81(2), pp.175–184.

Correa, D.D. et al., 2019. Genetic variants and cognitive functions in patients with brain tumors. *Neuro-oncology*, 21(10), pp.1297–1309.

Correa, D.D. et al., 2008. Longitudinal cognitive follow-up in low grade gliomas. *Journal of Neuro-Oncology*, 86(3), pp.321–327.

Costello, A. et al., 2004. The early effects of radiotherapy on intellectual and cognitive functioning in patients with frontal brain tumours: the use of a new neuropsychological methodology. *Journal of Neuro-Oncology*, 67(3), pp.351–359.

Coşman, M. et al., 2019. Correlations between histological subtypes and neurocognitive assessment of language area tumors. Our 43 case series and review of the literature. *Romanian journal of morphology and embryology = Revue roumaine de morphologie et embryologie*, 60(4), pp.1143–1151.

Douw, L. et al., 2009. Cognitive and radiological effects of radiotherapy in patients with low-grade glioma: long-term follow-up. *Lancet Neurol*, 8(9), pp.810–818.

Fang, D. et al., 2014. Attention dysfunction of postoperative patients with glioma. *World journal of surgical oncology*, 12(1), pp.317–5.

Forster, M.-T. et al., 2020. Benefits of glioma resection in the corpus callosum. *Scientific Reports*, 10(1), pp.16630–10.

Friedman, M.A., Meyers, C.A. & Sawaya, R., 2003. Neuropsychological effects of third ventricle tumor surgery. *Neurosurgery*, 52(4), pp.791–8– discussion 798.

Gehring, K. et al., 2009. Cognitive rehabilitation in patients with gliomas: a randomized, controlled trial. *Journal of Clinical Oncology*, 27(22), pp.3712–3722.

Gehring, K. et al., 2011. Predictors of neuropsychological improvement following cognitive rehabilitation in patients with gliomas. *Journal of the International Neuropsychological Society : JINS*, 17(2), pp.256–266.

Giovagnoli, A.R. et al., 2007. Preserved memory in temporal lobe epilepsy patients after surgery for low-grade tumour. A pilot study. *Neurological sciences : official journal of the Italian Neurological Society and of the Italian Society of Clinical Neurophysiology*, 28(5), pp.251–258.

Goldstein, B. et al., 2003. Attention in adult intracranial tumors patients. *Journal of clinical and experimental neuropsychology*, 25(1), pp.66–78.

Haldbo-Classen, L. et al., 2020. Cognitive impairment following radiation to hippocampus and other brain structures in adults with primary brain tumours. *Radiotherapy and Oncology*, 148, pp.1–7.

Hendriks, E.J. et al., 2018. Linking late cognitive outcome with glioma surgery location using resection cavity maps. *Human brain mapping*, 39(5), pp.2064–2074.

Hoffermann, M. et al., 2017. Pre- and postoperative neurocognitive deficits in brain tumor patients assessed by a computer based screening test. *J Clin Neurosci*, 36, pp.31–36.

Hornak, J. et al., 2003. Changes in emotion after circumscribed surgical lesions of the orbitofrontal and cingulate cortices. *Brain*, 126(Pt 7), pp.1691–1712.

Incekara, F. et al., 2018. Changes in language white matter tract microarchitecture associated with cognitive deficits in patients with presumed low-grade glioma. *Journal of Neurosurgery*, pp.1–9.

Irle, E. et al., 1994. Mood changes after surgery for tumors of the cerebral cortex. *Arch Neurol*, 51(2), pp.164–174.

Jaeckle, K.A. et al., 2021. CODEL: phase III study of RT, RT + TMZ, or TMZ for newly diagnosed 1p/19q codeleted oligodendroglioma. Analysis from the initial study design. *Neuro-oncology*, 23(3), pp.457–467.

Jagaroo, V., Rogers, M.P. & Black, P.M., 2000. Allocentric visuospatial processing in patients with cerebral gliomas: a neurocognitive assessment. *Journal of Neuro-Oncology*, 49(3), pp.235–248.

Jehna, M. et al., 2017. Symmetry of the arcuate fasciculus and its impact on language performance of patients with brain tumors in the language-dominant hemisphere. *Journal of Neurosurgery*, 127(6), pp.1407–1416.

Kaleita, T.A. et al., 2004. Prediction of neurocognitive outcome in adult brain tumor patients. *Journal of Neuro-Oncology*, 67(1-2), pp.245–253.

Kelm, A. et al., 2017. Resection of Gliomas with and without Neuropsychological Support during Awake Craniotomy-Effects on Surgery and Clinical Outcome. *Frontiers in Oncology*, 7, p.176.

Kessels, R.P. et al., 2000. Spatial memory impairment in patients after tumour resection: evidence for a double dissociation. *Journal of Neurology, Neurosurgery & Psychiatry*, 69(3), pp.389–391.

Klein, M. et al., 2001. Neurobehavioral status and health-related quality of life in newly diagnosed high-grade glioma patients. *Journal of clinical oncology : official journal of the American Society of Clinical Oncology*, 19(20), pp.4037–4047.

Klein, M. et al., 2002. Effect of radiotherapy and other treatment-related factors on mid-term to long-term cognitive sequelae in low-grade gliomas: a comparative study. *The Lancet*, 360(9343), pp.1361–1368.

Klein, M. et al., 2003. Epilepsy in low-grade gliomas: the impact on cognitive function and quality of life. *Ann Neurol*, 54(4), pp.514–520.

Klein, M. et al., 2021. Memory in low-grade glioma patients treated with radiotherapy or temozolomide: a correlative analysis of EORTC study 22033-26033. *Neuro-oncology*, 23(5), pp.803–811.

Kleinberg, L., Wallner, K. & Malkin, M.G., 1993. Good performance status of long-term disease-free survivors of intracranial gliomas. *Radiation Oncology Biology*, 26(1), pp.129–133.

Laack, N.N. et al., 2005. Cognitive function after radiotherapy for supratentorial low-grade glioma: a North Central Cancer Treatment Group prospective study. *Radiation Oncology Biology*, 63(4), pp.1175–1183.

Lang, S. et al., 2017. Functional Connectivity in Frontoparietal Network: Indicator of Preoperative Cognitive Function and Cognitive Outcome Following Surgery in Patients with Glioma. *World Neurosurgery*, 105, pp.913–922.e2.

Latini, F. et al., 2021. Role of Preoperative Assessment in Predicting Tumor-Induced Plasticity in Patients with Diffuse Gliomas. *Journal of Clinical Medicine*, 10(5), p.1108.

Lee, A.T. et al., 2020. The impact of high functional connectivity network hub resection on language task performance in adult low- and high-grade glioma. *Journal of Neurosurgery*, 134(3), pp.1102–1112.

Leonetti, A. et al., 2021. Factors Influencing Mood Disorders and Health Related Quality of Life in Adults With Glioma: A Longitudinal Study. *Frontiers in Oncology*, 11, p.662039.

Leroy, H.-A. et al., 2021. Microsurgical resection of fronto-temporo-insular gliomas in the non-dominant hemisphere, under general anesthesia using adjunct intraoperative MRI and no cortical and subcortical mapping: a series of 20 consecutive patients. *Scientific Reports*, pp.1–11.

Liu, Y. et al., 2015. Genetic Modulation of Neurocognitive Function in Glioma Patients. *Clinical cancer research : an official journal of the American Association for Cancer Research*, 21(14), pp.3340–3346.

Liu, D. et al., 2021. Contralesional homotopic functional plasticity in patients with temporal glioma. *Journal of Neurosurgery*, 134, pp.417–425.

Loit, M.-P. et al., 2019. Hotspots of small strokes in glioma surgery: an overlooked risk?, pp.1–8.

MacPherson, S.E. et al., 2017. Cognitive reserve and cognitive performance of patients with focal frontal lesions. *Neuropsychologia*, 96(C), pp.19–28.

MacPherson, S.E. et al., 2020. Cognitive Reserve Proxies Do Not Differentially Account for Cognitive Performance in Patients with Focal Frontal and Non-Frontal Lesions. *Journal of the International Neuropsychological Society : JINS*, 26(8), pp.739–748.

Mandonnet, E., 2019. Transopercular Resection of IDH-Mutated Insular Glioma: A Critical Appraisal of an Initial Experience. *World Neurosurgery*, 132, pp.e563–e576.

Mandonnet, E. et al., 2015. Initial experience using awake surgery for glioma: oncological, functional, and employment outcomes in a consecutive series of 25 cases. *Neurosurgery*, 76(4), pp.382–9– discussion 389.

Maschio, M. et al., 2020. Perampanel in brain tumor-related epilepsy: Observational pilot study. *Brain and behavior*, 10(6), p.e01612.

Mattavelli, G. et al., 2019. Consequences of brain tumour resection on emotion recognition. *Journal of neuropsychology*, 13(1), pp.1–21.

Motomura, K. et al., 2019. Neurocognitive and functional outcomes in patients with diffuse frontal lower-grade gliomas undergoing intraoperative awake brain mapping. *Journal of Neurosurgery*, 132(6), pp.1683–1691.

Motomura, K. et al., 2018. Supratotal Resection of Diffuse Frontal Lower Grade Gliomas with Awake Brain Mapping, Preserving Motor, Language, and Neurocognitive Functions. *World Neurosurgery*, 119, pp.30–39.

Muto, J. et al., 2018. Functional-Based Resection Does Not Worsen Quality of Life in Patients with a Diffuse Low-Grade Glioma Involving Eloquent Brain Regions: A Prospective Cohort Study. *World Neurosurgery*, 113, pp.e200–e212.

Nakada, M. et al., 2020. Awake surgery for right frontal lobe glioma can preserve visuospatial cognition and spatial working memory. *J Neurooncol*, 151(2), pp.221–230.

Nakajima, R. et al., 2017. Damage of the right dorsal superior longitudinal fascicle by awake surgery for glioma causes persistent visuospatial dysfunction. *Scientific Reports*, pp.1–10.

Nakajima, R. et al., 2019. Glioma surgery under awake condition can lead to good independence and functional outcome excluding deep sensation and visuospatial cognition. *Neuro-Oncology Practice*, 6(5), pp.354–363.

Ng, S. et al., 2020. Neuropsychological assessments before and after awake surgery for incidental low-grade gliomas. *Journal of Neurosurgery*, pp.1–10.

Noll, K.R. et al., 2015. Relationships between tumor grade and neurocognitive functioning in patients with glioma of the left temporal lobe prior to surgical resection. *Neuro-oncology*, 17(4), pp.580–587.

Noll, K.R. et al., 2021. Alterations in Functional Connectomics Associated With Neurocognitive Changes Following Glioma Resection. *Neurosurgery*, 88(3), pp.544–551.

Noll, K.R. et al., 2016. Neurocognitive functioning in patients with glioma of the left and right temporal lobes. *J Neurooncol*, 128(2), pp.323–331.

Noll, K.R. et al., 2017. Relationships between neurocognitive functioning, mood, and quality of life in patients with temporal lobe glioma. *Psycho-oncology*, 26(5), pp.617–624.

Norrelgen, F., Jensdottir, M. & Östberg, P., 2020. High-level language outcomes three and twelve months after awake surgery in low grade glioma and cavernoma patients. *Clinical Neurology and Neurosurgery*, 195, p.105946.

Owen, A.M. et al., 1996. Double dissociations of memory and executive functions in working memory tasks following frontal lobe excisions, temporal lobe excisions or amygdalo-hippocampectomy in man. *Brain*, 119 ( Pt 5), pp.1597–1615.

Pallud, J. & Dezamis, E., 2017. Functional and oncological outcomes following awake surgical resection using intraoperative cortico-subcortical functional mapping for supratentorial gliomas located in eloquent areas. *Neuro-Chirurgie*, 63(3), pp.208–218.

Peiffer, A.M. et al., 2013. Neuroanatomical target theory as a predictive model for radiation-induced cognitive decline. *Neurology*, 80(8), pp.747–753.

Peper, M. & Irle, E., 1997. Categorical and dimensional decoding of emotional intonations in patients with focal brain lesions. *Brain and language*, 58(2), pp.233–264.

Postma, T.J. et al., 2002. Radiotherapy-induced cerebral abnormalities in patients with low-grade glioma. *Neurology*, 59(1), pp.121–123.

Prabhu, R.S. et al., 2014. Effect of the addition of chemotherapy to radiotherapy on cognitive function in patients with low-grade glioma: secondary analysis of RTOG 98-02. *Journal of Clinical Oncology*, 32(6), pp.535–541.

Prat-Acín, R. et al., 2021. Intraoperative brain mapping of language, cognitive functions, and social cognition in awake surgery of low-grade gliomas located in the right non-dominant hemisphere. *Clinical Neurology and Neurosurgery*, 200, p.106363.

Quiñones, I. et al., 2021. What Can Glioma Patients Teach Us about Language (Re)Organization in the Bilingual Brain: Evidence from fMRI and MEG. *Cancers*, 13(11), p.2593.

Racine, C.A. et al., 2015. Neurocognitive Function in Newly Diagnosed Low-grade Glioma Patients Undergoing Surgical Resection With Awake Mapping Techniques. *Neurosurgery*, 77(3), pp.371–9– discussion 379.

Raysi Dehcordi, S. et al., 2013. Cognitive deficits in patients with low and high grade gliomas. *J Neurosurg Sci*, 57(3), pp.259–266.

Reijneveld, J.C. et al., 2001. Cognitive status and quality of life in patients with suspected versus proven low-grade gliomas. *Neurology*, 56(5), pp.618–623.

Rijnen, S.J.M. et al., 2019. Cognitive functioning in patients with low-grade glioma: effects of hemispheric tumor location and surgical procedure. *Journal of Neurosurgery*, pp.1–12.

Robinson, G.A., Biggs, V. & Walker, D.G., 2015. Cognitive screening in brain tumors: short but sensitive enough? *Frontiers in Oncology*, 5, p.60.

Roman-Goldstein, S. et al., 1995. MR and cognitive testing of patients undergoing osmotic blood-brain barrier disruption with intraarterial chemotherapy. *American Journal of Neuroradiology*, 16(3), pp.543–553.

Romero-Garcia, R. et al., 2021. Memory recovery in relation to default mode network impairment and neurite density during brain tumor treatment. *Journal of Neurosurgery*, pp.1–11.

Rossi, M. et al., 2021. Challenging Giant Insular Gliomas With Brain Mapping: Evaluation of Neurosurgical, Neurological, Neuropsychological, and Quality of Life Results in a Large Mono-Institutional Series. *Frontiers in Oncology*, 11, p.629166.

Rossi, M. et al., 2019. Is supratotal resection achievable in low-grade gliomas? Feasibility, putative factors, safety, and functional outcome. *Journal of Neurosurgery*, 132(6), pp.1692–1705.

Sanai, N., Mirzadeh, Z. & Berger, M.S., 2008. Functional outcome after language mapping for glioma resection. *N Engl J Med*, 358(1), pp.18–27.

Santini, B. et al., 2012. Cognitive outcome after awake surgery for tumors in language areas. *J Neurooncol*, 108(2), pp.319–326.

Sarubbo, S. et al., 2011. Awake surgery in low-grade gliomas harboring eloquent areas: 3-year mean follow-up. *Neurological sciences : official journal of the Italian Neurological Society and of the Italian Society of Clinical Neurophysiology*, 32(5), pp.801–810.

Satoer, D. et al., 2017. Differential Effects of Awake Glioma Surgery in “Critical” Language Areas on Cognition: 4 Case Studies. *Case Reports in Neurological Medicine*, pp.1–10.

Satoer, D. et al., 2014. Long-term evaluation of cognition after glioma surgery in eloquent areas. *J Neurooncol*, 116(1), pp.153–160.

Satoer, D. et al., 2018. Spontaneous speech in patients with gliomas in eloquent areas: Evaluation until 1 year after surgery. *Clinical Neurology and Neurosurgery*, 167, pp.112–116.

Scarone, P. et al., 2009. Agraphia after awake surgery for brain tumor: new insights into the anatomo-functional network of writing. *Surg Neurol*, 72(3), pp.223–41– discussion 241.

Shankar, A. & Rajshekhar, V., 2003. Radiological and clinical outcome following stereotactic biopsy and radiotherapy for low-grade insular astrocytomas. *Neurology India*, 51(4), pp.503–506.

Sherman, J.C. et al., 2015. Neurocognitive effects of proton radiation therapy in adults with low-grade glioma. *J Neurooncol*, 126(1), pp.157–164.

Shih, H.A. et al., 2015. Proton therapy for low-grade gliomas: Results from a prospective trial. *Cancer*, 121(10), pp.1712–1719.

Sunyach, M.P. et al., 2003. Conformal irradiation for pure and mixed oligodendroglioma: the experience of Centre Leon Berard Lyon. *Radiation Oncology Biology*, 56(1), pp.296–303.

Surma-aho, O. et al., 2001. Adverse long-term effects of brain radiotherapy in adult low-grade glioma patients. *Neurology*, 56(10), pp.1285–1290.

Tabrizi, S. et al., 2019. Long-term outcomes and late adverse effects of a prospective study on proton radiotherapy for patients with low-grade glioma. *Radiother Oncol*, 137, pp.95–101.

Talacchi, A. et al., 2011. Cognitive effects of tumour and surgical treatment in glioma patients. *J Neurooncol*, 103(3), pp.541–549.

Taphoorn, M.J. et al., 1994. Cognitive functions and quality of life in patients with low-grade gliomas: the impact of radiotherapy. *Ann Neurol*, 36(1), pp.48–54.

Teixidor, P. et al., 2007. Assessment of verbal working memory before and after surgery for low-grade glioma. *Journal of Neuro-Oncology*, 81(3), pp.305–313.

Tomasino, B. et al., 2020. Phonological and surface dyslexia in individuals with brain tumors: Performance pre-, intra-, immediately post-surgery and at follow-up. *Human brain mapping*, 41(17), pp.5015–5031.

van Dellen, E., de Witt Hamer, P.C., et al., 2012. Connectivity in MEG resting-state networks increases after resective surgery for low-grade glioma and correlates with improved cognitive performance. *NeuroImage. Clinical*, 2, pp.1–7.

van Dellen, E., Douw, L., et al., 2012. MEG network differences between low- and high-grade glioma related to epilepsy and cognition. *PLoS One*, 7(11), p.e50122.

van Dokkum, L.E.H. et al., 2019. Resting state network plasticity related to picture naming in low-grade glioma patients before and after resection. *NeuroImage. Clinical*, 24, p.102010.

van Kessel, E. et al., 2022. Tumor-related molecular determinants of neurocognitive deficits in patients with diffuse glioma. *Neuro-oncology*.

van Kessel, E. et al., 2019. Tumor-related neurocognitive dysfunction in patients with diffuse glioma: a retrospective cohort study prior to antitumor treatment. *Neuro-Oncology Practice*, 6(6), pp.463–472.

Vendrell, P. et al., 1995. The role of prefrontal regions in the Stroop task. *Neuropsychologia*, 33(3), pp.341–352.

Vilkki, J., Levänen, S. & Servo, A., 2002. Interference in dual-fluency tasks after anterior and posterior cerebral lesions. *Neuropsychologia*, 40(3), pp.340–348.

Vogt, V.L. et al., 2018. Cognitive features and surgical outcome of patients with long-term epilepsy-associated tumors (LEATs) within the temporal lobe. *Epilepsy & Behavior*, 88(C), pp.25–32.

Wang, Y. et al., 2021. Comprehensive ability evaluation and trend analysis of patients with malignant intracranial tumors in the perisurgery period. *Brain and behavior*, 11(11), p.e02192.

Wefel, J.S. et al., 2016. Neurocognitive function varies by IDH1 genetic mutation status in patients with malignant glioma prior to surgical resection. *Neuro-oncology*, 18(12), pp.1656–1663.

Weniger, G. & Irle, E., 2002. Impaired facial affect recognition and emotional changes in subjects with transmodal cortical lesions. *Cerebral cortex (New York, N.Y. : 1991)*, 12(3), pp.258–268.

Wolf, J. et al., 2016. Evaluation of neuropsychological outcome and “quality of life” after glioma surgery. *Langenbeck's Archives of Surgery*, pp.1–9.

Wu, A.S. et al., 2011. Neurocognitive function before and after surgery for insular gliomas. *Journal of Neurosurgery*, 115(6), pp.1115–1125.

Yavas, C. et al., 2012. Prospective assessment of health-related quality of life in patients with low-grade glioma: a single-center experience. *Supportive care in cancer : official journal of the Multinational Association of Supportive Care in Cancer*, 20(8), pp.1859–1868.

Yoshii, Y. et al., 2008. Cognitive function of patients with brain tumor in pre- and postoperative stage. *Surg Neurol*, 69(1), pp.51–61– discussion 61.

Yuan, B. et al., 2020. Tumor grade-related language and control network reorganization in patients with left cerebral glioma. *Cortex; a journal devoted to the study of the nervous system and behavior*, 129, pp.141–157.

Zarino, B. et al., 2020. Insular lobe surgery and cognitive impairment in gliomas operated with intraoperative neurophysiological monitoring. pp.1–11.

Zhang, N. et al., 2021. Multivariate machine learning-based language mapping in glioma patients based on lesion topography. *Brain imaging and behavior*, 15(5), pp.2552–2562.

Zimmermann, M. et al., 2020. Refined Functional Magnetic Resonance Imaging and Magnetoencephalography Mapping Reveals Reorganization in Language-Relevant Areas of Lesioned Brains. *World Neurosurgery*, 136, pp.e41–e59.
